# Beyond the SAFE strategy: Systematic review and meta-analysis of prevalence and associated factors of active trachoma among children in Ethiopia

**DOI:** 10.1371/journal.pone.0312024

**Published:** 2025-02-20

**Authors:** Zufan Alamrie Asmare, Denekew Tenaw Anley, Melaku Ashagrie Belete, Anteneh Mengist Dessie, Melkamu Aderajew Zemene, Ermiyas Alemayehu, Natnael Moges, Natnael Kebede, Sintayehu Simie Tsega, Asaye Alamneh Gebeyehu, Rahel Mulatie Anteneh, Ermias Sisay Chanie

**Affiliations:** 1 Department of Ophthalmology, School of Medicine and Health Science, Debre Tabor University, Debre Tabor, Ethiopia; 2 Department of Public Health, College of Health Sciences, Debre Tabor University, Debre Tabor, Ethiopia; 3 Department of Medical Laboratory Sciences, College of Medicine and Health Sciences, Wollo University, Dessie, Ethiopia; 4 Department of Pediatrics and Child Health Nursing, College of Health Sciences, Debre Tabor University, Debre Tabor, Ethiopia; 5 Department of Health Promotion, School of Public Health College of Medicine Health Sciences, Wollo University, Dessie, Ethiopia; 6 Department of Medical Nursing, School of Nursing, College of Medicine and Health Science, University of Gondar, Gondar, Ethiopia; 7 Department of Pediatric and Child Health, Debre Tabor University, Debre Tabor, Ethiopia; Cranfield University, UNITED KINGDOM OF GREAT BRITAIN AND NORTHERN IRELAND

## Abstract

**Background:**

Although the Surgery, Antibiotic, facial cleanliness, and environmental improvement (SAFE) strategy was adopted in Ethiopia over the last five years, there is still a high incidence of trachoma in areas with poor hygiene. In Ethiopia, a systematic review and meta-analysis were conducted before the implementation of SAFE implying, a need for the update. Therefore, this review gives the updated pooled prevalence and associated factors of active trachoma in Ethiopia after the implementation of SAFE.

**Method:**

The literature search was performed from PubMed, Google Scholar, EMBASE, HINARI, Scopus, and Web of Sciences from January 1–30, 2024. Data were extracted by using a pre-tested and standardized data extraction format and analyzed using STATA 17 statistical software. I^2^ tests to appraise the heterogeneity across the included studies, a random-effect model to estimate the pooled prevalence, and a sub-group analysis to discern the viable source of heterogeneity were executed. Potential publication bias was also assessed by funnel plot, Egger’s weighted correlation, and Begg’s regression. The odds ratio with its 95% confidence was used to reckon the association between the prevalence and factors.

**Result:**

From 504 identified studies, 20 articles were included. After the SAFE intervention, the national pooled prevalence of active trachoma among children was 21.16% (95% CI 17.28, 25.04). Fly-eye contact(Adjusted odds ratio (AOR) = 3.83, 95% CI: 2.25, 6.52), facial uncleanliness(AOR = 5.48, 95% CI: 3.02, 9.96), non-utilization of latrine (AOR = 3.30, 95% CI: 2.10, 5.18), and retrieving water from river(AOR = 2.94; 95%CI: 1.42, 6.05) were significantly associated with active trachoma.

**Conclusion:**

In Ethiopia, the pooled prevalence of active trachoma after SAFE intervention was much higher than the World Health Organization (WHO) threshold prevalence. It continues to pose a significant public health concern and is far from the elimination of trachoma as a public health problem. Fly-eye contact, facial cleanliness, latrine utilization, and source of water increase the odds of active trachoma. Therefore, it is imperative to fine-tune the intervention focus on personal hygiene-related activities in removing dirt, fly-eye contact, and a well-structured approach for both constructing and ensuring the functionality of household taps and latrines. Additionally, It is crucial to initiate a reliable SAFE intervention in Ethiopia.

## Introduction

Trachoma, the neglected and principal cause of preventable blindness, is caused by the bacteria *Chlamydia trachomatis* [1, 2]. It spreads from the infected eyes via fingers, eye-seeking flies, or clothing to non-infected eyes [3, 4]. Children under nine are particularly vulnerable to this disease, with prevalence rates ranging between 60 and 90% [5, 6]. According to the 2023 WHO report, globally 115.7 million people live in trachoma-endemic areas and are at risk of blindness. Of these, 88% live in Africa, and half in Ethiopia [7]. The World Health Organization (WHO) estimates that trachoma is responsible for the visual impairment of approximately 1.9 million people globally, with the highest burden in sub-Saharan Africa [8].

Trachoma remains a persistent public health problem, particularly in regions with poor hygiene and among disadvantaged and marginalized populations [3, 9]. Despite several efforts being made to control the disease, including Surgery, Antibiotics, facial cleanliness, and environmental improvement (SAFE) strategy, Africa remains the continent that is most affected by it [9]. The prevalence of active trachoma in Africa ranges from 6% to 45% with a peak prevalence reported in the sub-Saharan region [7, 10]. However, studies focusing after SAFE intervention (2019–2024) showed that Ethiopia had a wide variation in the prevalence of active trachoma ranging between 6% [11] and 44.1% [12].

The effects of trachoma extend beyond physical health, impacting socioeconomic conditions and quality of life [13]. Blindness or severe visual impairment from trachoma can prevent individuals from performing daily tasks, pursuing education, and maintaining employment, thereby perpetuating cycles of poverty [13, 14]. The prevalence of active trachoma in Ethiopia has been the subject of various epidemiological studies. Although systematic reviews about the prevalence of active trachoma were conducted in Ethiopia, some were done before the implementation of SAFE, and the other conducted after the implementation pooled all articles before and after SAFE, implying a need for the update to assess the effectiveness of SAFE in the country. Therefore, this systematic review and meta-analysis gives the updated pooled prevalence and associated factors of active trachoma in Ethiopia after the implementation of SAFE. Documenting the updated pooled prevalence and associated factors after the intervention provides solid evidence for assessing the action plan of WHO trachoma elimination by 2030. Besides, individuals with trachoma face the risk of developing visual complications, so estimating the updated pooled prevalence and associated factors of active trachoma becomes pressing for formulating healthcare strategies and tackling the nation’s penury for introducing policies and developing programs that address the unique challenges posed by active trachoma. Hence, we conducted this systematic review and meta-analysis with the aim of estimating the updated pooled prevalence and associated factors of active trachoma among children in Ethiopia.

## Materials and methods

### Design and searching strategies

This systematic review and meta-analysis were performed to compile the most recent shreds of evidence using articles published and grey literature on the prevalence and associated factors of active trachoma among children in Ethiopia. We searched articles related to this subject across various databases including PubMed/Medline, EMBASE, HINARI, Scopus, Google Scholar, and Web of Science. We also look for grey literature deposited at universities and research institutes’ websites and online repositories. In addition, a manual search was conducted in the Ethiopian Journal of Health Sciences, the Ethiopian Medical Journal, the Ethiopian Journal of Health and Development, and the Ethiopian Journal of Health and Biomedical Sciences. Other important articles unavailable with database and grey literature searches were identified by scanning the reference lists of published articles. For PubMed, we used a combination of key concepts (active trachoma, children, Ethiopia) according to the Medical Subject Headings (MeSH) thesaurus. A search combination was then adapted for other databases. The article searches were conducted independently by two groups of authors, the first group including ZAA, ESC, DTA, and RMA, and the second group including MAB, AMD, MAZ, and EA. The search of articles was done on January 1-30/2024 using the following search combinations; “Active Trachoma"[Title/Abstract] OR "Trachoma "[Title/Abstract]) AND (Children [Title/Abstract] OR School Ages [Title/Abstract]) AND (Ethiopia [Title/Abstract]). We adhered to the protocol of the Preferred Reporting Items for Systematic Reviews and Meta-Analysis (PRISMA) guideline for reporting [15] (S1 File). The protocol was registered in the International Prospective Register of Systematic Reviews (PROSPERO) with registration number CRD42024575043.

### Eligibility criteria

#### Inclusion criteria

*Study area*. Only those studies that were carried out in Ethiopia were included.

*Study design*. All observational studies reporting the prevalence and associated factors of active trachoma among children in Ethiopia were eligible.

*Language*. Articles written only in the English language were considered.

*Population*. Studies done among children (age < 15) were considered.

*Publication year*. All research reports published from 2019 to January 30, 2024, were included.

#### Exclusion criteria

All citations without abstract and/or full text were excluded.

### Study selection and quality appraisal

The collected articles were imported into EndNote X7 software. Following this, any duplicate articles were identified and eliminated. Subsequently, the titles and abstracts of the articles were independently reviewed and anonymized by the first two separate teams of authors. Any disagreements or conflicts between the two teams were resolved through discussion by a third team of review authors (NM, NK, AAG, and SST).

The full text of articles deemed eligible from the title and abstract screening were evaluated for inclusion in the systematic review and meta-analysis (S4 Table). The quality of these studies was assessed using the JBI critical appraisal checklist, which has 9 criteria to evaluate prevalence studies and 8 for analytical cross-sectional studies [16, 17] (S5A and S5B Tables in S5 Table). Articles that achieved an overall quality assessment score of 50% or more were included. The selection process was visually represented using PRISMA flow diagrams.

### Study aims

In this review, the primary aim was to assess the prevalence of active trachoma (trachomatous inflammation follicular and/or trachomatous inflammation intense) among children and the secondary aim was to assess factors associated with active trachoma in children. Odds ratios were used to examine the association between active trachoma and the factors.

### Data extraction

Two sets of review authors independently extracted all the pertinent data using a standardized data extraction template. Dissent between the two sets of authors was consulted with a third group to reach an agreement. The data extraction form was conducted using Microsoft Excel (S2 File) and contains, the name of the first author, year of publication, study year, study area/region, study design, sample size, response rate, prevalence of active trachoma (S2 Table), as well as an adjusted odds ratio (AOR) with a 95% confidence interval (S3A-S3D Tables in S3 Table). The authors addressed any missing data through contact with authors or co-authors of the article and as a result, sufficient information is obtained.

### Data analysis

STATA version 17 was used to analyze the elicited data from a Microsoft Excel spreadsheet. Heterogeneity across studies was assessed using the inverse variance (I2) test, in which 25%, 50%, and 75% correspond to low, moderate, and high respectively [18]. A random effects model was used to calculate the pooled effect according to Der Simonian and Laird’s method, which showed significant heterogeneity between studies. To identify the viable source of heterogeneity, we performed a subgroup analysis based on the region of the study. Potential publication bias was also assessed by descriptive funnel plot, Egger’s weighted correlation, and Begg’s regression intercept tests at a 5% significance level. The odds ratio with its 95% confidence was used to reckon the association between the prevalence of active trachoma among children and factors. The results were delineated as text and Forest plots.

## Result

### Searching results

**Fig 1:**
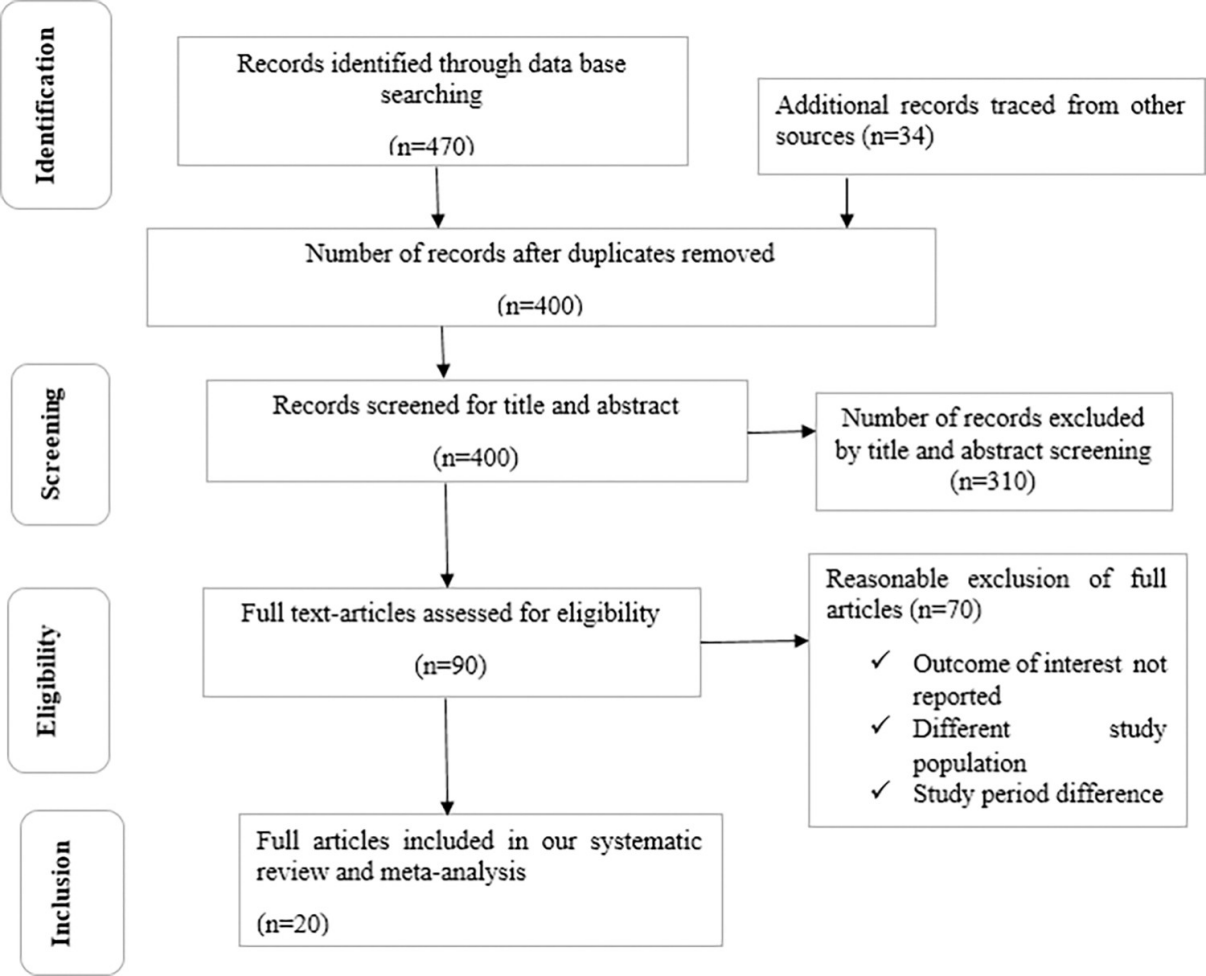
Flow-chart diagram describing the selection of studies for the systematic review and meta-analysis of prevalence and associated factors of active trachoma among children in Ethiopia.

Through electronic searches, 504 studies were discovered (S1 Table), 470 of which were found via database searches and 34 from other sources (academic library repositories). Duplication led to the removal of 104 studies. After screening the title and abstract, 310 were ineligible for this systematic review and meta-analysis and were thus excluded. The final 90 full-text articles were assessed based on predefined criteria for eligibility. Of these, 70 were further excluded for the reasons (study population difference, the outcome of interest was not reported, and study period difference). In the end, 20 studies fulfilled the eligibility requirements and were incorporated into this systematic review and meta-analysis (Fig 1).

### Description of the included studies

As shown in Table 1, this systematic review and meta-analysis included 20 studies. A total of 44,774 children were involved to estimate the pooled prevalence and associated factors of active trachoma. All the studies were cross-sectional and executed in various regions of the country. The prevalence of active trachoma among children in Ethiopia was reported between 6% and 44.1%. Before the analysis, independent reviewers re-evaluated the articles and assigned a quality score of 50% or higher. The following table offers an in-depth description of the studies included (Table 1).

**Table 1:** Description of cross-sectional studies included in this systematic review and meta-analysis.

Author	Year of publication	Region	Sample size	Response rate (%)	Prevalence rate (%)	Quality score (%)
Melkie et al. [19]	2020	Amhara	690	98.30	8.30	75.00
Asmare et al. [20]	2023	Amhara	585	92.30	35.37	87.50
Tuke et al. [21]	2023	SNNP	538	99.60	29.20	87.50
Getachew et al. [12]	2023	SNNP	1292	97.87	44.10	87.50
Genet et al. [11]	2022	Amhara	704	97.80	6.00	75.00
Alambo et al. [22]	2020	SNNP	586	96.20	37.90	75.00
Mekonnen et al. [23]	2022	SNNP	178	95.60	21.91	87.50
Shimelash et al. [24]	2022	Amhara	401	98.25	9.90	75.00
Belsti et al. [25]	2021	SNNP	620	98.39	21.6	75.00
Abdilwohab et al. [26]	2020	SNNP	831	91.60	17.80	75.00
Ayelgn et al. [27]	2021	Amhara	792	94.90	11.80	87.50
Kedir et al. [28]	2021	SNNP	589	95.20	29.40	75.00
Abdurahmanl [29]	2023	Oromia	1211	97.20	22.10	87.50
Yeshitila et al. [30]	2022	Harari	760	97.4	27.00	75.00
Delelegn et al [31]	2021	Oromia	746	94.00	17.50	75.00
Kassaw et al [32]	2020	Tigray	596	100.00	22.00	87.50
Reda et al. [33]	2020	Tigray	502	98.50	21.50	75.00
Seyum et al. [34]	2022	SNNP	1082	NR	20.00	75.00
Miecha et al. [35]	2023	Oromia	29230	NR	10.10	87.50
Nash et al. [36]	2023	Amhara	2841	100	11.90	75.00

Note: SNNP: Southern Nations Nationalities and Peoples Region, NR: Not Rated

### Meta-analysis on the pooled prevalence of active trachoma among children in Ethiopia

**Fig 2:**
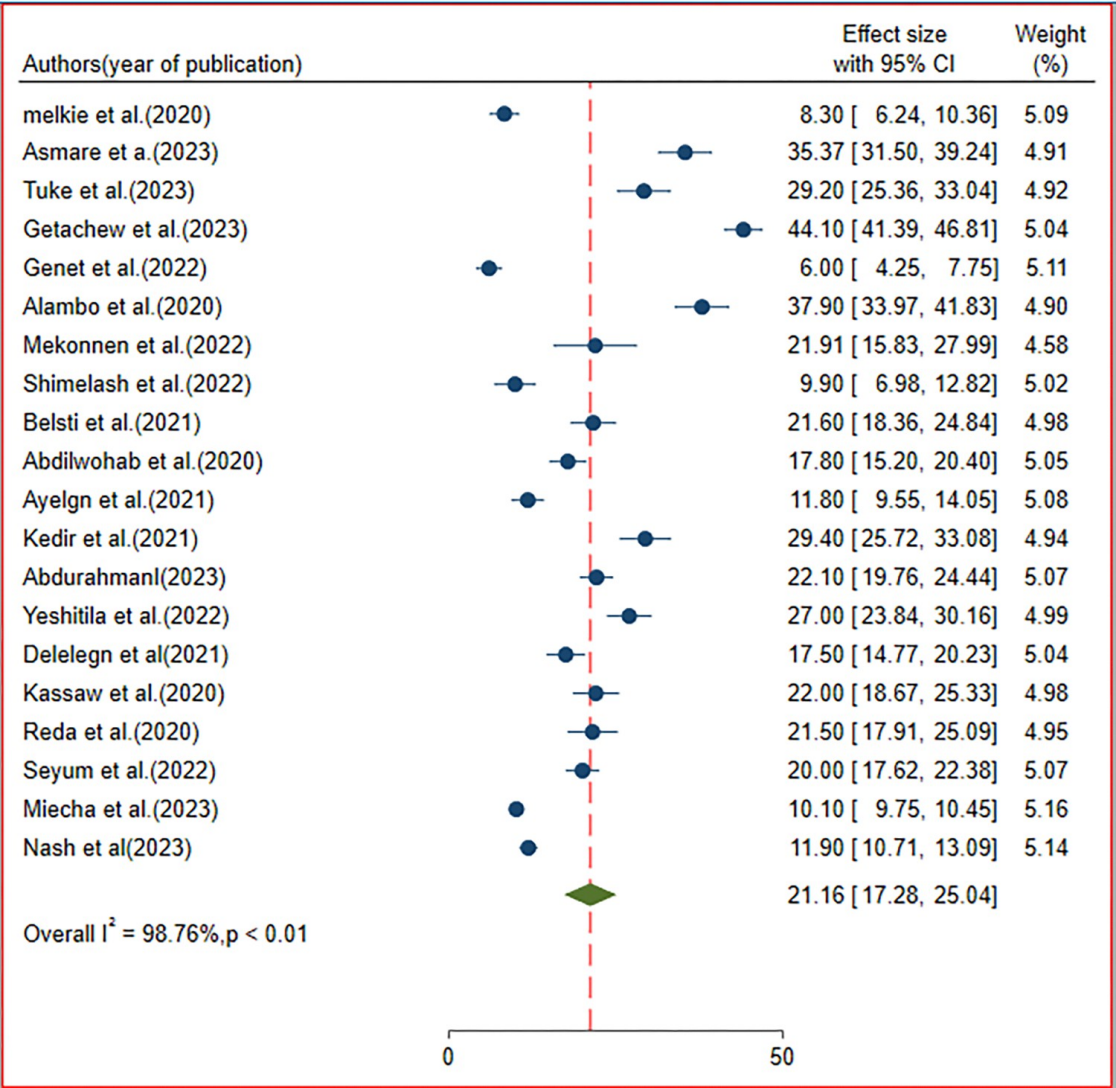
Forest plot of the pooled prevalence of active trachoma among children in Ethiopia.

After SAFE implementation, the pooled prevalence of active trachoma among children in Ethiopia was 21.16% (95% CI 17.28, 25.04). The I^2^ (variation in Effect Size (ES)) attributable to heterogeneity) test result indicated a substantial heterogeneity with I^2^ = 98.76%; at p < 0.01. Therefore we have employed the random effect model to adjust for heterogeneity. The finding was presented in a forest plot (Fig 2).

### Publication bias

**Fig 3:**
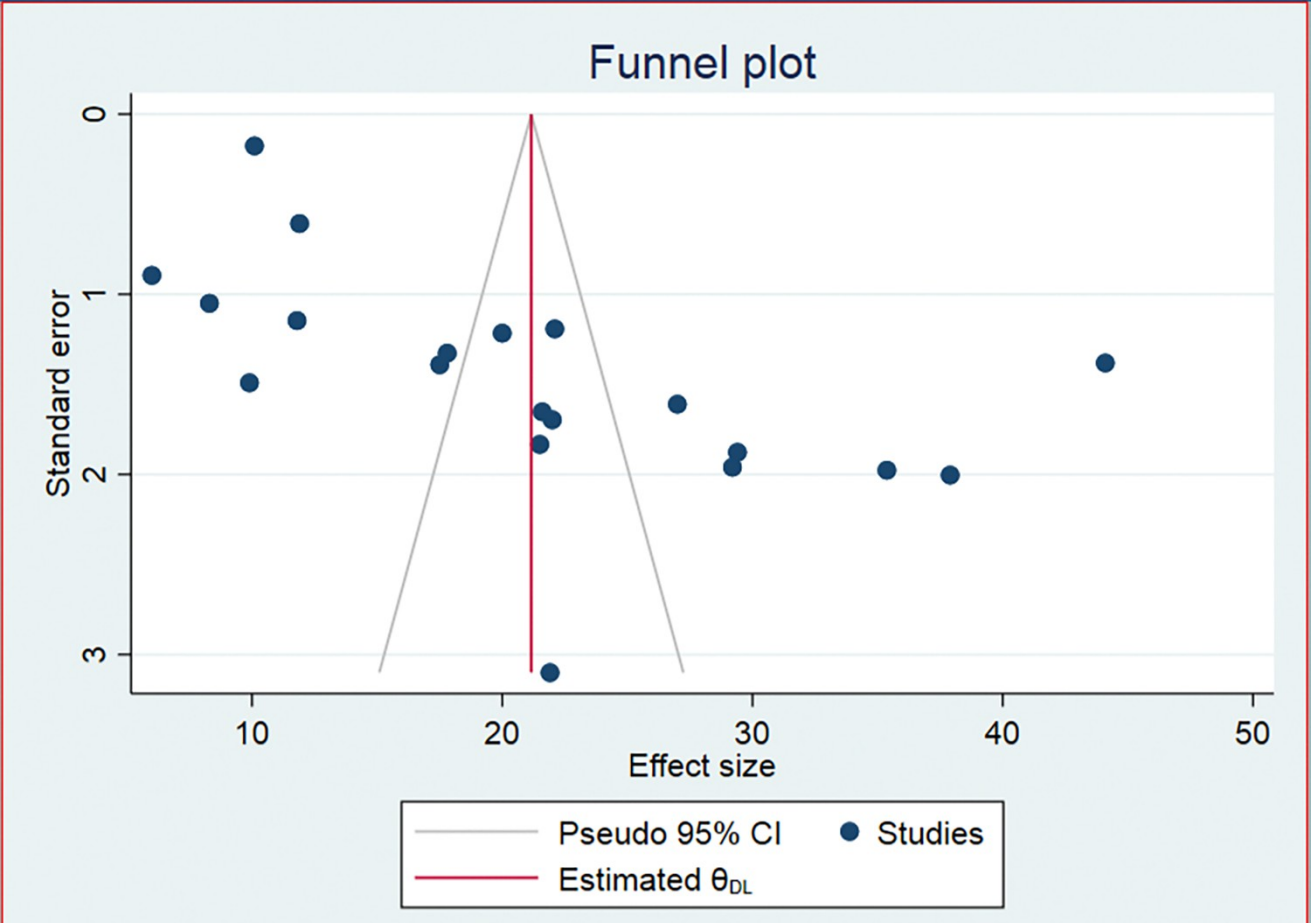
Funnel plot of the studies included in the systematic review and meta-analysis of prevalence and associated factors of active trachoma among children in Ethiopia.

Visual scrutiny of the symmetrical funnel plot (Fig 3) showed no publication bias, which was statistically supported by Egger’s test (P = 0.13) Table 2). Consequently, there was no need to use the Duval and Tweedie nonparametric/ trim and fill method to account for publication bias.

**Table 2:** Egger’s test of the studies included in this systematic review and meta-analysis.

Std-Eff	Coefficient	Std-err	t	p>|t|	Confidence interval	
Slope	8.23	1.26	6.50	0.13	5.57	10.89
Bias	8.47	1.87	4.52	0.32	4.53	12.41

Note: P = 0.13, Std-Eff: Standard effect, Std-err: Standard error

### Subgroup analysis

**Fig 4:**
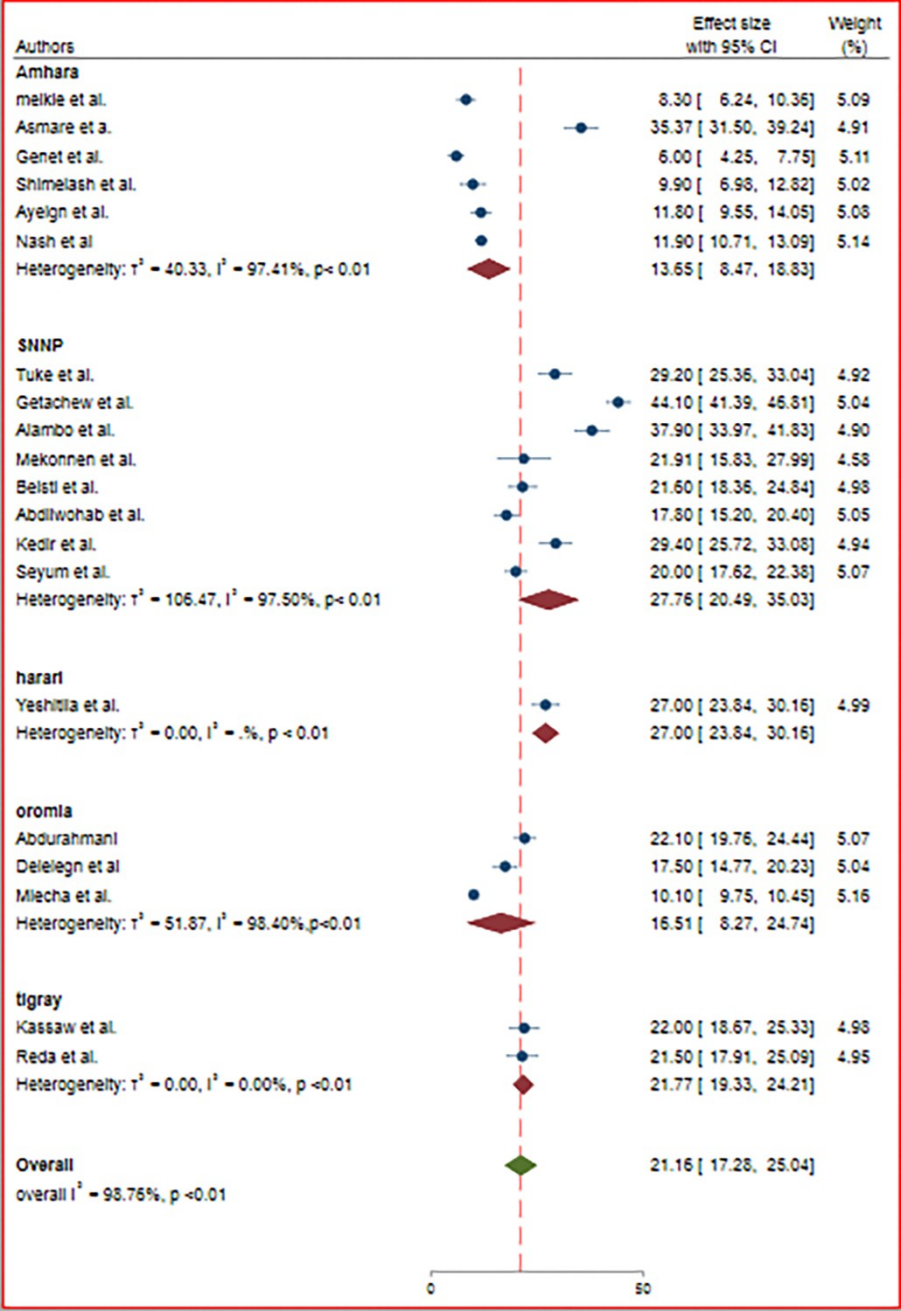
Forest plot of the pooled prevalence of active trachoma among children in Ethiopia by region.

Given the heterogeneity of the studies, we performed a subgroup analysis based on the region where the studies were conducted. Accordingly, the highest prevalence was reported in the SNNP region with a prevalence of 27.76% (95% CI: 20.49, 35.03) and lowest in the Amhara region with a prevalence of 13.65% (95% CI: 8.47, 18.83) (Fig 4).

### Associated factors of active trachoma among children in Ethiopia

In this study, we characterized factors associated with active trachoma among children. A separate analysis was conducted for each factor, which was cogitated in this meta-analysis. Variables assessed were: fly-eye contact, frequency of face washing, soap used for face washing, facial uncleanliness, non-utilization of latrine, nasal discharge, retrieving water from river, and not utilizing waste disposal pit. From those variables; fly-eye contact, facial uncleanliness, unutilization of the latrine, and retrieving water from the river were significantly associated with active trachoma.

Because of the presence of heterogeneity, a random effects model was used to examine the association between the factors and the pooled estimate of active trachoma, and Eggers’s test was used to assess publication bias and denoted no publication bias in all factors.

**Fig 5:**
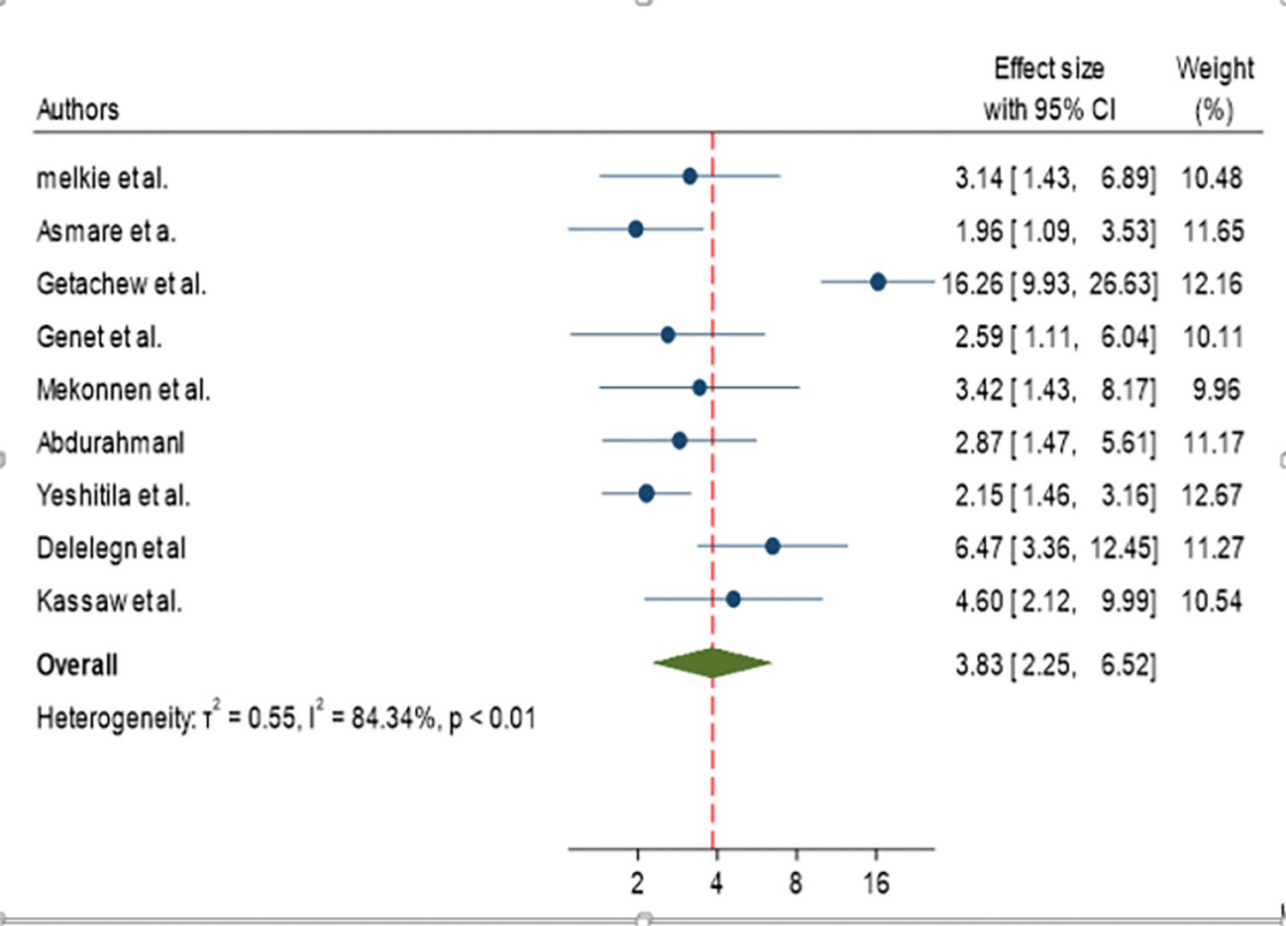
Forest plot showing pooled odds ratio (log scale) of the association between active trachoma and fly-eye contact.

The odds of developing active trachoma for children who had fly-eye contact were 3.83 times (AOR = 3.83, 95% CI: 2.25, 6.52) higher than their counterparts (Fig 5).

**Fig 6:**
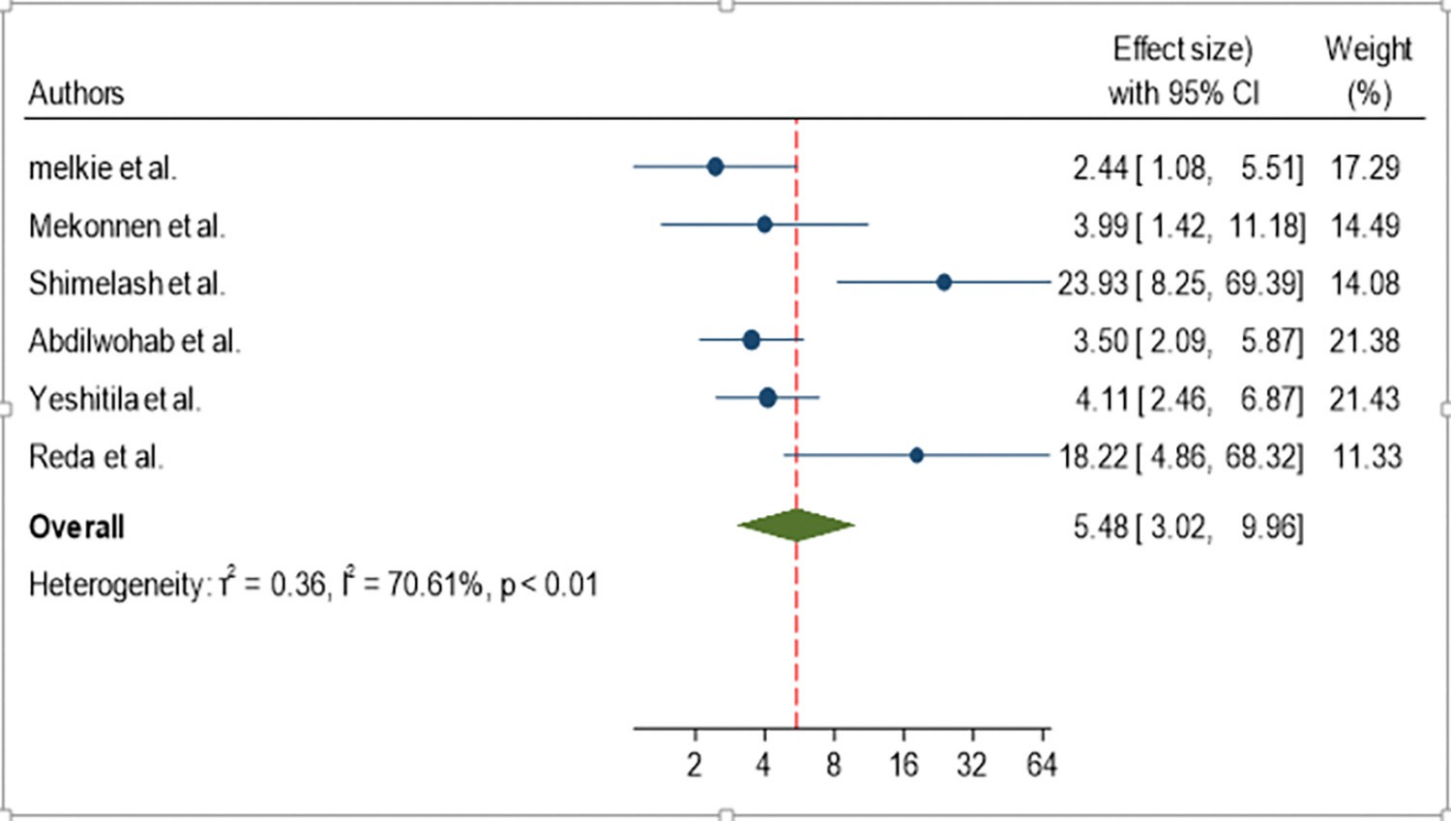
Forest plot showing pooled odds ratio (log scale) of the association between active trachoma and facial uncleanliness.

The odds of developing active trachoma for children with unclean face were 5.48 times (AOR = 5.48, 95% CI: 3.02, 9.96) higher than their counterparts (Fig 6).

**Fig 7:**
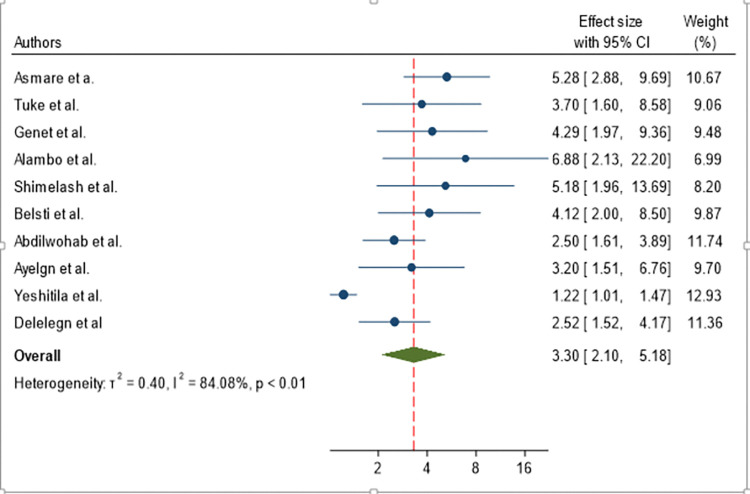
Forest plot showing pooled odds ratio (log scale) of the association between active trachoma and un-utilization of latrine.

The odds of developing active trachoma for children from households who didn’t utilize latrines were 3.30 times (AOR = 3.30, 95% CI: 2.10, 5.18) higher than their counterparts (Fig 7).

**Fig 8:**
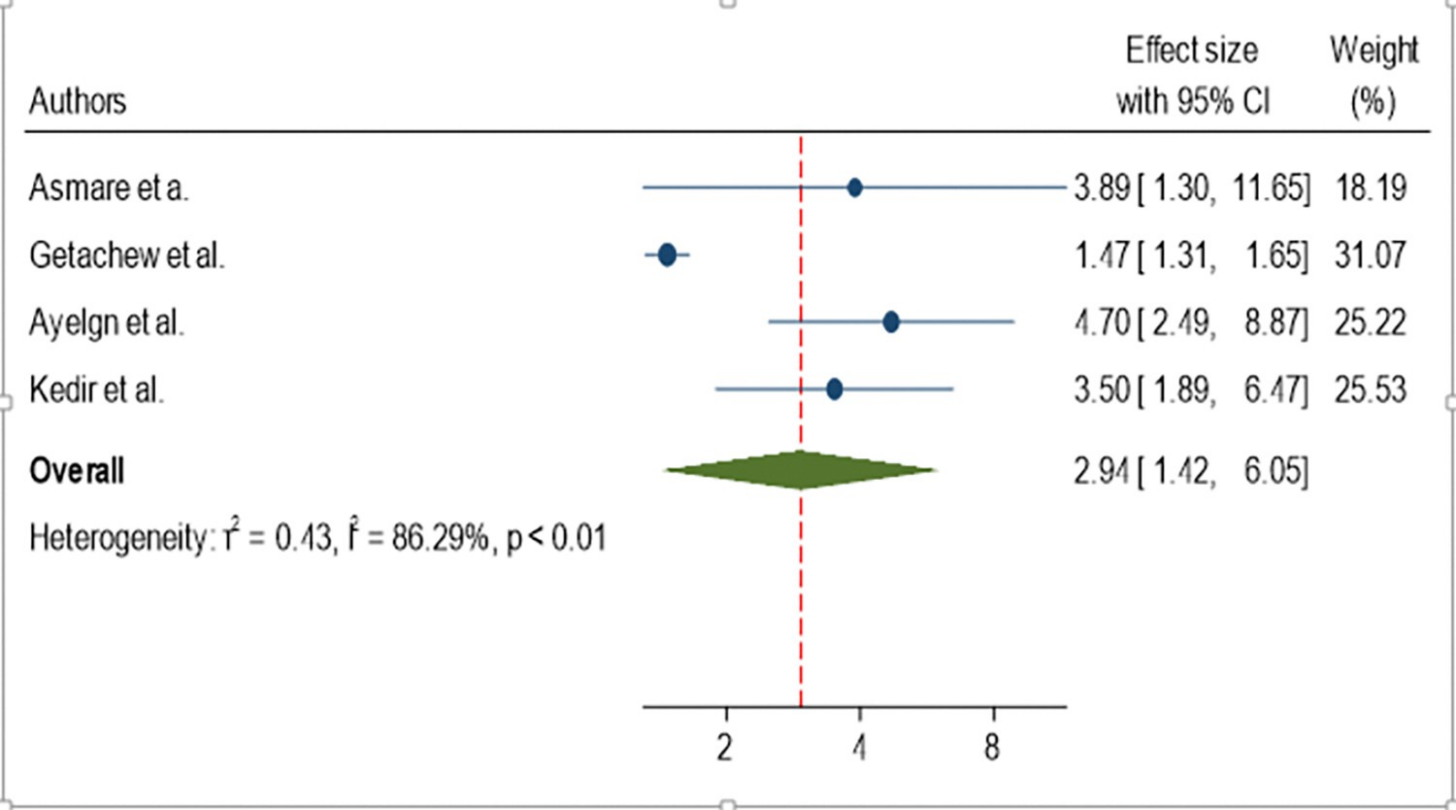
Forest plot showing pooled odds ratio (log scale) of the association between active trachoma and retrieving water from the river.

Regarding the source of water, the odds of developing active trachoma among children from households who get water from the river were 2.94 (AOR = 2.94; 95%CI: 1.42, 6.05) times higher than children from households who get water from the household tap (Fig 8).

## Discussion

Trachoma, a formidable adversary in the battle against preventable blindness, continues to cast a long shadow over underprivileged communities across various African regions, with Ethiopia bearing a significant brunt of this affliction. This study estimated the updated pooled prevalence and associated factors of active trachoma among children in Ethiopia. This meta-analysis estimated that the pooled prevalence of active trachoma among children in Ethiopia was 21.16%. This finding is consistent with a study done in Guinea-Bissau [37]. However, this prevalence of active trachoma was found lower compared to different studies conducted in Mali [38], and Nigeria [39] and higher than studies conducted in the Western division of Fiji [40], Senegal [41], Zimbabwe [42] and Tanzania [43]. These discrepancies might be due to the difference in the nation’s healthcare infrastructure, the effectiveness of trachoma prevention practice, study setting & period, intervention, and other sociodemographic characteristics.

The subgroup analysis of this study also showed that the prevalence of active trachoma among children significantly varies across regions of Ethiopia. The prevalence was higher in children living in SNNP (27.76%) regions as compared to other regions of the country. This highlights a critical paradox: even with the implementation of the SAFE strategy, the persistence of the disease underscores the imperative for a renewed and robust campaign.

Congruous with other studies conducted in Guinea Bissau [37] and Mali [44] in this study, Children who had fly-eye contact were more likely to develop active trachoma than those children who had no fly-eye contact. This might be because of mechanical vectors, flies can contribute to the increased prevalence of trachoma by transmitting the bacteria *Chlamydia trachomatis*.

Facial uncleanliness was another factor in the prevalence of active trachoma among children in which, those children whose faces had not been clean were more likely to have active trachoma as compared to their counterparts. This finding was similar to those of studies done in Gambia and Tanzania [45, 46]. The reason might be due to an unclean face may harbor infectious material and create a favorable environment for the survival and transmission of the bacteria, increasing the risk of person-to-person spread.

We found retrieving water from the river is associated with an increased risk of active trachoma. This finding is supported by studies conducted in different countries [47–49]. This might be due to a river or any exposed water source like ponds and streams can act as a living ground for flies, a reservoir for *Chlamydia trachomatis*. The use of contaminated water for face washing can introduce bacteria, including *Chlamydia trachomatis*, contributing to the transmission of trachoma [20].

Moreover, in this study, the odds of developing active trachoma for those children from households who did not utilize latrines were higher than their counterparts. This is supported by studies done in north-eastern Nigeria, Gunia Bisau, and Fiji [37, 40, 50]. The possible reason might be due to *Musca sorbens* serve as a reservoir for the causative agent and *Chlamydia trachomatis* that has been shown to breed in human excreta [51]. Hence, Engaging in open defecation near residences creates a conducive environment for the breeding of *Musca sorbens* and is a crucial factor in the transmission of *Chlamydia trachomatis* [20, 52].

### Strengths and limitations of the study

To begin with the strength, this systematic review and meta-analysis give updated pooled prevalence and factors affecting active trachoma in Ethiopia after the SAFE intervention. Documenting the updated pooled prevalence and associated factors will help evaluating WHO’s action plan for trachoma elimination by 2030. There was, however, a limitation to this study. It might be necessary to interpret the results with caution since this meta-analysis did not include studies from all Ethiopian regions.

## Conclusion

In this systematic review and meta-analysis, the pooled prevalence of active trachoma after SAFE implementation was much higher than the WHO threshold prevalence (5%). It continues to pose a significant public health concern and is far from the elimination of trachoma as a public health problem, signaling a formidable challenge that continues to linger. Fly-eye contact, facial cleanliness, latrine utilization, and source of water were independent determinants of active trachoma.

Therefore, it is imperative to fine-tune interventions focusing on personal hygiene-related activities such as washing children’s faces utterly to remove dirt, and averting fly-eye contact. Considerable attention and a well-structured approach are vital for both constructing and ensuring the functionality of household taps for personal hygiene. Moreover, it is essential to give priority to the construction and utilization of latrines. It is also crucial to initiate a reliable SAFE intervention in Ethiopia.

## Supporting information

S1 File PRISMA 2020 checklist. (PDF)

S2 File The minimal anonymized data set. (XLSX)

S1 Table All studies identified in the literature search (n = 504). (PDF)

S2 Table Data extraction summary. (PDF)

S3 Table Data extraction summary for each factors. (PDF)

S4 Table Eligibility confirmation. (PDF)

S5 Table Quality assessment by using the JBI critical appraisal tool for analytical and descriptive cross-sectional studies. (PDF)
