# Supplementary material for: Beyond the SAFE strategy: Systematic review and meta-analysis of prevalence and associated factors of active trachoma among children in Ethiopia
Source: PLoS One. 2025 Feb 20;20(2):e0312024. doi: 10.1371/journal.pone.0312024 (PMC11841906; doi:10.1371/journal.pone.0312024)
Supplement: S1 Table — (PDF) [file pone.0312024.s003.pdf]

S1 Table. All studies identified in the literature search (n=504)

No	Literatures	Included/Excluded	Reason for Exclusion (if excluded)
1	Ferede AT, Dadi AF, Tariku A, Adane AA. Prevalence and determinants of active trachoma among preschool-aged children in Dembia District Northwest Ethiopia. *Infect Dis Poverty*. 2017;**6**(1):128. doi: 10.1186/s40249-017-0345-8.	Excluded	Published outside the eligible date range (2019–2024)
2	Tadesse B, Worku A, Kumie A, Yimer SA. Effect of water, sanitation and hygiene interventions on active trachoma in North and South Wollo zones of Amhara Region, Ethiopia: A Quasi-experimental study. PLoS neglected tropical diseases. 2017 Nov 10;11(11):e0006080.	Excluded	Inappropriate study design (non-observational) and Published outside the eligible date range (2019–2024)
3	Ferede AT, Dadi AF, Tariku A, Adane AA. Prevalence and determinants of active trachoma among preschool-aged children in Dembia District Northwest Ethiopia. *Infect Dis Poverty*. 2017;**6**(1):128. doi: 10.1186/s40249-017-0345-8.	Excluded	Duplicated
4	Getachew D, Woldekidan F, Ayele G, Bekele Y, Sleshi S, Tekalgn E, et al. High prevalence of active trachoma and associated factors among school-aged children in Southwest Ethiopia. PLOS Neglected Tropical Diseases. 2023;17(12):e0011846.	Included	
5	Ketema K, Tiruneh M, Woldeyohannes D, Muluye D. Active trachoma and associated risk factors among children in Baso Liben District of East Gojjam Ethiopia. *BMC Public Health.* 2012;**12**(1):1105. doi: 10.1186/1471-2458-12-1105.	Excluded	Published outside the eligible date range (2019–2024)
6	Emerson PM, Ngondi J, Biru E, Graves PM, Ejigsemahu Y, Gebre T, Endeshaw T, Genet A, Mosher AW, Zerihun M. Integrating an NTD with one	Excluded	Published outside the eligible date range (2019–2024)

	of “the big three”: combined malaria and trachoma survey in Amhara region of Ethiopia. PLoS Negl Trop Dis. 2008;2(3):e197		
7	Genet A, Dagnew Z, Melkie G, Keleb A, Motbainor A, Mebrat A, et al. Prevalence of active trachoma and its associated factors among 1–9 years of age children from model and non-model kebeles in Dangila district, northwest Ethiopia. Plos one. 2022;17(6):e0268441.	Included	
8	Zerihun N. Impact of trachoma among rural Ethiopian women. Ethiopian Journal of Health sciences. 1998 Jan 1;8(1):53-9.	Excluded	Excluded due to study population not meeting the inclusion criteria and Published outside the eligible date range (2019–2024)
9	Ferede AT, Dadi AF, Tariku A, Adane AA. Prevalence and determinants of active trachoma among preschool-aged children in Dembia District Northwest Ethiopia. *Infect Dis Poverty.* 2017;**6**(1):128. doi: 10.1186/s40249-017-0345-8.	Excluded	Duplicated
10	Anteneh ZA, Getu WY. Prevalence of active trachoma and associated risk factors among children in Gazegibela district of Wagehemra Zone, Amhara region, Ethiopia: community-based cross-sectional study. *Trop Dis Travel Med Vaccines.* 2016;**2**(1):5. doi: 10.1186/s40794-016-0022-0	Excluded	Published outside the eligible date range (2019–2024)
11	Emerson PM, Ngondi J, Biru E, Graves PM, Ejigsemahu Y, Gebre T, Endeshaw T, Genet A, Mosher AW, Zerihun M. Integrating an NTD with one of “the big three”: combined malaria and trachoma survey in Amhara region of Ethiopia. PLoS Negl Trop Dis. 2008;2(3):e197	Excluded	Published outside the eligible date range (2019–2024)
12	Aragie S, Wittberg DM, Tadesse W, Dagnew A, Hailu D, Chernet A, Melo JS, Aiemjoy K, Haile M, Zeru T, Tadesse Z. Water, sanitation, and hygiene for control of trachoma in Ethiopia (WUHA): a two-arm, parallel-	Excluded	Inappropriate study design (non-observational)

	group, cluster-randomised trial. The Lancet Global Health. 2022 Jan 1;10(1):e87-95		
13	Anteneh ZA, Getu WY. Prevalence of active trachoma and associated risk factors among children in Gazegibela district of Wagehemra Zone, Amhara region, Ethiopia: community-based cross-sectional study. *Trop Dis Travel Med Vaccines.* 2016;**2**(1):5. doi: 10.1186/s40794-016-0022-0	Excluded	Duplicated
14	Asmare ZA, Seifu BL, Mare KU, Asgedom YS, Kase BF, Shibeshi AH, et al. Prevalence and associated factors of active trachoma among 1–9 years of age children in Andabet district, northwest Ethiopia, 2023: A multi-level mixed-effect analysis. PLOS Neglected Tropical Diseases. 2023;17(8):e0011573.	Included	
15	Tuke D, Etu E, Shalemo E. Active trachoma prevalence and related variables among children in a pastoralist community in southern Ethiopia in 2021: a community-based cross-sectional study. The American Journal of Tropical Medicine and Hygiene. 2023;108(2):252.	Included	
16	Nigusie A, Berhe R, Gedefaw M. Prevalence and associated factors of active trachoma among childeren aged 1–9 years in rural communities of Gonji Kolella district, West Gojjam zone North West Ethiopia. *BMC Res Notes.* 2015;**8**(1):641. doi: 10.1186/s13104-015-1529-6.	Excluded	Published outside the eligible date range (2019–2024)
17	Shiferaw D, Moges HG. Risk factors for active trachoma among children aged 1-9 years in Maksegnit town, Gondar Zuria District Northwest Ethiopia. *Risk.* 2013;**2**(3):202–206	Excluded	Published outside the eligible date range (2019–2024)
18	Golovaty I, Jones L, Gelaye B, Tilahun M, Belete H, Kumie A, Berhane Y, Williams MA. Access to water source, latrine facilities and other risk factors of active	Excluded	Published outside the eligible date range (2019–2024)

	trachoma in Ankober Ethiopia. *PLoS One*. 2009;**4**(8):e6702. doi: 10.1371/journal.pone.0006702.		
19	Gedefaw M, Shiferaw A, Alamrew Z, Feleke A, Fentie T, Atnafu K: Current state of active trachoma among elementary school students in the context of ambitious national growth plan: The case of Ethiopia. Health (NY) 2013, 5(11):1768.	Excluded	Published outside the eligible date range (2019–2024)
20	Oswald WE, Stewart AE, Kramer MR, Endeshaw T, Zerihun M, Melak B, Sata E, Gessese D, Teferi T, Tadesse Z. Active trachoma and community use of sanitation Ethiopia. Bull World Health Organ. 2017;95(4):250	Excluded	Published outside the eligible date range (2019–2024)
21	Gedefaw M, Shiferaw A, Alamrew Z, Feleke A, Fentie T, Atnafu K: Current state of active trachoma among elementary school students in the context of ambitious national growth plan: The case of Ethiopia. Health (NY) 2013, 5(11):1768.	Excluded	Published outside the eligible date range (2019–2024)
22	Oswald WE, Stewart AE, Kramer MR, Endeshaw T, Zerihun M, Melak B, Sata E, Gessese D, Teferi T, Tadesse Z. Active trachoma and community use of sanitation Ethiopia. Bull World Health Organ. 2017;95(4):250	Excluded	Duplicated
23	Shiferaw D, Moges HG. Risk factors for active trachoma among children aged 1-9 years in Maksegnit town, Gondar Zuria District Northwest Ethiopia. *Risk*. 2013;**2**(3):202–206	Excluded	Duplicated
24	Golovaty I, Jones L, Gelaye B, Tilahun M, Belete H, Kumie A, Berhane Y, Williams MA. Access to water source, latrine facilities and other risk factors of active trachoma in Ankober Ethiopia. *PLoS One*. 2009;**4**(8):e6702. doi: 10.1371/journal.pone.0006702.	Excluded	Duplicated

25	Negash K, Macleod C, Adamu Y, Ahmed M, Ibrahim M, Ali M, Haileselassie T, Willis R, Chu BK, Dejene M. Prevalence of trachoma in the Afar Region of Ethiopia: results of seven population-based surveys from the Global Trachoma Mapping Project. Ophthalmic Epidemiol. 2018;25(sup1):3–10	Excluded	Published outside the eligible date range (2019–2024)
26	Adamu Y, Macleod C, Adamu L, Fikru W, Kidu B, Abashawl A, Dejene M, Chu BK, Flueckiger RM, Willis R, et al. Prevalence of Trachoma in Benishangul Gumuz Region, Ethiopia: Results of Seven Population-Based Surveys from the Global Trachoma Mapping Project. Ophthalmic Epidemiol. 2016;23(sup1):70–6	Excluded	Published outside the eligible date range (2019–2024)
27	Emerson PM, Ngondi J, Biru E, Graves PM, Ejigsemahu Y, Gebre T, Endeshaw T, Genet A, Mosher AW, Zerihun M. Integrating an NTD with one of "the big three": combined malaria and trachoma survey in Amhara region of Ethiopia. PLoS Negl Trop Dis. 2008;2(3):e197	Excluded	Duplicated
28	Alemayehu M, Koye DN, Tariku A, Yimam K. Prevalence of active trachoma and its associated factors among rural and urban children in Dera Woreda Northwest Ethiopia: a comparative cross-sectional study. BioMed Res Int. 2015;2015	Excluded	Published outside the eligible date range (2019–2024)
29	Abashawl A, Macleod C, Riang J, Mossisa F, Dejene M, Willis R, Flueckiger RM, Pavluck AL, Tadesse A, Adera TH, et al. Prevalence of Trachoma in Gambella Region, Ethiopia: Results of Three Population-Based Prevalence Surveys Conducted with the Global Trachoma Mapping Project. Ophthalmic Epidemiol. 2016;23(sup1):77–83.	Excluded	Published outside the eligible date range (2019–2024)
30	Nega A, Aklilu AR, Abdosh AT, Jelalu KB, Eskindr DZ. Prevalence and factors associated with trachoma	Excluded	Published outside the eligible date range (2019–2024)

	among primary school children in Harari region Eastern Ethiopia. Ophthalmol Res. 2017;7(3):1–9		
31	Alambo MM, Lake EA, Bitew Workie S, Wassie AY. Prevalence of active trachoma and associated factors in Areka Town, south Ethiopia, 2018. Interdisciplinary Perspectives on Infectious Diseases. 2020;2020.	Included	
32	Mekonnen J, Kassim J, Ahmed M, Gebeyehu N. Prevalence of active trachoma and associated factors among children 1–9 years old at Arsi Negele Town, West Arsi Zone, Oromia Regional State, Southern Ethiopia. Plos one. 2022;17(10):e0273808.	Included	
33	Shimelash A, Alemayehu M, Dagne H, Mihiretie G, Lamore Y, Tegegne E, et al. Prevalence of active trachoma and associated factors among school age children in Debre Tabor Town, Northwest Ethiopia, 2019: a community based cross-sectional study. Italian Journal of Pediatrics. 2022;48(1):1-9.	Included	
34	Belsti Y, Fekadu SA, Assem AS. Active trachoma prevalence and its associated factors among children aged 1-9 years in rural residents of Lare District, Southwest Ethiopia. International Journal of Ophthalmology. 2021;14(11):1756.	Included	
35	Yalew KN, Mekonnen MG, Jemaneh AA. Trachoma and its determinants in Mojo and Lume districts of Ethiopia. Pan Afr Med J. 2012;13(Suppl 1).	Excluded	Published outside the eligible date range (2019–2024)
36	Ejigu M, Kariuki MM, Ilako DR, Gelaw Y. Rapid trachoma assessment in kersa district, Southwest Ethiopia. Ethiop J Health Sci. 2013;23(1):1–9	Excluded	Published outside the eligible date range (2019–2024)
37	Emerson PM, Ngondi J, Biru E, Graves PM, Ejigsemahu Y, Gebre T, Endeshaw T, Genet A, Mosher AW, Zerihun M. Integrating an NTD with one	Excluded	Duplicated

	of “the big three”: combined malaria and trachoma survey in Amhara region of Ethiopia. PLoS Negl Trop Dis. 2008;2(3):e197		
38	Mehari ZA. Pattern of childhood ocular morbidity in rural eye hospital Central Ethiopia. BMC Ophthalmol. 2014;14(1):50.	Excluded	Published outside the eligible date range (2019–2024)
39	Ejigu M, Kariuki MM, Ilako DR, Gelaw Y. Rapid trachoma assessment in kersa district, Southwest Ethiopia. Ethiop J Health Sci. 2013;23(1):1–9	Excluded	Duplicated
40	Yalew KN, Mekonnen MG, Jemaneh AA. Trachoma and its determinants in Mojo and Lume districts of Ethiopia. Pan Afr Med J. 2012;13(Suppl 1).	Excluded	Duplicated
41	Gelaye B, Kumie A, Aboset N, Berhane Y, Williams MA. School-based intervention: evaluating the role of water, latrines and hygiene education on trachoma and intestinal parasitic infections in Ethiopia. Journal of water, sanitation and hygiene for development. 2014 Mar 1;4(1):120-30.	Excluded	Published outside the eligible date range (2019–2024)
42	Mehari ZA. Pattern of childhood ocular morbidity in rural eye hospital Central Ethiopia. BMC Ophthalmol. 2014;14(1):50	Excluded	Duplicated
43	Wondimu A, Bejiga A. Prevalence of trachomatous trichiasis in the community of Alaba District, Southern Ethiopia. East African medical journal. 2003;80(7):365-9.	Excluded	Excluded due to the outcome of interest not reported
44	Tabor Yimam A, Wassie GT, Alene GD. Postoperative trachomatous trichiasis and associated factors among adults who underwent trachomatous trichiasis surgery in Ambassel District, North-East Ethiopia. Plos one. 2024 May 28;19(5):e0304407.	Excluded	Excluded due to the outcome of interest not reported
45	Rajak SN, Habtamu E, Weiss HA, Bedri A, Gebre T, Bailey RL, Mabey DC, Khaw PT, Gilbert CE, Emerson PM, Burton MJ. The clinical phenotype of	Excluded	Excluded due to the outcome of interest not reported

	trachomatous trichiasis in Ethiopia: not all trichiasis is due to entropion. Investigative ophthalmology & visual science. 2011 Oct 1;52(11):7974-80		
46	Habte D, Gebre T, Zerihun M, Carter Center-Ethiopia, Regional Coordinator, Assefa Y. Determinants of uptake of surgical treatment for trachomatous trichiasis in North Ethiopia. Ophthalmic epidemiology. 2008 Jan 1;15(5):328-33.	Excluded	Excluded due to the outcome of interest not reported
47	Glagn Abdilwohab M, Hailemariam Abebo Z. High prevalence of clinically active trachoma and its associated risk factors among preschool-aged children in Arba Minch Health and demographic surveillance site, southern Ethiopia. Clinical Ophthalmology. 2020:3709-18.	Included	
48	Ayelgn K, Guadu T, Getachew A. Low prevalence of active trachoma and associated factors among children aged 1–9 years in rural communities of Metema District, Northwest Ethiopia: a community based cross-sectional study. Italian Journal of Pediatrics. 2021;47(1):1-8.	Included	
49	Kedir S, Lemnuro K, Yesse M, Abdella B, Muze M, Mustefa A, et al. Prevalence and Factors Associated with Active Trachoma among Children 1-9 years of Age in the Catchment Population of Tora Primary Hospital, Silte zone, Southern Ethiopia, 2020. The Open Ophthalmology Journal. 2021;15(1).	Included	
50	Shafi Abdurahman O, Last A, Macleod D, Habtamu E, Versteeg B, Dumessa G, et al. Trachoma risk factors in Oromia Region, Ethiopia. PLoS Neglected Tropical Diseases. 2023;17(11):e0011679.	Included	

51	Mehari ZA. Pattern of childhood ocular morbidity in rural eye hospital Central Ethiopia. BMC Ophthalmol. 2014;14(1):50.	Excluded	Duplicated
52	Habtamu E, Wondie T, Aweke S, Tadesse Z, Zerihun M, Gashaw B, Wondimagegn GS, Mengistie HD, Rajak SN, Callahan K, Weiss HA. Predictors of trachomatous trichiasis surgery outcome. Ophthalmology. 2017 Aug 1;124(8):1143-55.	Excluded	Excluded due to the outcome of interest not reported
53	Churko C, Asfaw MA, Zerdo Z. Exploring barriers for trachomatous trichiasis surgery implementation in gamo zone, Southern Ethiopia. PLoS Neglected Tropical Diseases. 2021 Sep 15;15(9):e0009780.	Excluded	Excluded due to the outcome of interest not reported
54	Assefa Y, Habte D, Yigzaw T, Mekonnen A, Gebrie T, Dubale T, Zerihun M. Trichiasis ercurrence in Nother Ethiopia: a one year prospecitve study of Trachomatous Trichiasis surgery done by integrated eye care workers. Ethiopian Journal of Health Development. 2008 Aug 27;22(1):8-13.	Excluded	Excluded due to the outcome of interest not reported
55	Churko C, Asfaw MA, Zerdo Z. Knowledge and attitude of community towards trachoma and trichiasis in Arba Minch Zuria district, Gamo Zone, Southern Ethiopia, 2019.	Excluded	Excluded due to the outcome of interest not reported
56	Adafrie Y, Redae G, Zenebe D, Adhena G. Uptake of trachoma trichiasis surgery and associated factors among trichiasis-diagnosed clients in southern Tigray, Ethiopia. Clinical Ophthalmology. 2021 May 10:1939-48	Excluded	Excluded due to the outcome of interest not reported
57	Rajak SN, Habtamu E, Weiss HA, Bedri A, Gebre T, Genet A, Khaw PT, Bailey RL, Mabey DC, Gilbert CE, Emerson PM. Epilation for trachomatous trichiasis and the risk of corneal opacification. Ophthalmology. 2012 Jan 1;119(1):84-9.	Excluded	Duplicated

58	Meron Y, Mandefro S, Haymanot M. Active Trachoma and Associated Factors among Children (Aged 1-9 years) in Haramaya District, Eastern Ethiopia. East African Journal of Health and Biomedical Sciences. 2022;6(1):1-10.	Included	
59	Delelegn D, Tolcha A, Beyene H, Tsegaye B. Status of active trachoma infection among school children who live in villages of open field defecation: a comparative cross-sectional study. BMC Public Health. 2021;21:1-10.	Included	
60	Kassaw MW, Abebe AM, Tegegne KD, Getu MA, Bihonegn WT. Prevalence and associations of active trachoma among rural preschool children in Wadla district, northern Ethiopia. BMC ophthalmology. 2020;20:1-10.	Included	
61	Reda G, Yemane D, Gebreyesus A. Prevalence and associated factors of active trachoma among 1–9 years old children in Deguatemben, Tigray, Ethiopia, 2018: community cross-sectional study. BMC ophthalmology. 2020;20(1):1-9.	Included	
62	Seyum D, Fetene N, Kifle T, Negash H, Kabeto T, Gebre M, et al. Prevalence of trachoma from 66 impact surveys in 52 woredas of Southern Nations, Nationalities and Peoples' and Sidama Regions of Ethiopia, 2017–2019. Ophthalmic epidemiology. 2023;30(6):637-46.	Included	
63	Miecha H, Dejene M, Adugna D, Kebede A, Yadeta D, Alemayehu A, et al. Prevalence of Trachoma in Pre-validation Surveillance Surveys in 11 Evaluation Units (Covering 12 Districts) in Oromia Regional	Included	

	State, Ethiopia: Results from 2018− 2020. Ophthalmic epidemiology. 2023;30(6):655-62.		
64	Nash SD, Astale T, Nute AW, Bethea D, Chernet A, Sata E, et al. Population-based prevalence of Chlamydia trachomatis infection and antibodies in four districts with varying levels of trachoma endemicity in Amhara, Ethiopia. The American journal of tropical medicine and hygiene. 2021;104(1):207	Included	
65	Rajak SN, Habtamu E, Weiss HA, Bedri A, Gebre T, Genet A, Khaw PT, Bailey RL, Mabey DC, Gilbert CE, Emerson PM. Epilation for trachomatous trichiasis and the risk of corneal opacification. Ophthalmology. 2012 Jan 1;119(1):84-9.	Excluded	Duplicated
66	Adafrie Y, Redae G, Zenebe D, Adhena G. Uptake of trachoma trichiasis surgery and associated factors among trichiasis-diagnosed clients in southern Tigray, Ethiopia. Clinical Ophthalmology. 2021 May 10:1939-48.	Excluded	Duplicated
67	Churko C, Asfaw MA, Zerdo Z. Knowledge and attitude of community towards trachoma and trichiasis in Arba Minch Zuria district, Gamo Zone, Southern Ethiopia, 2019	Excluded	Duplicated
68	Assefa Y, Habte D, Yigzaw T, Mekonnen A, Gebrie T, Dubale T, Zerihun M. Trichiasis ercurrence in Nother Ethiopia: a one year prospecitve study of Trachomatous Trichiasis surgery done by integrated eye care workers. Ethiopian Journal of Health Development. 2008 Aug 27;22(1):8-13.	Excluded	Duplicated
69	Adafrie Y, Redae G, Zenebe D, Adhena G. Uptake of trachoma trichiasis surgery and associated factors among trichiasis-diagnosed clients in southern Tigray,	Excluded	Duplicated

	Ethiopia. Clinical Ophthalmology. 2021 May 10:1939-48		
70	Rajak SN, Habtamu E, Weiss HA, Bedri A, Gebre T, Genet A, Khaw PT, Bailey RL, Mabey DC, Gilbert CE, Emerson PM. Epilation for trachomatous trichiasis and the risk of corneal opacification. Ophthalmology. 2012 Jan 1;119(1):84-9.	Excluded	Duplicated
71	Meshesha TD, Senbete GH, Bogale GG. Determinants for not utilizing trachomatous trichiasis surgery among trachomatous trichiasis patients in Mehalsayint District, North-East Ethiopia. PLoS neglected tropical diseases. 2018 Jul 18;12(7):e0006669.	Excluded	Excluded due to the outcome of interest not reported
72	Burn H, Aweke S, Wondie T, Habtamu E, Deribe K, Rajak S, Bremner S, Davey G. Podoconiosis, trachomatous trichiasis and cataract in northern Ethiopia: A comparative cross sectional study. PLoS neglected tropical diseases. 2017 Feb 10;11(2):e0005388.	Excluded	Excluded due to the outcome of interest not reported
73	Melese M, West ES, Alemayehu W, Munoz B, Worku A, Gaydos CA, West SK. Characteristics of trichiasis patients presenting for surgery in rural Ethiopia. British Journal of Ophthalmology. 2005 Sep 1;89(9):1084-8.	Excluded	Excluded due to the outcome of interest not reported
74	Mehari ZA. Pattern of childhood ocular morbidity in rural eye hospital, Central Ethiopia. BMC ophthalmology. 2014 Dec;14:1-6	Excluded	Excluded due to the outcome of interest not reported
75	Habtamu E, Weiss HA, Bedri A, Gebre T, Bailey RL, Mabey DC, Khaw PT, Gilbert CE, Emerson PM, Burton MJ. The clinical phenotype of trachomatous trichiasis in Ethiopia: not all trichiasis is due to entropion. Investigative ophthalmology & visual science. 2011 Oct 1;52(11):7974-80	Excluded	Excluded due to the outcome of interest not reported

76	Rajak SN, Habtamu E, Weiss HA, Kello AB, Gebre T, Genet A, Bailey RL, Mabey DC, Khaw PT, Gilbert CE, Emerson PM. Absorbable versus silk sutures for surgical treatment of trachomatous trichiasis in Ethiopia: a randomised controlled trial. PLoS medicine. 2011 Dec 13;8(12):e1001137.	Excluded	Duplicated
77	Melese K. *Determinants of Corrective Upper Eye Lid Surgery Refusals Among Trachomatous Trichiasis Patients in Mecha Woreda, West Gojjam Zone, Ethiopia, 2018 GC* (Doctoral dissertation).	Excluded	Excluded due to the outcome of interest not reported
78	Wang EY, Kong X, Wolle M, Gasquet N, Ssekasanvu J, Mariotti SP, Bourne R, Taylor H, Resnikoff S, West S. Global trends in blindness and vision impairment resulting from corneal opacity 1984–2020: A meta-analysis. Ophthalmology. 2023 Aug 1;130(8):863-71.	Excluded	Excluded due to the outcome of interest not reported
	Melese K. *Determinants of Corrective Upper Eye Lid Surgery Refusals Among Trachomatous Trichiasis Patients in Mecha Woreda, West Gojjam Zone, Ethiopia, 2018 GC* (Doctoral dissertation).	Excluded	Duplicated
79	Rajak SN, Habtamu E, Weiss HA, Kello AB, Gebre T, Genet A, Bailey RL, Mabey DC, Khaw PT, Gilbert CE, Emerson PM. Surgery versus epilation for the treatment of minor trichiasis in Ethiopia: a randomised controlled noninferiority trial. PLoS medicine. 2011 Dec 13;8(12):e1001136	Excluded	Duplicated
80	Burn H, Aweke S, Wondie T, Habtamu E, Deribe K, Rajak S, Bremner S, Davey G. Podoconiosis, trachomatous trichiasis and cataract in northern Ethiopia: A comparative cross sectional study. PLoS neglected tropical diseases. 2017 Feb 10;11(2):e0005388.	Excluded	Duplicated
81	Melese K. *Determinants of Corrective Upper Eye Lid Surgery Refusals Among Trachomatous Trichiasis*	Excluded	Duplicated

	Patients in Mecha Woreda, West Gojjam Zone, Ethiopia, 2018 GC (Doctoral dissertation).		
82	Bejiga A, Alemayehu W. Prevalence of trachoma and its determinants in Dalocha District Central. Ethiopia Ophthalmic Epidemiol. 2001;8(2–3):119–25	Excluded	Published outside the eligible date range (2019–2024)
83	Nigusie A, Berhe R, Gedefaw M. Prevalence and associated factors of active trachoma among childeren aged 1–9 years in rural communities of Gonji Kolella district, West Gojjam zone North West Ethiopia. *BMC Res Notes.* 2015;**8**(1):641. doi: 10.1186/s13104-015-1529-6.	Excluded	Published outside the eligible date range (2019–2024)
84	Shiferaw D, Moges HG. Risk factors for active trachoma among children aged 1-9 years in Maksegnit town, Gondar Zuria District Northwest Ethiopia. *Risk.* 2013;**2**(3):202–206	Excluded	Published outside the eligible date range (2019–2024)
85	Abashawl A, Macleod C, Riang J, Mossisa F, Dejene M, Willis R, Flueckiger RM, Pavluck AL, Tadesse A, Adera TH, et al. Prevalence of Trachoma in Gambella Region, Ethiopia: Results of Three Population-Based Prevalence Surveys Conducted with the Global Trachoma Mapping Project. Ophthalmic Epidemiol. 2016;23(sup1):77–83	Excluded	Published outside the eligible date range (2019–2024)
86	Berhane Y, Worku A, Bejiga A. National survey on blindness, low vision and trachoma in Ethiopia. Fed Minist Health Ethiop. 2006	Excluded	Published outside the eligible date range (2019–2024)
87	Wondie T, Aweke S, Tadesse Z, Zerihun M, Zewdie Z, Callahan K, Emerson PM, Kuper H, Bailey RL. Trachoma and relative poverty: a casecontrol study. PLoS Negl Trop Dis. 2015;9(11):e0004228	Excluded	Published outside the eligible date range (2019–2024)
88	Gebre T, Shargie EB, Graves PM, Ejigsemahu Y, Teferi T, Genet A, Mosher AW, Endeshaw T, Zerihun M. Risk factors for active trachoma in children and trichiasis in adults: a household survey in Amhara	Excluded	Published outside the eligible date range (2019–2024)

	regional state, Ethiopia. Trans R Soc Trop Med Hyg. 2008;102(5):432–8.		
89	Melese M, Fredlander E, Worku A, Courtright P. Active trachoma in children in central Ethiopia: association with altitude. Transactions of the Royal Society of Tropical Medicine and Hygiene. 2005 Nov 1;99(11):840-3.	Excluded	Published outside the eligible date range (2019–2024)
90	Mekonnen MG, Jemaneh AA. Trachoma and its determinants in Mojo and Lume districts of Ethiopia. The Pan African Medical Journal. 2012;13(Suppl 1).	Excluded	Published outside the eligible date range (2019–2024)
91	Stocks ME, Ogden S, Haddad D, Addiss DG, McGuire C, Freeman MC. Effect of water, sanitation, and hygiene on the prevention of trachoma: a systematic review and meta-analysis. PLoS medicine. 2014 Feb 25;11(2):e1001605.	Excluded	Excluded due to the outcome of interest not reported
92	Prüss A, Mariotti SP. Preventing trachoma through environmental sanitation: a review of the evidence base. Bulletin of the World Health Organization. 2000;78(2):267-73.	Excluded	Excluded due to the outcome of interest not reported
93	Alemayehu W, Melese M, Fredlander E, Worku A, Courtright P. Active trachoma in children in Central Ethiopia: association with altitude. Trans R Soc Trop Med Hyg. 2005;99(11):840–3.	Excluded	Duplicated
94	Ejere HO, Alhassan MB, Rabiu M. Face washing promotion for preventing active trachoma. Cochrane Database of Systematic Reviews. 2015(2).	Excluded	Excluded due to the outcome of interest not reported
95	Stocks ME, Ogden S, Haddad D, Addiss DG, McGuire C, Freeman MC. Effect of water, sanitation, and hygiene on the prevention of trachoma: a systematic review and meta-analysis. PLoS medicine. 2014 Feb 25;11(2):e1001605.	Excluded	Duplicated
96	Prüss A, Mariotti SP. Preventing trachoma through environmental sanitation: a review of the evidence	Excluded	Duplicated

	base. Bulletin of the World Health Organization. 2000;78(2):267-73.		
97	Melese M, Fredlander E, Worku A, Courtright P. Active trachoma in children in central Ethiopia: association with altitude. Transactions of the Royal Society of Tropical Medicine and Hygiene. 2005 Nov 1;99(11):840-3.	Excluded	Duplicated
98	West SK. Blinding trachoma: prevention with the safe strategy. American Journal of Tropical Medicine and Hygiene. 2003 Nov 1;69(5):18-23.	Excluded	Excluded due to the outcome of interest not reported
99	Bailey R, Lietman T. The SAFE strategy for the elimination of trachoma by 2020: will it work?. Bulletin of the World Health Organization. 2001;79:233-6.	Excluded	Excluded due to the outcome of interest not reported
100	Chen X, Munoz B, Mkocha H, Wolle MA, K. West S. Children as messengers of health knowledge? Impact of health promotion and water infrastructure in schools on facial cleanliness and trachoma in the community. PLoS neglected tropical diseases. 2021 Feb 1;15(2):e0009119	Excluded	Excluded due to the outcome of interest not reported
101	Karimurio J, Ilako DR, Adala HS, Gichangi M, Kilima P. Prevalence of trachoma in six districts of Kenya. East African medical journal. 2006 Sep 12;83(4):63-8.	Excluded	Conducted outside the eligible geographic area (Ethiopia)
102	Karimurio J, Ilako F, Gichangi M. Prevalence of active and potentially blinding trachoma in Laikipia district, Kenya.	Excluded	Conducted outside the eligible geographic area (Ethiopia)
103	Nasieku L, Mutai J, Muthami L, Karanja S. Determinants of active trachoma among children aged 1-9 years in Ol Donyo Nyokie location, Kajiado County, Kenya. African Journal of Health Sciences. 2017;30(2):77-86.	Excluded	Conducted outside the eligible geographic area (Ethiopia)

104	Kamau JW. *Prevalence, Risk Factors and Trachoma Causing Species Circulating in East Pokot, Baringo County, Kenya* (Doctoral dissertation).	Excluded	Conducted outside the eligible geographic area (Ethiopia)
105	Nyamwaro CM. Prevalence, and Risk Factors for Trachoma Infection Among Children Aged 1–9 Years Old in Oldonyonyokie Location, Magadi Division, Kajiado County, Kenya.	Excluded	Conducted outside the eligible geographic area (Ethiopia)
106	Renneker KK, Abdala M, Addy J, Al-Khatib T, Amer K, Badiane MD, Batcho W, Bella L, Bougouma C, Bucumi V, Chisenga T. Global progress toward the elimination of active trachoma: an analysis of 38 countries. The Lancet Global Health. 2022 Apr 1;10(4):e491-500.	Excluded	Conducted outside the eligible geographic area (Ethiopia)
107	Njomo DW, Karimurio J, Odhiambo GO, Mukuria M, Wanyama EB, Rono HK, Gichangi M. Knowledge, practices and perceptions of trachoma and its control among communities of Narok County, Kenya. Tropical diseases, travel medicine and vaccines. 2016 Dec;2:1-0.	Excluded	Conducted outside the eligible geographic area (Ethiopia)
108	Nyamwaro CM. Prevalence, and Risk Factors for Trachoma Infection Among Children Aged 1–9 Years Old in Oldonyonyokie Location, Magadi Division, Kajiado County, Kenya	Excluded	Conducted outside the eligible geographic area (Ethiopia)
109	Karimurio J, Ilako DR, Adala HS, Gichangi M, Kilima P. Prevalence of trachoma in six districts of Kenya. East African medical journal. 2006 Sep 12;83(4):63-8.	Excluded	Conducted outside the eligible geographic area (Ethiopia)
110	Ng'etich AS, Owino C, Juma A. Utilization of trachoma eye care services in central division of Kajiado County, Kenya. International Research Journal of Public and Environmental Health. 2016 Mar.	Excluded	Conducted outside the eligible geographic area (Ethiopia)

111	Karimurio J, Ilako DR, Adala HS, Gichangi M, Kilima P. Prevalence of trachoma in six districts of Kenya. East African medical journal. 2006 Sep 12;83(4):63-8	Excluded	Duplicated
112	Owino C, Juma A. Utilization of trachoma eye care services in central division of Kajiado County, Kenya. International Research Journal of Public and Environmental Health. 2016 Mar.	Excluded	Conducted outside the eligible geographic area (Ethiopia)
113	Ngondi J, Matthews F, Reacher M, Onsarigo A, Matende I, Baba S, Brayne C, Zingeser J, Emerson P. Prevalence of risk factors and severity of active trachoma in southern Sudan: an ordinal analysis. American Journal of Tropical Medicine and Hygiene. 2007 Jul 1;77(1):126-32.	Excluded	Conducted outside the eligible geographic area (Ethiopia)
114	Alemayehu W, Melese M, Fredlander E, Worku A, Courtright P. Active trachoma in children in central Ethiopia: association with altitude. Transactions of the Royal Society of Tropical Medicine and Hygiene. 2005 Nov 1;99(11):840-3.	Excluded	Duplicated
115	Ngondi J, Matthews F, Reacher M, Onsarigo A, Matende I, Baba S, Brayne C, Zingeser J, Emerson P. Prevalence of risk factors and severity of active trachoma in southern Sudan: an ordinal analysis. American Journal of Tropical Medicine and Hygiene. 2007 Jul 1;77(1):126-32.	Excluded	Duplicated
116	King JD, Ngondi J, Gatpan G, Lopidia B, Becknell S, Emerson PM. The burden of trachoma in ayod county of southern Sudan. PLoS Neglected Tropical Diseases. 2008 Sep 24;2(9):e299.	Excluded	Conducted outside the eligible geographic area (Ethiopia)
117	Yalew KN, Mekonnen MG, Jemaneh AA. Trachoma and its determinants in Mojo and Lume districts of Ethiopia. The Pan African Medical Journal. 2012;13(Suppl 1).	Excluded	Duplicated
		Excluded	

118	King JD, Ngondi J, Gatpan G, Lopidia B, Becknell S, Emerson PM. The burden of trachoma in ayod county of southern Sudan. PLoS Neglected Tropical Diseases. 2008 Sep 24;2(9):e299.	Excluded	Conducted outside the eligible geographic area (Ethiopia)
119	Ngondi J, Onsarigo A, Adamu L, Matende I, Baba S, Reacher M, Emerson P, Zingeser J. The epidemiology of trachoma in Eastern Equatoria and Upper Nile States, southern Sudan. Bulletin of the World Health Organization. 2005;83:904-12.	Excluded	Conducted outside the eligible geographic area (Ethiopia)
120	Hassan A, Ngondi JM, King JD, Elshafie BE, Al Ginaid G, Elsanousi M, Abdalla Z, Aziz N, Sankara D, Simms V, Cromwell EA. The prevalence of blinding trachoma in northern states of Sudan. PLoS neglected tropical diseases. 2011 May 31;5(5):e1027.	Excluded	Conducted outside the eligible geographic area (Ethiopia)
121	Alemayehu W, Melese M, Fredlander E, Worku A, Courtright P. Active trachoma in children in central Ethiopia: association with altitude. Transactions of the Royal Society of Tropical Medicine and Hygiene. 2005 Nov 1;99(11):840-3	Excluded	Duplicated
122	Zerihun N. Trachoma in Jimma zone, south western Ethiopia. Tropical Medicine & International Health. 1997 Dec;2(12):1115-21.	Excluded	Duplicated
123	Yalew KN, Mekonnen MG, Jemaneh AA. Trachoma and its determinants in Mojo and Lume districts of Ethiopia. The Pan African Medical Journal. 2012;13(Suppl 1).	Excluded	Duplicated
124	Zerihun N, Mabey D. Blindness and low vision in Jimma Zone, Ethopia: results of a population-based survey. Ophthalmic epidemiology. 1997 Jan 1;4(1):19-26.	Excluded	Excluded after title and abstract screening
125	Zerihun N. Trachoma in Jimma zone, south western Ethiopia. Tropical Medicine & International Health. 1997 Dec;2(12):1115-21.	Excluded	Duplicated

126	Melese M, Alemayehu W, Bayu S, Girma T, Hailesellasie T, Khandekar R, Worku A, Courtright P. Low vision and blindness in adults in Gurage Zone, central Ethiopia. British Journal of Ophthalmology. 2003 Jun 1;87(6):677-80.	Excluded	Excluded after title and abstract screening
127	Ngondi J, Onsarigo A, Adamu L, Matende I, Baba S, Reacher M, Emerson P, Zingeser J. The epidemiology of trachoma in Eastern Equatoria and Upper Nile States, southern Sudan. Bulletin of the World Health Organization. 2005;83:904-12.	Excluded	Duplicated
128	Cherinet FM, Tekalign SY, Anbesse DH, Bizuneh ZY. Prevalence and associated factors of low vision and blindness among patients attending St. Paul's Hospital Millennium Medical College, Addis Ababa, Ethiopia. BMC ophthalmology. 2018 Dec;18:1-6	Excluded	Excluded after title and abstract screening
129	Ngondi J, Onsarigo A, Adamu L, Matende I, Baba S, Reacher M, Emerson P, Zingeser J. The epidemiology of trachoma in Eastern Equatoria and Upper Nile States, southern Sudan. Bulletin of the World Health Organization. 2005;83:904-12.	Excluded	Duplicated
130	Cherinet FM, Tekalign SY, Anbesse DH, Bizuneh ZY. Prevalence and associated factors of low vision and blindness among patients attending St. Paul's Hospital Millennium Medical College, Addis Ababa, Ethiopia. BMC ophthalmology. 2018 Dec;18:1-6.	Excluded	Excluded after title and abstract screening
131	Zerihun N. Trachoma in Jimma zone, south western Ethiopia. Tropical Medicine & International Health. 1997 Dec;2(12):1115-21.	Excluded	Duplicated
132	Wale MZ, Abebe Y, Adamu Y, Zelalem A. Prevalence of color blindness among school children in three primary schools of Gish–Abay town district, Amhara regional state, north-west Ethiopia. BMC ophthalmology. 2018 Dec;18:1-6	Excluded	Excluded after title and abstract screening

134	Ngondi J, Onsarigo A, Adamu L, Matende I, Baba S, Reacher M, Emerson P, Zingeser J. The epidemiology of trachoma in Eastern Equatoria and Upper Nile States, southern Sudan. Bulletin of the World Health Organization. 2005;83:904-12.	Excluded	Duplicated
135	Stocks ME, Ogden S, Haddad D, Addiss DG, McGuire C, Freeman MC. Effect of water, sanitation, and hygiene on the prevention of trachoma: a systematic review and meta-analysis. PLoS medicine. 2014 Feb 25;11(2):e1001605	Excluded	Excluded after title and abstract screening
136	Sullivan KM, Harding-Esch EM, Keil AP, Freeman MC, Batcho WE, Bio Issifou AA, Bucumi V, Bella AL, Epee E, Bobo Barkesa S, Seife Gebretsadik F. Exploring water, sanitation, and hygiene coverage targets for reaching and sustaining trachoma elimination: G-computation analysis. PLoS neglected tropical diseases. 2023 Feb 13;17(2):e0011103	Excluded	Excluded after title and abstract screening
137	Prüss A, Mariotti SP. Preventing trachoma through environmental sanitation: a review of the evidence base. Bulletin of the World Health Organization. 2000;78(2):267-73.	Excluded	Excluded after title and abstract screening
138	Zerihun N. Trachoma in Jimma zone, south western Ethiopia. Tropical Medicine & International Health. 1997 Dec;2(12):1115-21.	Excluded	Duplicated
139	Esrey SA, Potash JB, Roberts L, Shiff C. Effects of improved water supply and sanitation on ascariasis, diarrhoea, dracunculiasis, hookworm infection, schistosomiasis, and trachoma. Bulletin of the World Health organization. 1991;69(5):609	Excluded	Excluded after title and abstract screening
140	Zerihun N. Trachoma in Jimma zone, south western Ethiopia. Tropical Medicine & International Health. 1997 Dec;2(12):1115-21.	Excluded	Duplicated

141	Oswald WE, Stewart AE, Kramer MR, Endeshaw T, Zerihun M, Melak B, Sata E, Gessese D, Teferi T, Tadesse Z, Guadie B. Active trachoma and community use of sanitation, Ethiopia. Bulletin of the World Health Organization. 2017 Apr 4;95(4):250	Excluded	Excluded after title and abstract screening
142	Ngondi J, Onsarigo A, Adamu L, Matende I, Baba S, Reacher M, Emerson P, Zingeser J. The epidemiology of trachoma in Eastern Equatoria and Upper Nile States, southern Sudan. Bulletin of the World Health Organization. 2005;83:904-12.	Excluded	Duplicated
143	Tadesse B, Worku A, Kumie A, Yimer SA. Effect of water, sanitation and hygiene interventions on active trachoma in North and South Wollo zones of Amhara Region, Ethiopia: A Quasi-experimental study. PLoS neglected tropical diseases. 2017 Nov 10;11(11):e0006080.	Excluded	Excluded after title and abstract screening
144	Zerihun N. Trachoma in Jimma zone, south western Ethiopia. Tropical Medicine & International Health. 1997 Dec;2(12):1115-21.	Excluded	Duplicated
145	Ferede AT, Dadi AF, Tariku A, Adane AA. Prevalence and determinants of active trachoma among preschool-aged children in Dembia District Northwest Ethiopia. *Infect Dis Poverty*. 2017;**6**(1):128. doi: 10.1186/s40249-017-0345-8.	Excluded	Excluded after title and abstract screening
146	Travers A, Strasser S, Palmer SL, Stauber C. The added value of water, sanitation, and hygiene interventions to mass drug administration for reducing the prevalence of trachoma: a systematic review examining. Journal of Environmental and Public Health. 2013;2013(1):682093.	Excluded	Excluded after title and abstract screening
147	Abebe TA, Tucho GT. The impact of access to water supply and sanitation on the prevalence of active trachoma in Ethiopia: A systematic review and meta-	Excluded	Excluded after title and abstract screening

	analysis. PLoS Neglected Tropical Diseases. 2021 Sep 9;15(9):e0009644.		
148	Ketema K, Tiruneh M, Woldeyohannes D, Muluye D. Active trachoma and associated risk factors among children in Baso Liben District of East Gojjam Ethiopia. *BMC Public Health.* 2012;**12**(1):1105. doi: 10.1186/1471-2458-12-1105	Excluded	Duplicated
149	Ntozini R. Trachoma control using water, sanitation, and hygiene. The Lancet Global Health. 2022 Jan 1;10(1):e10-1.	Excluded	Excluded after title and abstract screening
150	Emerson PM, Ngondi J, Biru E, Graves PM, Ejigsemahu Y, Gebre T, Endeshaw T, Genet A, Mosher AW, Zerihun M. Integrating an NTD with one of "the big three": combined malaria and trachoma survey in Amhara region of Ethiopia. PLoS Negl Trop Dis. 2008;2(3):e197	Excluded	Duplicated
151	Aragie S, Lietman TM, Keenan JD. Trachoma control using water, sanitation, and hygiene–Authors' reply. The Lancet Global Health. 2022 Apr 1;10(4):e480.	Excluded	Excluded after title and abstract screening
152	Delea MG, Solomon H, Solomon AW, Freeman MC. Interventions to maximize facial cleanliness and achieve environmental improvement for trachoma elimination: a review of the grey literature. PLoS neglected tropical diseases. 2018 Jan 25;12(1):e0006178.	Excluded	Excluded after title and abstract screening
153	Anteneh ZA, Getu WY. Prevalence of active trachoma and associated risk factors among children in Gazegibela district of Wagehemra Zone, Amhara region, Ethiopia: community-based cross-sectional study. *Trop Dis Travel Med Vaccines.* 2016;**2**(1):5. doi: 10.1186/s40794-016-0022-0	Excluded	Duplicated
154	Mpyet C, Tagoh S, Boisson S, Willis R, Muhammad N, Bakhtiari A, Adamu MD, Pavluck AL, Umar MM,	Excluded	Excluded after title and abstract screening

	Alada J, Isiyaku S. Prevalence of trachoma and access to water and sanitation in Benue State, Nigeria: results of 23 population-based prevalence surveys. Ophthalmic epidemiology. 2018 Dec 28;25(sup1):79-85.		
155	Tadesse B, Worku A, Kumie A, Yimer SA. The burden of and risk factors for active trachoma in the north and south Wollo zones of Amhara region, Ethiopia: a cross-sectional study. *Infect Dis Poverty*. 2017;**6**(1):143. doi: 10.1186/s40249-017-0358-3.	Excluded	Duplicated
156	Dyer CE, Kalua K, Chisambi AB, Wand H, McManus H, Liu B, Kaldor JM, Vaz Nery S. Water, Sanitation, and Hygiene (WASH) Factors Influencing the Effectiveness of Mass Drug Administration to Eliminate Trachoma as a Public Health Problem in Malawi. Ophthalmic Epidemiology. 2024 Mar 3;31(2):127-33.	Excluded	Excluded after title and abstract screening
157	Aragie S, Wittberg DM, Tadesse W, Dagnew A, Hailu D, Chernet A, Melo JS, Aiemjoy K, Haile M, Zeru T, Tadesse Z. Water, sanitation, and hygiene for control of trachoma in Ethiopia (WUHA): a two-arm, parallel-group, cluster-randomised trial. The Lancet Global Health. 2022 Jan 1;10(1):e87-95.	Excluded	Duplicated
158	Chikwanda M, Munyinda N, Mwale C, Mbanefo P, Banda TC, Mubita P. An association between water, sanitation, and hygiene (WASH) and prevalence of trachoma in Monze district of Southern Province, Zambia. Journal of Water, Sanitation and Hygiene for Development. 2021 May 1;11(3):453-60.	Excluded	Excluded after title and abstract screening
159	Ngondi J, Matthews F, Reacher M, Baba S, Brayne C, Emerson P. Associations between active trachoma and community intervention with antibiotics, facial	Excluded	Excluded after title and abstract screening

	cleanliness, and environmental improvement (A, F, E). PLoS neglected tropical diseases. 2008 Apr 30;2(4):e229.		
160	Tadesse B, Worku A, Kumie A, Yimer SA. The burden of and risk factors for active trachoma in the north and south Wollo zones of Amhara region, Ethiopia: a cross-sectional study. *Infect Dis Poverty*. 2017;**6**(1):143. doi: 10.1186/s40249-017-0358-3.	Excluded	Duplicated
161	Tadesse Z, Callahan K, Emerson PM, Aragie S, Gebeyehu W, Freeman MC, Cevallos V, Melo J, Wittberg DM, Porco TC, Lietman TM. Sanitation, Water, and Instruction in Face-washing for Trachoma I.	Excluded	Excluded after title and abstract screening
162	Reacher M, Baba S, Brayne C, Emerson P. Associations between active trachoma and community intervention with antibiotics, facial cleanliness, and environmental improvement (A, F, E). PLoS neglected tropical diseases. 2008 Apr 30;2(4):e229.	Excluded	Excluded after title and abstract screening
163	Mohammadpour M, Abrishami M, Masoumi A, Hashemi H. Trachoma: Past, present and future. Journal of current ophthalmology. 2016 Dec 1;28(4):165-9	Excluded	Excluded after title and abstract screening
164	Aragie S, Wittberg DM, Tadesse W, Dagnew A, Hailu D, Chernet A, Melo JS, Aiemjoy K, Haile M, Zeru T, Tadesse Z. Water, sanitation, and hygiene for control of trachoma in Ethiopia (WUHA): a two-arm, parallel-group, cluster-randomised trial. The Lancet Global Health. 2022 Jan 1;10(1):e87-95.	Excluded	Duplicated
165	Abebe TA, Tucho GT. The impact of access to water supply and sanitation on the prevalence of active trachoma in Ethiopia: A systematic review and meta-	Excluded	Excluded after title and abstract screening

	analysis. PLoS Neglected Tropical Diseases. 2021 Sep 9;15(9):e0009644.		
166	Ntozini R. Trachoma control using water, sanitation, and hygiene. The Lancet Global Health. 2022 Jan 1;10(1):e10-1.	Excluded	Excluded after title and abstract screening
167	Sarr B, Sissoko M, Fall M, Nizigama L, Cohn D, Willis R, Fuller B, O'Neil M, Solomon AW, Global Trachoma Mapping Project. Prevalence of trachoma in Senegal: Results of baseline surveys in 17 districts. Ophthalmic epidemiology. 2018 Dec 28;25(sup1):41-52.	Excluded	Excluded after title and abstract screening
168	Lavett DK, Lansingh VC, Carter MJ, Eckert KA, Silva JC. Will the SAFE strategy be sufficient to eliminate trachoma by 2020? Puzzlements and possible solutions. The Scientific World Journal. 2013;2013(1):648106.	Excluded	Excluded after title and abstract screening
169	Cumberland P, Edwards T, Hailu G, Harding-Esch E, Andreasen A, Mabey D, Todd J. The impact of community level treatment and preventative interventions on trachoma prevalence in rural Ethiopia. International journal of epidemiology. 2008 Jun 1;37(3):549-58.	Excluded	Excluded after title and abstract screening
170	Cumberland P, Edwards T, Hailu G, Harding-Esch E, Andreasen A, Mabey D, Todd J. The impact of community level treatment and preventative interventions on trachoma prevalence in rural Ethiopia. International journal of epidemiology. 2008 Jun 1;37(3):549-58.	Excluded	Excluded after title and abstract screening
171	Lansingh VC, Carter MJ, Eckert KA, Silva JC. Will the SAFE strategy be sufficient to eliminate trachoma by 2020? Puzzlements and possible solutions. The Scientific World Journal. 2013;2013(1):648106	Excluded	Excluded after title and abstract screening

172	Sarr B, Sissoko M, Fall M, Nizigama L, Cohn D, Willis R, Fuller B, O'Neil M, Solomon AW, Global Trachoma Mapping Project. Prevalence of trachoma in Senegal: Results of baseline surveys in 17 districts. Ophthalmic epidemiology. 2018 Dec 28;25(sup1):41-52.	Excluded	Excluded after title and abstract screening
173	Wu TJ, Reynolds MM. Trachoma, the world's leading infectious cause of blindness: The remaining gap in care and access to basic handwashing facilities. European Journal of Ophthalmology. 2023 Jul;33(4):1576-82	Excluded	Excluded after title and abstract screening
174	Travers AR. A Systematic Review Examining the Added Value of Water, Sanitation, and Hygiene Interventions for Preventive Chemotherapy Programs on Reducing the Prevalence of Trachoma	Excluded	Excluded after title and abstract screening
175	Callahan K, Emerson PM, Aragie S, Gebeyehu W, Freeman MC, Cevallos V, Melo J, Wittberg DM, Porco TC, Lietman TM. Sanitation, Water, and Instruction in Face-washing for Trachoma I.	Excluded	Excluded after title and abstract screening
176	Jones L, Gelaye B, Tilahun M, Belete H, Kumie A, Berhane Y, Williams MA. Access to water source, latrine facilities and other risk factors of active trachoma in Ankober, Ethiopia. PLoS One. 2009 Aug 20;4(8):e6702	Excluded	Excluded after title and abstract screening
177	Khan M. Eliminating Trachoma in Africa: The Importance of Environmental Interventions. Cureus. 2024 Jan;16(1).	Excluded	Excluded after title and abstract screening
178	M. Eliminating Trachoma in Africa: The Importance of Environmental Interventions. Cureus. 2024 Jan;16(1).	Excluded	Excluded after title and abstract screening
179	Wu TJ, Reynolds MM. Trachoma, the world's leading infectious cause of blindness: The remaining gap in care and access to basic handwashing facilities.	Excluded	Excluded after title and abstract screening

	European Journal of Ophthalmology. 2023 Jul;33(4):1576-82		
180	Abebe TA, Tucho GT. The impact of access to water supply and sanitation on the prevalence of active trachoma in Ethiopia: A systematic review and meta-analysis. PLoS Neglected Tropical Diseases. 2021 Sep 9;15(9):e0009644	Excluded	Excluded after title and abstract screening
181	Lynch M, Turner V, Munoz B, Rapoza P, Mmbaga BB, Taylor HR. Water availability and trachoma. Bulletin of the World Health Organization. 1989;67(1):71	Excluded	Excluded after title and abstract screening
182	Mwangi GW. Post-operative Trachomatous Trichiasis in Africa: a systematic review and online survey	Excluded	Excluded after title and abstract screening
183	Ogden S, Haddad D, Addiss DG, McGuire C, Freeman MC. Effect of water, sanitation, and hygiene on the prevention of trachoma: a systematic review and meta-analysis. PLoS medicine. 2014 Feb 25;11(2):e1001605	Excluded	Excluded after title and abstract screening
184	Alamrew Z, Feleke A, Fentie T, Atnafu K. Current state of active trachoma among elementary school students in the context of ambitious national growth plan: the case of Ethiopia. Health. 2013 Nov 11;2013	Excluded	Excluded after title and abstract screening
185	GD, Abebe S. Prevalence of risk factors for trachoma in a rural locality of Northwestern Ethiopia. East African medical journal. 2000;77(6	Excluded	Excluded after title and abstract screening
186	Worku A, Bejiga A, Adamu L, Alemayehu W, Bedri A, Haile Z, Ayalew A, Adamu Y, Gebre T, Kebede TD. Prevalence of trachoma in Ethiopia. The Ethiopian Journal of Health Development. 2007;21(3).	Excluded	Excluded after title and abstract screening
187	Wondimu A, Bejiga A. Prevalence of trachomatous trichiasis in the community of Alaba District, Southern Ethiopia. East African medical journal. 2003;80(7):365-9.	Excluded	Excluded after title and abstract screening

188	Wittberg DM, Tadesse W, Dagnew A, Hailu D, Chernet A, Melo JS, Aiemjoy K, Haile M, Zeru T, Tadesse Z. Water, sanitation, and hygiene for control of trachoma in Ethiopia (WUHA): a two-arm, parallel-group, cluster-randomised trial. The Lancet Global Health. 2022 Jan 1;10(1):e87-95.	Excluded	Excluded after title and abstract screening
189	Worku A, Kumie A, Yimer SA. Effect of water, sanitation and hygiene interventions on active trachoma in North and South Wollo zones of Amhara Region, Ethiopia: A Quasi-experimental study. PLoS neglected tropical diseases. 2017 Nov 10;11(11):e0006080.	Excluded	Excluded after title and abstract screening
190	Altherr FM, Nute AW, Zerihun M, Sata E, Stewart AE, Gessese D, Melak B, Astale T, Ayenew G, Callahan EK, Chanyalew M. Associations between Water, Sanitation and Hygiene (WASH) and trachoma clustering at aggregate	Excluded	Excluded after title and abstract screening
191	Greenland K, Czerniewska A, Guye M, Legesse D, Ahmed Mume A, Shafi Abdurahman O, Abraham Aga M, Miecha H, Shumi Bejiga G, Sarah V, Burton M. Seasonal variation in water use for hygiene in Oromia, Ethiopia, and its implications for trachoma control: An intensive observational study. PLoS neglected tropical diseases. 2022 May 13;16(5):e0010424	Excluded	Excluded after title and abstract screening
192	Tadesse B, Worku A, Kumie A, Yimer SA. The burden of and risk factors for active trachoma in the north and south Wollo zones of Amhara region, Ethiopia: a cross-sectional study. *Infect Dis Poverty*. 2017;**6**(1):143. doi: 10.1186/s40249-017-0358-3.	Excluded	Duplicated
193	Anteneh ZA, Getu WY. Prevalence of active trachoma and associated risk factors among children in Gazegibela district of Wagehemra Zone, Amhara	Excluded	Duplicated

	region, Ethiopia: community-based cross-sectional study. *Trop Dis Travel Med Vaccines.* 2016;**2**(1):5. doi: 10.1186/s40794-016-0022-0		
194	Gelaye B, Kumie A, Aboset N, Berhane Y, Williams MA. School-based intervention: evaluating the role of water, latrines and hygiene education on trachoma and intestinal parasitic infections in Ethiopia. Journal of water, sanitation and hygiene for development. 2014 Mar 1;4(1):120-30	Excluded	Duplicated
195	Kumie A, Aboset N, Berhane Y, Williams MA. School-based intervention: evaluating the role of water, latrines and hygiene education on trachoma and intestinal parasitic infections in Ethiopia. Journal of water, sanitation and hygiene for development. 2014 Mar 1;4(1):120-30.	Excluded	Duplicated
196	Smith JL, Sivasubramaniam S, Rabiu MM, Kyari F, Solomon AW, Gilbert C. Multilevel analysis of trachomatous trichiasis and corneal opacity in Nigeria: the role of environmental and climatic risk factors on the distribution of disease. PLoS neglected tropical diseases. 2015 Jul 29;9(7):e0003826	Excluded	Excluded after title and abstract screening
197	Aboset N, Berhane Y, Williams MA. School-based intervention: evaluating the role of water, latrines and hygiene education on trachoma and intestinal parasitic infections in Ethiopia. Journal of water, sanitation and hygiene for development. 2014 Mar 1;4(1):120-30	Excluded	Duplicated
198	Alebel A, Zegeye A, Tesfaye B, Wagnew F. Prevalence and associated factors of active trachoma among children in Ethiopia: a systematic review and meta-analysis. BMC infectious diseases. 2019 Dec;19:1-2.	Excluded	Excluded after title and abstract screening
199	Gupta N, Vashist P, Meel R, Grover S, Jain S, Kumar D, Gupta V, Tandon R, Solomon AW. Prospective	Excluded	Excluded after title and abstract screening

	audit of the phenotype, causes and correlates of trachomatous and non trachomatous trichiasis in a peri-elimination setting. PLOS Neglected Tropical Diseases. 2022 Dec 27;16(12):e0011014		
200	Matthews F, Reacher M, Baba S, Brayne C, Emerson P. Associations between active trachoma and community intervention with antibiotics, facial cleanliness, and environmental improvement (A, F, E). PLoS neglected tropical diseases. 2008 Apr 30;2(4):e229.	Excluded	Excluded after title and abstract screening
201	Kwami CS, Godfrey S, Gavilan H, Lakhanpaul M, Parikh P. Water, sanitation, and hygiene: linkages with stunting in rural Ethiopia. International journal of environmental research and public health. 2019 Oct;16(20):3793.	Excluded	Excluded due to the outcome of interest not reported
202	Yayemain D, King JD, Debrah O, Emerson PM, Aboe A, Ahorsu F, Wanye S, Ansah MO, Gyapong JO, Hagan M. Achieving trachoma control in Ghana after implementing the SAFE strategy. Transactions of the Royal Society of Tropical Medicine and Hygiene. 2009 Oct 1;103(10):993-1000.	Excluded	Conducted outside the eligible geographic area (Ethiopia)
203	Yallew WW, Terefe MW, Herchline TE, Sharma HR, Bitew BD, Kifle MW, Tetemke DM, Tefera MA, Adane MM. Assessment of water, sanitation, and hygiene practice and associated factors among people living with HIV/AIDS home based care services in Gondar city, Ethiopia. BMC Public Health. 2012 Dec;12:1-0.	Excluded	Excluded due to the outcome of interest not reported
204	Berhe AA, Aregay AD, Abreha AA, Aregay AB, Gebretsadik AW, Negash DZ, Gebreegziabher EG, Demoz KG, Fenta KA, Mamo NB. Knowledge, attitude, and practices on water, sanitation, and hygiene among rural residents in Tigray Region,	Excluded	Excluded due to the outcome of interest not reported

	Northern Ethiopia. Journal of Environmental and Public Health. 2020;2020(1):5460168.		
205	Aragie S, Wittberg DM, Tadesse W, Dagnew A, Hailu D, Chernet A, Melo JS, Aiemjoy K, Haile M, Zeru T, Tadesse Z. Water, sanitation, and hygiene for control of trachoma in Ethiopia (WUHA): a two-arm, parallel-group, cluster-randomised trial. The Lancet Global Health. 2022 Jan 1;10(1):e87-95.	Excluded	Excluded due to the outcome of interest not reported
206	Aschale A, Adane M, Getachew M, Faris K, Gebretsadik D, Sisay T, Dewau R, Chanie MG, Muche A, Zerga AA, Lingerew M. Water, sanitation, and hygiene conditions and prevalence of intestinal parasitosis among primary school children in Dessie City, Ethiopia. PloS one. 2021 Feb 3;16(2):e0245463.	Excluded	Excluded due to the outcome of interest not reported
207	Sahiledengle B, Petrucka P, Kumie A, Mwanri L, Beressa G, Atlaw D, Tekalegn Y, Zenbaba D, Desta F, Agho KE. Association between water, sanitation and hygiene (WASH) and child undernutrition in Ethiopia: a hierarchical approach. BMC Public Health. 2022 Oct 19;22(1):1943.	Excluded	Excluded due to the outcome of interest not reported
208	Debrah O, Mensah EO, Senyonjo L, de Souza DK, Hervie TE, Agyemang D, Bakajika D, Marfo B, Ahorsu F, Wanye S, Bailey R. Elimination of trachoma as a public health problem in Ghana: Providing evidence through a pre-validation survey. PLoS neglected tropical diseases. 2017 Dec 12;11(12):e0006099.	Excluded	Conducted outside the eligible geographic area (Ethiopia)
209	Gyasi ME, Nsiire A, Yayemain D, Debrah OA, Asamani D, Gyapong J, Amoaku WM. Trachoma in Northern Ghana: a need for further studies. Ophthalmic Epidemiology. 2010 Dec 1;17(6):343-8.	Excluded	Conducted outside the eligible geographic area (Ethiopia)
210	Senyonjo L, Aboe A, Bailey R, Agyemang D, Marfo B, Wanye S, Schmidt E, Addy J, Blanchet K.	Excluded	Conducted outside the eligible geographic area (Ethiopia)

	Operational adaptations of the trachoma pre-validation surveillance strategy employed in Ghana: a qualitative assessment of successes and challenges. Infectious diseases of poverty. 2019 Dec;8:1-1.		
211	Clegg EA. *Prevalence of Trachoma and Factors that Influence Transmission in Children in the Builsa District* (Doctoral dissertation, University of Ghana).	Excluded	Conducted outside the eligible geographic area (Ethiopia)
212	Girsha WD, Adlo AM, Garoma DA, Beggi SK. Assessment of water, sanitation and hygiene status of households in Welenchiti town, Boset Woreda, East Shoa Zone, Ethiopia. Sci J Public Health. 2016 Sep;4(6):435.	Excluded	Excluded due to the outcome of interest not reported
213	Senyonjo LG, Debrah O, Martin DL, Asante-Poku A, Migchelsen SJ, Gwyn S, Desouza DK, Solomon AW, Agyemang D, Biritwum-Kwadwo N, Marfo B. Serological and PCR-based markers of ocular Chlamydia trachomatis transmission in northern Ghana after elimination of trachoma as a public health problem. PLoS neglected tropical diseases. 2018 Dec 14;12(12):e0007027.	Excluded	Conducted outside the eligible geographic area (Ethiopia)
214	Abashawl A, Macleod C, Riang J, Mossisa F, Dejene M, Willis R, Flueckiger RM, Pavluck AL, Tadesse A, Adera TH, et al. Prevalence of Trachoma in Gambella Region, Ethiopia: Results of Three Population-Based Prevalence Surveys Conducted with the Global Trachoma Mapping Project. Ophthalmic Epidemiol. 2016;23(sup1):77–83	Excluded	Duplicated
215	Alemayehu M, Koye DN, Tariku A, Yimam K. Prevalence of active trachoma and its associated factors among rural and urban children in Dera Woreda Northwest Ethiopia: a comparative cross-sectional study. BioMed Res Int. 2015;2015	Excluded	Duplicated

216	Gebremichael SG, Yismaw E, Tsegaw BD, Shibeshi AD. Determinants of water source use, quality of water, sanitation and hygiene perceptions among urban households in North-West Ethiopia: A cross-sectional study. Plos one. 2021 Apr 22;16(4):e0239502.	Excluded	Excluded due to the outcome of interest not reported
217	Nega A, Aklilu AR, Abdosh AT, Jelalu KB, Eskindr DZ. Prevalence and factors associated with trachoma among primary school children in Harari region Eastern Ethiopia. Ophthalmol Res. 2017;7(3):1–9	Excluded	Duplicated
218	Debrah O, Mensah E, Senyonjo L, de Souza DK, Hervie TE, Agyemang D, Bakajika D, Marfo B, Ahorsu F, Wanye S, Koroma J. Pre-validation survey for the elimination of trachoma and evaluation of the effectiveness of the trachoma surveillance strategy in Ghana. ProtocolsIo Available: https://www. protocols. io/view/pre-validation-survey-for-the-elimination-of-trach-h9db926. 2017 Jun 27.	Excluded	Conducted outside the eligible geographic area (Ethiopia)
219	Emerson PM, Ngondi J, Biru E, Graves PM, Ejigsemahu Y, Gebre T, Endeshaw T, Genet A, Mosher AW, Zerihun M. Integrating an NTD with one of "the big three": combined malaria and trachoma survey in Amhara region of Ethiopia. PLoS Negl Trop Dis. 2008;2(3):e197	Excluded	Duplicated
220	Nega A, Aklilu AR, Abdosh AT, Jelalu KB, Eskindr DZ. Prevalence and factors associated with trachoma among primary school children in Harari region Eastern Ethiopia. Ophthalmol Res. 2017;7(3):1–9	Excluded	Duplicated
221	Zegeye M. Water, Sanitation, and Hygiene (WASH) in Ethiopia: An International Human Rights Framework. Journal of Health and Human Services Administration. 2024 Sep 26:10793739241277608.	Excluded	Excluded after title and abstract screening
222	Alemu F, Eba K, Bonger ZT, Youya A, Gerbaba MJ, Teklu AM, Medhin G. The effect of a health extension	Excluded	Excluded after title and abstract screening

	program on improving water, sanitation, and hygiene practices in rural Ethiopia. BMC Health Services Research. 2023 Aug 7;23(1):836.		
223	Phillips AE, Ower AK, Mekete K, Liyew EF, Maddren R, Belay H, Chernet M, Anjulo U, Mengistu B, Salasibew M, Tasew G. Association between water, sanitation, and hygiene access and the prevalence of soil-transmitted helminth and schistosome infections in Wolayita, Ethiopia. Parasites & Vectors. 2022 Nov 4;15(1):410.	Excluded	Excluded after title and abstract screening
224	Whitley L, Hutchings P, Cooper S, Parker A, Kebede A, Joseph S, Butterworth J, Van Koppen B, Mulejaa A. A framework for targeting water, sanitation and hygiene interventions in pastoralist populations in the Afar region of Ethiopia. International Journal of Hygiene and Environmental Health. 2019 Sep 1;222(8):1133-44.	Excluded	Excluded after title and abstract screening
225	Berihun G, Adane M, Walle Z, Abebe M, Alemnew Y, Natnael T, Andualem A, Ademe S, Tegegne B, Teshome D, Berhanu L. Access to and challenges in water, sanitation, and hygiene in healthcare facilities during the early phase of the COVID-19 pandemic in Ethiopia: A mixed-methods evaluation. PloS one. 2022 May 13;17(5):e0268272.	Excluded	Excluded after title and abstract screening
226	Tadesse B, Worku A, Kumie A, Yimer SA. Effect of water, sanitation and hygiene interventions on active trachoma in North and South Wollo zones of Amhara Region, Ethiopia: A Quasi-experimental study. PLoS neglected tropical diseases. 2017 Nov 10;11(11):e0006080.	Excluded	Excluded after title and abstract screening
227	Smith JL, Flueckiger RM, Hooper PJ, Polack S, Cromwell EA, Palmer SL, Emerson PM, Mabey DC, Solomon AW, Haddad D, Brooker SJ. The	Excluded	Conducted outside the eligible geographic area (Ethiopia)

	geographical distribution and burden of trachoma in Africa. PLoS neglected tropical diseases. 2013 Aug 8;7(8):e2359.		
228	Akyereko E, Ameme DK, Afari EA, Nyarko KM, Sackey S, Bandoh DA, Kenu E. Towards global elimination of Trachoma: An evaluation of Trachoma surveillance system, Wa East District, Ghana. Journal of Interventional Epidemiology and Public Health. 2022 Dec 27;5(24).	Excluded	Conducted outside the eligible geographic area (Ethiopia)
229	Amoabeng JK. *Risk Factors for Trachoma: A Case Control Study at Yaala Sub-District, Wa District, Upper West Region* (Doctoral dissertation, University of Ghana).	Excluded	Conducted outside the eligible geographic area (Ethiopia)
230	Amoabeng JK. *Risk Factors for Trachoma: A Case Control Study at Yaala Sub-District, Wa District, Upper West Region* (Doctoral dissertation, University of Ghana).	Excluded	Conducted outside the eligible geographic area (Ethiopia)
231	Renneker KK, Abdala M, Addy J, Al-Khatib T, Amer K, Badiane MD, Batcho W, Bella L, Bougouma C, Bucumi V, Chisenga T. Global progress toward the elimination of active trachoma: an analysis of 38 countries. The Lancet Global Health. 2022 Apr 1;10(4):e491-500.	Excluded	Conducted outside the eligible geographic area (Ethiopia)
232	King JD, Odermatt P, Utzinger J, Ngondi J, Bamani S, Kamissoko Y, Boubicar K, Hassan AS, Nwobi BC, Jip N, Amnie A. Trachoma among children in community surveys from four African countries and implications of using school surveys for evaluating prevalence. International health. 2013 Dec 1;5(4):280-7.	Excluded	Conducted outside the eligible geographic area (Ethiopia)
233	Antwi-Adjei EK. Relationship between the prevalence of trachomatous inflammation in children (age 1-9years) and the prevalence of trichiasis in adults (age 15years and above) at a presumed steady state.	Excluded	Conducted outside the eligible geographic area (Ethiopia)

234	Gebre T, Kello AB, Habtamu E, Ngondi JM. Trachoma. InNeglected Tropical Diseases-Sub-Saharan Africa 2024 Jun 8 (pp. 415-431). Cham: Springer International Publishing.	Excluded	Conducted outside the eligible geographic area (Ethiopia)
235	Ageed A, Khan M. Eliminating Trachoma in Africa: The Importance of Environmental Interventions. Cureus. 2024 Jan;16(1).	Excluded	Conducted outside the eligible geographic area (Ethiopia)
235	Hagan M, Yayemain D, Ahorsu F, Aboe A. Prevalence of active trachoma two years after control activities. Ghana Medical Journal. 2009;43(2).	Excluded	Conducted outside the eligible geographic area (Ethiopia)
236	Polack S, Brooker S, Kuper H, Mariotti S, Mabey D, Foster A. Mapping the global distribution of trachoma. Bulletin of the World Health Organization. 2005 Dec;83(12):913-9.	Excluded	Conducted outside the eligible geographic area (Ethiopia)
237	Kanyi S, Hydara A, Sillah A, Mpyet C, Harte A, Bakhtiari A, Willis R, Jimenez C, Aboe A, Bailey R, Harding-Esch EM. The Gambia Trachomatous Trichiasis Surveys: Results from Five Evaluation Units Confirm Attainment of Trachoma Elimination Thresholds. Ophthalmic epidemiology. 2023 Jul 2:1-9.	Excluded	Conducted outside the eligible geographic area (Ethiopia)
238	Lietman TM, Oldenburg CE, Keenan JD. Trachoma: time to talk eradication. Ophthalmology. 2020 Jan 1;127(1):11-3.	Excluded	Conducted outside the eligible geographic area (Ethiopia)
239	Adah OS, Zoakah IA, Ogbonna C, Obadiah M, Ogiri S, Shown LD, Envuladu EA, Banwat ME, Bupwatda P. The effect of health education and antibiotic use on the prevalence of Trachoma in school children in Borno State of Northern Nigeria. Jos Journal of Medicine. 2010;5(1):38-42.	Excluded	Conducted outside the eligible geographic area (Ethiopia)
240	Rotondo LA, Ngondi J, Rodgers AF, King JD, Kamissoko Y, Amadou A, Jip N, Cromwell EA, Emerson PM. Evaluation of community intervention with pit latrines for trachoma control in Ghana, Mali,	Excluded	Conducted outside the eligible geographic area (Ethiopia)

	Niger and Nigeria. International Health. 2009 Dec 1;1(2):154-62.		
241	Girmay AM, Alemu ZA, Mengesha SD, Dinssa DA, Wagari B, Weldegebriel MG, Serte MG, Alemayehu TA, Kenea MA, Weldetinsae A, Teklu KT. Effect of demographic disparities on the use of the JMP ladders for water, sanitation, and hygiene services in Bishoftu Town, Ethiopia. Discover Water. 2022 Sep 12;2(1):8.	Excluded	Excluded after title and abstract screening
242	World Health Organization. Achieving quality universal health coverage through better water, sanitation and hygiene services in health care facilities: a focus on Ethiopia. World Health Organization; 2017.	Excluded	Excluded after title and abstract screening
243	Emerson LE, Anantharam P, Yehuala FM, Bilcha KD, Tesfaye AB, Fairley JK. Poor WASH (water, sanitation, and hygiene) conditions are associated with leprosy in North Gondar, Ethiopia. International journal of environmental research and public health. 2020 Sep;17(17):6061.	Excluded	Excluded after title and abstract screening
244	Tamene A, Afework A. Exploring barriers to the adoption and utilization of improved latrine facilities in rural Ethiopia: An Integrated Behavioral Model for Water, Sanitation and Hygiene (IBM-WASH) approach. PloS one. 2021 Jan 11;16(1):e0245289.	Excluded	Excluded after title and abstract screening
245	Wagari S, Girma H, Geremew A. Water, sanitation, and hygiene service ladders and childhood diarrhea in haramaya demographic and health surveillance site, eastern Ethiopia. Environmental Health Insights. 2022 Apr;16:11786302221091416.	Excluded	Excluded after title and abstract screening
246	Gebrehiwot T, Geberemariyam BS, Gebretsadik T, Gebresilassie A. Prevalence of diarrheal diseases among schools with and without water, sanitation and hygiene programs in rural communities of north-	Excluded	Excluded after title and abstract screening

	eastern Ethiopia: a comparative cross-sectional study. Rural and Remote Health. 2020 Dec;20(4):1-9.		
247	Sahiledengle B, Agho K. Determinants of childhood diarrhea in households with improved water, sanitation, and hygiene (WASH) in Ethiopia: evidence from a repeated cross-sectional study. Environmental Health Insights. 2021 Jun;15:11786302211025180.	Excluded	Excluded after title and abstract screening
248	Sahiledengle B, Agho K. Determinants of childhood diarrhea in households with improved water, sanitation, and hygiene (WASH) in Ethiopia: evidence from a repeated cross-sectional study. Environmental Health Insights. 2021 Jun;15:11786302211025180.	Excluded	Excluded after title and abstract screening
249	Girmay AM, Mengesha SD, Dinssa DA, Alemu ZA, Wagari B, Weldegebriel MG, Serte MG, Alemayehu TA, Kenea MA, Weldetinsae A, Teklu KT. Access to water, sanitation and hygiene (WASH) services and drinking water contamination risk levels in households of Bishoftu Town, Ethiopia: A cross-sectional study. Health Science Reports. 2023 Nov;6(11):e1662.	Excluded	Excluded after title and abstract screening
250	Admasie A, Debebe A. Estimating access to drinking water supply, sanitation, and hygiene facilities in Wolaita Sodo town, southern Ethiopia, in reference to national coverage. Journal of Environmental and Public Health. 2016;2016(1):8141658.	Excluded	Excluded after title and abstract screening
251	Admasie A, Debebe A. Estimating access to drinking water supply, sanitation, and hygiene facilities in Wolaita Sodo town, southern Ethiopia, in reference to national coverage. Journal of Environmental and Public Health. 2016;2016(1):8141658.	Excluded	Excluded after title and abstract screening
252	Abera B, Mulu W, Yizengaw E, Hailu T, Kibret M. Water safety, sanitation and hygiene related knowledge, attitudes and practices among household	Excluded	Excluded after title and abstract screening

	residents in peri-urban areas in Northwest Ethiopia. Ethiopian Journal of Health Development. 2018;32(3).		
253	Abera B, Mulu W, Yizengaw E, Hailu T, Kibret M. Water safety, sanitation and hygiene related knowledge, attitudes and practices among household residents in peri-urban areas in Northwest Ethiopia. Ethiopian Journal of Health Development. 2018;32(3).	Excluded	Excluded after title and abstract screening
254	Derso A, Addis T, Mengistie B. Healthcare facility water, sanitation, and hygiene service status and barriers in Addis Ababa, Ethiopia. Journal of Water, Sanitation and Hygiene for Development. 2023 Dec 1;13(12):1001-17.	Excluded	Excluded after title and abstract screening
255	Hailu T, Wondemagegn MU, Abera B. Effects of water source, sanitation and hygiene on the prevalence of Schistosoma mansoni among school age children in Jawe District, Northwest Ethiopia. Iranian Journal of Parasitology. 2020 Jan;15(1):124.	Excluded	Excluded after title and abstract screening
256	Soboksa NE, Hailu AB, Gari SR, Alemu BM. Water supply, sanitation and hygiene interventions and childhood diarrhea in Kersa and Omo Nada districts of Jimma Zone, Ethiopia: a comparative cross-sectional study. Journal of Health, Population and Nutrition. 2019 Dec;38:1-4.	Excluded	Excluded after title and abstract screening
257	Hailu D, Jeon Y, Gedefaw A, Kim JH, Mraidi R, Getahun T, Mogeni OD, Mesfin Getachew E, Jang GH, Mukasa D, Pak GD. Dissecting Water, Sanitation, and Hygiene (WaSH) to Assess Risk Factors for Cholera in Shashemene, Oromia Region, Ethiopia. Clinical Infectious Diseases. 2024 Jul 15;79(Supplement_1):S53-62.	Excluded	Excluded after title and abstract screening
258	Head JR, Pachón H, Tadesse W, Tesfamariam M, Freeman MC. Integration of water, sanitation, hygiene and nutrition programming is associated with lower	Excluded	Excluded after title and abstract screening

	prevalence of child stunting and fever in Oromia, Ethiopia.		
259	Aydamo AA, Gari SR, Mereta ST. Access to drinking water, sanitation, and hand hygiene facilities in the peri-urban and informal settlements of Hosanna Town, Southern Ethiopia. Environmental health insights. 2023 Aug;17:11786302231193604.	Excluded	Excluded after title and abstract screening
260	Amsalu A, Atikilt G, Taklual W, Tilahun A. Progress of water, sanitation, and hygiene facilities in public primary schools of south Gondar zone, Northern Ethiopia. PAMJ-One Health. 2022 Feb 22;7(19).	Excluded	Excluded after title and abstract screening
261	Mpyet C, Goyol M, Ogoshi C. Personal and environmental risk factors for active trachoma in children in Yobe state, north-eastern Nigeria. Tropical Medicine & International Health. 2010 Feb;15(2):168-72.	Excluded	Conducted outside the eligible geographic area (Ethiopia)
262	Mansur R, Muhammad N, Liman IR. Prevalence and magnitude of trachoma in a local government area of Sokoto State, north western Nigeria. Nigerian Journal of Medicine. 2007;16(4):348-53.	Excluded	Conducted outside the eligible geographic area (Ethiopia)
263	Ramyil A, Wade P, Ogoshi C, Goyol M, Adenuga O, Dami N, Mpyet C. Prevalence of trachoma in Jigawa state, Northwestern Nigeria. Ophthalmic epidemiology. 2015 May 4;22(3):184-9.	Excluded	Conducted outside the eligible geographic area (Ethiopia)
264	Mpyet C, Muhammad N, Adamu MD, Muazu H, Umar MM, Goyol M, Onyebuchi U, Chima I, Idris H, William A, Isiyaku S. Prevalence of trachoma in Katsina State, Nigeria: results of 34 district-level surveys. Ophthalmic epidemiology. 2016 Dec 7;23(sup1):55-62.	Excluded	Conducted outside the eligible geographic area (Ethiopia)
265	Muhammad N, Mohammed A, Isiyaku S, Adamu MD, Gwom A, Rabiu MM. Mapping trachoma in 25 local government areas of Sokoto and Kebbi states,	Excluded	Conducted outside the eligible geographic area (Ethiopia)

	northwestern Nigeria. British Journal of Ophthalmology. 2014 Apr 1;98(4):432-7.		
266	Mpyet C, Muhammad N, Adamu MD, Muazu H, Umar MM, Abdull M, Alada J, Goyol M, Onyebuchi U, Olamiju F, Isiyaku S. Prevalence of trachoma in Bauchi State, Nigeria: results of 20 local government area-level surveys. Ophthalmic epidemiology. 2016 Dec 7;23(sup1):39-45.	Excluded	Conducted outside the eligible geographic area (Ethiopia)
267	Hammou J, El Ajaroumi H, Hasbi H, Nakhlaoui A, Hmadna A, El Maaroufi A. In Morocco, the elimination of trachoma as a public health problem becomes a reality. The Lancet Global Health. 2017 Mar 1;5(3):e250-1.	Excluded	Conducted outside the eligible geographic area (Ethiopia)
268	Hammou J, Obtel M, Razine R, Berraho A, Belmekki M. Elimination of trachoma from Morocco: a historical review. Eastern Mediterranean Health Journal. 2020;26(6):713-9.	Excluded	Conducted outside the eligible geographic area (Ethiopia)
269	Hammou J, Guagliardo SA, Obtel M, Razine R, Haroun AE, Youbi M, Bellefquih AM, White M, Gwyn S, Martin DL. Post-validation survey in two districts of Morocco after the elimination of trachoma as a public health problem. The American Journal of Tropical Medicine and Hygiene. 2022 May;106(5):1370.	Excluded	Conducted outside the eligible geographic area (Ethiopia)
270	Mohammadpour M, Abrishami M, Masoumi A, Hashemi H. Trachoma: Past, present and future. Journal of current ophthalmology. 2016 Dec 1;28(4):165-9.	Excluded	Conducted outside the eligible geographic area (Ethiopia)
271	Tadesse B, Worku A, Kumie A, Yimer SA. Effect of water, sanitation and hygiene interventions on active trachoma in North and South Wollo zones of Amhara Region, Ethiopia: A Quasi-experimental study. PLoS	Excluded	Non-observational study design

	neglected tropical diseases. 2017 Nov 10;11(11):e0006080.		
272	Bengtson IA. The epithelial cell inclusions of trachoma: Experimental studies. American Journal of Ophthalmology. 1929 Aug 1;12(8):637-50.	Excluded	Non-observational study design
273	Grayston JT, Wang SP, Yang YF, Woolridge RL. The effect of trachoma virus vaccine on the course of experimental trachoma infection in blind human volunteers. The Journal of experimental medicine. 1962 May 1;115(5):1009-22.	Excluded	Non-observational study design
274	Grayston JT, Wang SP, Woolridge RL, Yang YF, Johnston PB. Trachoma-studies of etiology, laboratory diagnosis, and prevention. Journal of the American Medical Association. 1960 Apr 9;172(15):1577-86.	Excluded	Non-observational study design
275	Noguchi H, Cohen M. EXPERIMENTS ON THE CULTIVATION OF SO CALLED TRACHOMA BODIES. The Journal of Experimental Medicine. 1913 Nov 1;18(5):572-8.	Excluded	Non-observational study design
276	Olitsky PK, Knutti RE, Tyler JR. THE EFFECT OF SECONDARY INFECTIONS ON EXPERIMENTAL TRACHOMA. The Journal of Experimental Medicine. 1931 May 1;53(5):753-62.	Excluded	Non-observational study design
277	Chang HL, Chin HY, Wang KO. EXPERIMENTAL TRACHOMA IN HUMAN VOLUNTEERS PRODUCFD BY CULTURED VIRUS. Chinese Medical Journal. 1960;80(03):214-21.	Excluded	Non-observational study design
278	Grayston JT. Immunization against trachoma. InFirst international conference on vaccine against viral and rickettsial diseases of man. Pan American Health Organization Scientific Publication 1967 May (Vol. 147, pp. 546-9).	Excluded	Non-observational study design
279	THYGESON P. Acute trachoma. Archives of Ophthalmology. 1949 Nov 1;42(5):655-65.	Excluded	Non-observational study design

280	Reimann HA, Pillat A. Studies on the Etiology of Trachoma. The Journal of Experimental Medicine. 1931 May 1;53(5):687-94.	Excluded	Non-observational study design
281	Tiruneh M, Woldeyohannes D, Muluye D. Active trachoma and associated risk factors among children in Baso Liben District of East Gojjam Ethiopia. *BMC Public Health.* 2012;**12**(1):1105. doi: 10.1186/1471-2458-12-1105	Excluded	Published outside the eligible date range (2019–2024)
282	Worku A, Kumie A, Yimer SA. The burden of and risk factors for active trachoma in the north and south Wollo zones of Amhara region, Ethiopia: a cross-sectional study. *Infect Dis Poverty*. 2017;**6**(1):143. doi: 10.1186/s40249-017-0358-3	Excluded	Published outside the eligible date range (2019–2024)
283	Berhe R, Gedefaw M. Prevalence and associated factors of active trachoma among childeren aged 1–9 years in rural communities of Gonji Kolella district, West Gojjam zone North West Ethiopia. *BMC Res Notes.* 2015;**8**(1):641. doi: 10.1186/s13104-015-1529-6	Excluded	Published outside the eligible date range (2019–2024)
284	Moges HG. Risk factors for active trachoma among children aged 1-9 years in Maksegnit town, Gondar Zuria District Northwest Ethiopia. *Risk.* 2013;**2**(3):202–20	Excluded	Published outside the eligible date range (2019–2024)
285	Shiferaw A, Alamrew Z, Feleke A, Fentie T, Atnafu K: Current state of active trachoma among elementary school students in the context of ambitious national growth plan: The case of Ethiopia. Health (NY) 2013, 5(11):1768.	Excluded	Published outside the eligible date range (2019–2024)
286	Stewart AE, Kramer MR, Endeshaw T, Zerihun M, Melak B, Sata E, Gessese D, Teferi T, Tadesse Z. Active trachoma and community use of sanitation Ethiopia. Bull World Health Organ. 2017;95(4):250	Excluded	Published outside the eligible date range (2019–2024)

287	Graves PM, Ejigsemahu Y, Gebre T, Endeshaw T, Genet A, Mosher AW, Zerihun M. Integrating an NTD with one of “the big three”: combined malaria and trachoma survey in Amhara region of Ethiopia. PLoS Negl Trop Dis. 2008;2(3):e197	Excluded	Published outside the eligible date range (2019–2024)
288	Macleod C, Adamu Y, Ahmed M, Ibrahim M, Ali M, Haileselassie T, Willis R, Chu BK, Dejene M. Prevalence of trachoma in the Afar Region of Ethiopia: results of seven population-based surveys from the Global Trachoma	Excluded	Published outside the eligible date range (2019–2024)
289	Negussu Ayele N, Macleod CK, Kello AB, Eshetu Gezachew Z, Binegdie A, Dejene M, Alemayehu W, Flueckiger RM, Massae PA. Epidemiology of trachoma and its implications for implementing the “SAFE” strategy in Somali Region, Ethiopia: results of 14 population-based prevalence surveys. Ophthalmic Epidemiol. 2018;25(sup1):25–32.	Excluded	Published outside the eligible date range (2019–2024)
290	Wosen A, Bekana F, Ayanos T. Prevalence of trachoma and associated risk factors among Yello elementary school students. Loma Woreda, Dawro Zone, Ethiopia: J Nursing care; 2015.	Excluded	Published outside the eligible date range (2019–2024)
291	Sherief ST, Macleod C, Gigar G, Godefay H, Abraha A, Dejene M, Kello AB, Belete A, Assefa Y, Willis R, et al. The Prevalence of Trachoma in TigrayRegion, Northern Ethiopia: Results of 11 Population-Based Prevalence Surveys Completed as Part of the Global Trachoma Mapping Project. Ophthalmic Epidemiol. 2016;23(sup1):94–9	Excluded	Published outside the eligible date range (2019–2024)
292	Haftu D, Gebremichael G, Ajema D, Gedamu G, Agedew E. Water, sanitation and hygiene practice and associated factors among HIV infected people in Arba Minch town, Southern Ethiopia. Journal of water and health. 2017 Aug 1;15(4):615-25.	Excluded	Excluded after title and abstract screening

293	Regassa N, Rajan DS, Ketsela K. Access to, and utilization of information on sanitation and hygiene by rural households in Alaba special district, Southern Ethiopia. Journal of Human Ecology. 2011 Feb 1;33(2):101-12.	Excluded	Excluded after title and abstract screening
294	Beyene A, Hailu T, Faris K, Kloos H. Current state and trends of access to sanitation in Ethiopia and the need to revise indicators to monitor progress in the Post-2015 era. BMC public health. 2015 Dec;15:1-8.	Excluded	Excluded after title and abstract screening
295	Tesema KA, Haimanot AB. Knowledge, attitude and practice towards water, sanitation and hygiene (WASH) among holy water users in Amhara regional state Orthodox churches, Ethiopia, 2024: institutional based cross-sectional study. Journal of Water and Health. 2024 Jul 1;22(7):1282-92.	Excluded	Excluded after title and abstract screening
296	Tamene A. What it takes to save lives: An assessment of water, sanitation, and hygiene facilities in temporary COVID-19 isolation and treatment centers of Southern Ethiopia: A mixed-methods evaluation. PLoS One. 2021 Aug 13;16(8):e0256086.	Excluded	Excluded after title and abstract screening
297	Gizaw Z, Worku A. Effects of single and combined water, sanitation and hygiene (WASH) interventions on nutritional status of children: a systematic review and meta-analysis. Italian journal of pediatrics. 2019 Dec;45:1-4.	Excluded	Excluded after title and abstract screening
298	Woldesenbet B, Tolcha A, Tsegaye B. Water, hygiene and sanitation practices are associated with stunting among children of age 24-59 months in Lemo district, South Ethiopia, in 2021: community based cross sectional study. BMC nutrition. 2023 Jan 23;9(1):17.	Excluded	Excluded after title and abstract screening
299	Kassie GG, Hayelom DH. Assessment of water handling and sanitation practices among rural communities of Farta Woreda, Northwest Ethiopia.	Excluded	Excluded after title and abstract screening

	American Journal of Health Research. 2017 Aug;5(5):119-24.		
300	Gizaw Z, Adane T, Azanaw J, Addisu A, Haile D. Childhood intestinal parasitic infection and sanitation predictors in rural Dembiya, northwest Ethiopia. Environmental health and preventive medicine. 2018 Dec;23:1-0.	Excluded	Excluded after title and abstract screening
301	West ES, Munoz B, Imeru A, Alemayehu W, Melese M, West SK. The association between epilation and corneal opacity among eyes with trachomatous trichiasis. British journal of ophthalmology. 2006 Feb 1;90(2):171-4.	Excluded	Excluded due to the outcome of interest not reported
302	Worku EM, Fekadu SA, Alemie BW, Lorato MM. Prevalence and associated factors of corneal opacity among adults in Kolladiba town, Northwest Ethiopia: a cross-sectional study. BMJ Open Ophthalmology. 2024 Apr 1;9(1):e001665.	Excluded	Excluded due to the outcome of interest not reported
303	Burn H, Aweke S, Wondie T, Habtamu E, Deribe K, Rajak S, Bremner S, Davey G. Podoconiosis, trachomatous trichiasis and cataract in northern Ethiopia: A comparative cross sectional study. PLoS neglected tropical diseases. 2017 Feb 10;11(2):e0005388.	Excluded	Excluded due to the outcome of interest not reported
304	Ngondi J, Matthews F, Reacher M, Baba S, Brayne C, Emerson P. Associations between active trachoma and community intervention with antibiotics, facial cleanliness, and environmental improvement (A, F, E). PLoS neglected tropical diseases. 2008 Apr 30;2(4):e229.	Excluded	Duplicated
305	Esmael Habtamu EH, Rajak SN, Zerihun Tadesse ZT, Tariku Wondie TW, Mulat Zerihun MZ, Birhan Guadie BG, Teshome Gebre TG, Amir Bedri Kello AB, Callahan K, Mabey DC, Khaw PT. Epilation for	Excluded	Excluded due to the outcome of interest not reported

	minor trachomatous trichiasis: four-year results of a randomised controlled trial.		
306	Shaffi M, Bejiga A. Common eye diseases in children of rural community in Goro district, Central Ethiopia. Ethiopian Journal of Health Development. 2005 Oct 27;19(2):148-52.	Excluded	Excluded by title and abstract screening
307	Rajak SN, Habtamu E, Weiss HA, Bedri A, Gebre T, Bailey RL, Mabey DC, Khaw PT, Gilbert CE, Emerson PM, Burton MJ. The clinical phenotype of trachomatous trichiasis in Ethiopia: not all trichiasis is due to entropion. Investigative ophthalmology & visual science. 2011 Oct 1;52(11):7974-80.	Excluded	Excluded due to the outcome of interest not reported
308	Meshesha TD, Senbete GH, Bogale GG. Determinants for not utilizing trachomatous trichiasis surgery among trachomatous trichiasis patients in Mehalsayint District, North-East Ethiopia. PLoS neglected tropical diseases. 2018 Jul 18;12(7):e0006669.	Excluded	Excluded due to the outcome of interest not reported
309	Rajak SN, Habtamu E, Weiss HA, Kello AB, Gebre T, Genet A, Bailey RL, Mabey DC, Khaw PT, Gilbert CE, Emerson PM. Absorbable versus silk sutures for surgical treatment of trachomatous trichiasis in Ethiopia: a randomised controlled trial. PLoS medicine. 2011 Dec 13;8(12):e1001137.	Excluded	Excluded due to the outcome of interest not reported
310	Nyamwaro CM. Prevalence, and Risk Factors for Trachoma Infection Among Children Aged 1–9 Years Old in Oldonyonyokie Location, Magadi Division, Kajiado County, Ethiopia	Excluded	Duplicated
311	Elmajri KA, Abdolmejed F. Trachoma Diseases in conflict-affected areas Derna city eastern part of Libya Review of Their Prevalence, Distribution, and Disease Burden. Ophthalmology and Vision Care. 2023 Mar 4;3(1).	Excluded	Conducted outside the eligible geographic area (Ethiopia)

312	Ben-Amer MI. Trachoma and Some Other Eye Diseases in a Libyan Village. University of Glasgow (United Kingdom); 1983.	Excluded	
313	Smith JL, Flueckiger RM, Hooper PJ, Polack S, Cromwell EA, Palmer SL, Emerson PM, Mabey DC, Solomon AW, Haddad D, Brooker SJ. The geographical distribution and burden of trachoma in Africa. PLoS neglected tropical diseases. 2013 Aug 8;7(8):e2359.	Excluded	Conducted outside the eligible geographic area (Ethiopia)
314	World Health Organization. WHO Alliance for the Global Elimination of Blinding Trachoma by the year 2020: Progress report on elimination of trachoma, 2013. Weekly Epidemiological Record= Relevé épidémiologique hebdomadaire. 2014;89(39):421-8.	Excluded	Outcome not reported
315	Altajori NN, Elshrek YM. risk factors for non-communicable diseases in Libya. The Egyptian Journal of Hospital Medicine. 2017 Jan 1;66(1):202-14.	Excluded	Conducted outside the eligible geographic area (Ethiopia)
316	Mpyet C, Ogoshi C, Goyol M. Prevalence of trachoma in Yobe State, north-eastern Nigeria. Ophthalmic epidemiology. 2008 Jan 1;15(5):303-7.	Excluded	
317	Mpyet CD, Olobio N, Isiyaku S, Wamyil-Mshelia T, Ajege G, Ogoshi C, Olamiju F, Achu I, Adamu MD, Muhammad N, Jabo AM. Progress towards the elimination of trachoma in Nigeria. International Health. 2024 May 31:ihae035.	Excluded	Duplicated
318	Sarsour A, Al Shaarawi S. Water scarcity and trachoma prevalence in Gaza Strip, Palestine: possibilities and precautions according to WHO SAFE strategy for trachoma control. International Journal of Pharmacy Teaching & Practices. 2013;4:622-30.	Excluded	Conducted outside the eligible geographic area (Ethiopia)
319	mondiale de la Santé O, World Health Organization. Weekly Epidemiological Record, 2022, vol. 97, 31 [full issue]. Weekly Epidemiological Record= Relevé	Excluded	Outcome not reported

	épidémiologique hebdomadaire. 2022 Aug 5;97(31):353-64.		
320	Worku EM, Fekadu SA, Alemie BW, Lorato MM. Prevalence and associated factors of corneal opacity among adults in Kolladiba town, Northwest Ethiopia: a cross-sectional study. BMJ Open Ophthalmology. 2024 Apr 1;9(1):e001665.	Excluded	Excluded by title and abstract screening
321	Mehari ZA. Pattern of childhood ocular morbidity in rural eye hospital, Central Ethiopia. BMC ophthalmology. 2014 Dec;14:1-6.	Excluded	Excluded by title and abstract screening
322	Shaffi M, Bejiga A. Common eye diseases in children of rural community in Goro district, Central Ethiopia. Ethiopian Journal of Health Development. 2005 Oct 27;19(2):148-52.	Excluded	Excluded by title and abstract screening
323	Shaffi M, Bejiga A. Common eye diseases in children of rural community in Goro district, Central Ethiopia. Ethiopian Journal of Health Development. 2005 Oct 27;19(2):148-52.	Excluded	Excluded by title and abstract screening
324	Worku Y, Bayu S. Screening for ocular abnormalities and subnormal vision in school children of Butajira Town, southern Ethiopia. Ethiopian Journal of Health Development. 2002;16(2):165-71.	Excluded	Excluded by title and abstract screening
325	Demissie BS, Demissie ES. Patterns of eye diseases in children visiting a tertiary teaching hospital: South-western Ethiopia. Ethiopian journal of health sciences. 2014 Apr 15;24(1):69-74.	Excluded	Excluded by title and abstract screening
326	Negussie D, Tilahun Y. Pattern of corneal blindness in Goro district, Gurage zone, southern Ethiopia. The Ethiopian Journal of Health Development. 2008;22(3).	Excluded	Excluded by title and abstract screening
327	Gelaw Y, Abateneh A. Ocular morbidity among refugees in Southwest Ethiopia. Ethiopian Journal of Health Sciences. 2014 Jul 31;24(3):227-34.	Excluded	Excluded by title and abstract screening

328	Gelaw Y, Abateneh A. Ocular morbidity among refugees in Southwest Ethiopia. Ethiopian Journal of Health Sciences. 2014 Jul 31;24(3):227-34.	Excluded	
329	Tolla TT. Visually impaired children in some sites of Ethiopia: Review on prevalence, causes and problems. Bahir Dar Journal of Education. 2016;16(1).	Excluded	Excluded by title and abstract screening
330	Ndisabiye D, Gahungu A, Kayugi D, Waters EK. Association of environmental risk factors and trachoma in Gashoho Health District, Burundi. African Health Sciences. 2020 Apr 20;20(1):182-9.	Excluded	Conducted outside the eligible geographic area (Ethiopia)
331	Ndayishimiye O, Willems J, Manirakiza E, Smith JL, Gashikanyi R, Kariyo L, Ndayishimiye S, Niyoniziziye B, Niyonkuru A, Nkunda A, Deville MA. Population-based survey of active trachoma in 11 districts of Burundi. Ophthalmic epidemiology. 2011 Aug 1;18(4):146-9.	Excluded	Conducted outside the eligible geographic area (Ethiopia)
332	Tolla TT. Visually impaired children in some sites of Ethiopia: Review on prevalence, causes and problems. Bahir Dar Journal of Education. 2016;16(1).	Excluded	Duplicated
333	Renneker KK, Abdala M, Addy J, Al-Khatib T, Amer K, Badiane MD, Batcho W, Bella L, Bougouma C, Bucumi V, Chisenga T. Global progress toward the elimination of active trachoma: an analysis of 38 countries. The Lancet Global Health. 2022 Apr 1;10(4):e491-500.	Excluded	Conducted outside the eligible geographic area (Ethiopia)
334	Senyonjo L, Downs P, Schmidt E, Bailey R, Blanchet K. Lessons learned for surveillance strategies for trachoma elimination as a public health problem, from the evaluation of approaches utilised by Guinea worm and onchocerciasis programmes: A literature review. PLoS neglected tropical diseases. 2021 Jan 28;15(1):e0009082.	Excluded	Conducted outside the eligible geographic area (Ethiopia)

335	Gebreselassie G, Negash K, Tsegaye S, Makonnen M, Deneke B, Desalegn M, Harding-Esch EM, Harte A, Solomon AW, Boyd S, Bakhtiari A. Prevalence of trachoma in Somali region, Ethiopia: results from trachoma impact surveys in 50 woredas. International Health. 2023 Dec;15(Supplement_2):ii30-7.	Excluded	Conducted outside the eligible geographic area (Ethiopia)
336	Duale AB, Negussu Ayele N, Macleod CK, Kello AB, Eshetu Gezachew Z, Binegdie A, Dejene M, Alemayehu W, Flueckiger RM, Massae PA, Willis R. Epidemiology of trachoma and its implications for implementing the "SAFE" strategy in Somali Region, Ethiopia: results of 14 population-based prevalence surveys. Ophthalmic epidemiology. 2018 Dec 28;25(sup1):25-32.	Excluded	Conducted outside the eligible geographic area (Ethiopia)
337	World Health Organization. Address by Dr Hussein A. Gezairy, Regional Director, WHO Eastern Mediterranean Region, to the intercountry meeting on regional planning workshop for the elimination of blinding trachoma, Cairo, Egypt, 28-30 November 2005.	Excluded	Conducted outside the eligible geographic area (Ethiopia)
338	Olobio N, Isiyaku S, Wamyil-Mshelia T, Ajege G, Ogoshi C, Olamiju F, Achu I, Adamu MD, Muhammad N, Jabo AM. Progress towards the elimination of trachoma in Nigeria. International Health. 2024 May 31:ihae035	Excluded	Conducted outside the eligible geographic area (Ethiopia)
339	Jip NF, King JD, Diallo MO, Miri ES, Hamza AT, Ngondi J, Emerson PM. Blinding trachoma in Katsina state, Nigeria: population-based prevalence survey in ten local government areas. Ophthalmic epidemiology. 2008 Jan 1;15(5):294-302	Excluded	Duplicated
340	Mohammadpour M, Abrishami M, Masoumi A, Hashemi H. Trachoma: Past, present and future.	Excluded	Excluded after title and abstract screening

	Journal of current ophthalmology. 2016 Dec 1;28(4):165-9		
341	Rabiu MM, Muhammed N, Isiyaku S. Challenges of trachoma control: An assessment of the situation in Northern Nigeria. Middle East African journal of ophthalmology. 2011 Apr 1;18(2):115-22.	Excluded	Excluded after title and abstract screening
342	Clements AC, Kur LW, Gatpan G, Ngondi JM, Emerson PM, Lado M, Sabasio A, Kolaczinski JH. Targeting trachoma control through risk mapping: the example of Southern Sudan. PLoS Neglected Tropical Diseases. 2010 Aug 17;4(8):e799.	Excluded	Excluded after title and abstract screening
343	Greenland K, Czerniewska A, Guye M, Legesse D, Ahmed Mume A, Shafi Abdurahman O, Abraham Aga M, Miecha H, Shumi Bejiga G, Sarah V, Burton M. Seasonal variation in water use for hygiene in Oromia, Ethiopia, and its implications for trachoma control: An intensive observational study. PLoS neglected tropical diseases. 2022 May 13;16(5):e0010424.	Excluded	Excluded after title and abstract screening
344	Gelaye B, Kumie A, Aboset N, Berhane Y, Williams MA. School-based intervention: evaluating the role of water, latrines and hygiene education on trachoma and intestinal parasitic infections in Ethiopia. Journal of water, sanitation and hygiene for development. 2014 Mar 1;4(1):120-30.	Excluded	Excluded after title and abstract screening
345	Gelaye B, Kumie A, Aboset N, Berhane Y, Williams MA. School-based intervention: evaluating the role of water, latrines and hygiene education on trachoma and intestinal parasitic infections in Ethiopia. Journal of water, sanitation and hygiene for development. 2014 Mar 1;4(1):120-30.	Excluded	Excluded after title and abstract screening
345	Smith JL, Sivasubramaniam S, Rabiu MM, Kyari F, Solomon AW, Gilbert C. Multilevel analysis of trachomatous trichiasis and corneal opacity in Nigeria:	Excluded	Excluded after title and abstract screening

	the role of environmental and climatic risk factors on the distribution of disease. PLoS neglected tropical diseases. 2015 Jul 29;9(7):e0003826.		
346	Gupta N, Vashist P, Meel R, Grover S, Jain S, Kumar D, Gupta V, Tandon R, Solomon AW. Prospective audit of the phenotype, causes and correlates of trachomatous and non-trachomatous trichiasis in a peri-elimination setting. PLOS Neglected Tropical Diseases. 2022 Dec 27;16(12):e0011014.	Excluded	Excluded after title and abstract screening
347	Mboni C. *Outcome of penetrating Keratoplasties performed at Kikuyu Eye Unit from 1993 to 2003* (Doctoral dissertation, University of Nairobi).	Excluded	Excluded after title and abstract screening
348	Arunga S. *The Epidemiology of Microbial Keratitis in South Western Uganda* (Doctoral dissertation, London School of Hygiene & Tropical Medicine).	Excluded	Excluded after title and abstract screening
349	Nyamwaro CM. Prevalence, and Risk Factors for Trachoma Infection Among Children Aged 1–9 Years Old in Oldonyonyokie Location, Magadi Division, Kajiado County, Ethiopia	Excluded	Excluded after title and abstract screening
350	Whitcher JP, Srinivasan M, Upadhyay MP. Corneal blindness: a global perspective. Bulletin of the world health organization. 2001;79(3):214-21.	Excluded	Excluded after title and abstract screening
351	Woodward L. Pediatric Eye Disease in Tanzania. International Ophthalmology Clinics. 2010 Oct 1;50(4):137-48.	Excluded	Excluded after title and abstract screening
352	Vashist P, Meel R, Grover S, Jain S, Kumar D, Gupta V, Tandon R, Solomon AW. Prospective audit of the phenotype, causes and correlates of trachomatous and non-trachomatous trichiasis in a peri-elimination setting. PLOS Neglected Tropical Diseases. 2022 Dec 27;16(12):e0011014	Excluded	Excluded after title and abstract screening
353	Sivasubramaniam S, Rabiu MM, Kyari F, Solomon AW, Gilbert C. Multilevel analysis of trachomatous	Excluded	Excluded after title and abstract screening

	trichiasis and corneal opacity in Nigeria: the role of environmental and climatic risk factors on the distribution of disease. PLoS neglected tropical diseases. 2015 Jul 29;9(7):e0003826.		
354	Kamau JW. *Prevalence, Risk Factors and Trachoma Causing Species Circulating in East Pokot, Baringo County, Kenya* (Doctoral dissertation).	Excluded	Excluded after title and abstract screening
355	Astle WF, Wiafe B, Ingram AD, Mwanga M, Glassco CB. Trachoma control in Southern Zambia—an international team project employing the SAFE strategy. Ophthalmic epidemiology. 2006 Jan 1;13(4):227-36.	Excluded	Conducted outside the eligible geographic area (Ethiopia)
356	Mwale C, Mumbi W, Funjika M, Sokesi T, Silumesii A, Mulenga M, Mutati G, Kwendakwema D, Chelu C, Adamu Y, Alemayehu W. Prevalence of trachoma in 47 administrative districts of Zambia: results of 32 population-based prevalence surveys. Ophthalmic epidemiology. 2018 Dec 28;25(sup1):171-80.	Excluded	Conducted outside the eligible geographic area (Ethiopia)
357	Kilangalanga J, Ndjemba JM, Uvon PA, Kibangala FM, Mwandulo JL, Mavula N, Ndombe M, Kazadi J, Limbaka H, Cohn D, Tougoue JJ. Trachoma in the Democratic Republic of the Congo: results of 46 baseline prevalence surveys conducted with the Global Trachoma Mapping Project. Ophthalmic epidemiology. 2018 Dec 28;25(sup1):192-200.	Excluded	Conducted outside the eligible geographic area (Ethiopia)
358	Renneker KK, Abdala M, Addy J, Al-Khatib T, Amer K, Badiane MD, Batcho W, Bella L, Bougouma C, Bucumi V, Chisenga T. Global progress toward the elimination of active trachoma: an analysis of 38 countries. The Lancet Global Health. 2022 Apr 1;10(4):e491-500.	Excluded	Conducted outside the eligible geographic area (Ethiopia)
359	Chikwanda M, Mubita P, Munyinda N, Mwale C, Mbanefo P, Banda TC. Research Paper An association	Excluded	Conducted outside the eligible geographic area (Ethiopia)

	between water, sanitation, and hygiene (WASH) and prevalence of trachoma in Monze district of Southern Province, Zambia.		
360	Phiri I, Manangazira P, Macleod CK, Mduluza T, Dhobbie T, Chaora SG, Chigwena C, Katiyo J, Willis R, Bakhtiari A, Bare P. The burden of and risk factors for trachoma in selected districts of Zimbabwe: results of 16 population-based prevalence surveys. Ophthalmic epidemiology. 2018 Dec 28;25(sup1):181-91.	Excluded	Conducted outside the eligible geographic area (Ethiopia)
361	Rajak SN, Habtamu E, Weiss HA, Bedri A, Zerihun M, Gebre T, Gilbert CE, Emerson PM, Burton MJ. Why do people not attend for treatment for trachomatous trichiasis in Ethiopia? A study of barriers to surgery.	Excluded	Duplicated
362	Ngondi J, Gebre T, Shargie EB, Graves PM, Ejigsemahu Y, Teferi T, Genet A, Mosher AW, Endeshaw T, Zerihun M, Messele A. Risk factors for active trachoma in children and trichiasis in adults: a household survey in Amhara Regional State, Ethiopia. Transactions of the Royal Society of Tropical Medicine and Hygiene. 2008 May 1;102(5):432-8.	Excluded	Duplicated
363	Manavi K. A review on infection with Chlamydia trachomatis. Best Practice & Research Clinical Obstetrics & Gynaecology. 2006 Dec 1;20(6):941-51.	Excluded	Excluded due to the outcome of interest not reported
364	Brunham RC, Rey-Ladino J. Immunology of Chlamydia infection: implications for a Chlamydia trachomatis vaccine. Nature reviews immunology. 2005 Feb 1;5(2):149-61.	Excluded	Excluded due to the outcome of interest not reported
364	Stamm WE. Chlamydia trachomatis infections: progress and problems. The Journal of infectious diseases. 1999 Mar 1;179(Supplement_2):S380-3.	Excluded	Excluded due to the outcome of interest not reported

365	Wyrick PB. Chlamydia trachomatis persistence in vitro: an overview. The Journal of infectious diseases. 2010 Jun 15;201(Supplement_2):S88-95.	Excluded	Excluded due to the outcome of interest not reported
366	Miller KE. Diagnosis and treatment of Chlamydia trachomatis infection. American family physician. 2006 Apr 15;73(8):1411-6.	Excluded	Excluded due to the outcome of interest not reported
367	Witkin SS, Minis E, Athanasiou A, Leizer J, Linhares IM. Chlamydia trachomatis: the persistent pathogen. Clinical and Vaccine Immunology. 2017 Oct;24(10):e00203-17.	Excluded	Excluded due to the outcome of interest not reported
368	Carder C, Mercey D, Benn P. Chlamydia trachomatis. Sexually transmitted infections. 2006 Dec 1;82(suppl 4):iv10-2.	Excluded	Excluded due to the outcome of interest not reported
369	Schachter J, Grossman M, Sweet RL, Holt J, Jordan C, Bishop E. Prospective study of perinatal transmission of Chlamydia trachomatis. Jama. 1986 Jun 27;255(24):3374-7.	Excluded	Excluded due to the outcome of interest not reported
370	Read TD, Brunham RC, Shen C, Gill SR, Heidelberg JF, White O, Hickey EK, Peterson J, Utterback T, Berry K, Bass S. Genome sequences of Chlamydia trachomatis MoPn and Chlamydia pneumoniae AR39. Nucleic acids research. 2000 Mar 15;28(6):1397-406.	Excluded	Excluded due to the outcome of interest not reported
371	Clarke IN. Evolution of Chlamydia trachomatis. Annals of the New York Academy of Sciences. 2011 Aug;1230(1):E11-8.	Excluded	Excluded due to the outcome of interest not reported
372	Schachter J, Hill EC, King EB, Heilbron DC, Ray RM, Margolis AJ, Greenwood SA. Chlamydia trachomatis and cervical neoplasia. Jama. 1982 Nov 5;248(17):2134-8.	Excluded	Excluded due to the outcome of interest not reported
373	Baud D, Goy G, Jaton K, Osterheld MC, Blumer S, Borel N, Vial Y, Hohlfeld P, Pospischil A, Greub G. Role of Chlamydia trachomatis in miscarriage. Emerging infectious diseases. 2011 Sep;17(9):1630.	Excluded	Excluded due to the outcome of interest not reported

374	Land JA, Van Bergen JE, Morre SA, Postma MJ. Epidemiology of Chlamydia trachomatis infection in women and the cost-effectiveness of screening. Human reproduction update. 2010 Mar 1;16(2):189-204.	Excluded	Excluded due to the outcome of interest not reported
375	Habtamu E, Weiss HA, Bedri A, Gebre T, Genet A, Khaw PT, Bailey RL, Mabey DC, Gilbert CE, Emerson PM. Epilation for trachomatous trichiasis and the risk of corneal opacification. Ophthalmology. 2012 Jan 1;119(1):84	Excluded	Excluded after title and abstract screening
376	Bejiga A. Common eye diseases in children of rural community in Goro district, Central Ethiopia. Ethiopian Journal of Health Development. 2005 Oct 27;19(2):148-52	Excluded	Excluded after title and abstract screening
377	Senbete GH, Bogale GG. Determinants for not utilizing trachomatous trichiasis surgery among trachomatous trichiasis patients in Mehalsayint District, North-East Ethiopia. PLoS neglected tropical diseases. 2018 Jul 18;12(7):e0006669.	Excluded	Excluded after title and abstract screening
378	Wondie T, Habtamu E, Deribe K, Rajak S, Bremner S, Davey G. Podoconiosis, trachomatous trichiasis and cataract in northern Ethiopia: A comparative cross sectional study. PLoS neglected tropical diseases. 2017 Feb 10;11(2):e0005388.	Excluded	Excluded after title and abstract screening
379	Asfaw MA, Zerdo Z. Knowledge and attitude of community towards trachoma and trichiasis in Arba Minch Zuria district, Gamo Zone, Southern Ethiopia, 2019.	Excluded	Excluded after title and abstract screening
380	, Bejiga A. Common eye diseases in children of rural community in Goro district, Central Ethiopia. Ethiopian Journal of Health Development. 2005 Oct 27;19(2):148-52.	Excluded	Excluded after title and abstract screening
381	Wosen A, Bekana F, Ayanos T. Prevalence of trachoma and associated risk factors among Yello	Excluded	Duplicated

	elementary school students. Loma Woreda, Dawro Zone, Ethiopia: J Nursing care; 2015.		
382	Sherief ST, Macleod C, Gigar G, Godefay H, Abraha A, Dejene M, Kello AB, Belete A, Assefa Y, Willis R, et al. The Prevalence of Trachoma in TigrayRegion, Northern Ethiopia: Results of 11 Population-Based Prevalence Surveys Completed as Part of the Global Trachoma Mapping Project. Ophthalmic Epidemiol. 2016;23(sup1):94–9	Excluded	Duplicated
383	Darville T. Chlamydia trachomatis infections in neonates and young children. InSeminars in pediatric infectious diseases 2005 Oct 1 (Vol. 16, No. 4, pp. 235-244). WB Saunders.	Excluded	Outcome of interest not reported
384	Hammerschlag MR. Chlamydia trachomatis and Chlamydia pneumoniae infections in children and adolescents. Pediatrics in review. 2004 Feb 1;25(2):43-51.	Excluded	Outcome of interest not reported
385	Bobo LD, Novak N, Muñoz B, Hsieh YH, Quinn TC, West S. Severe disease in children with trachoma is associated with persistent Chlamydia trachomatis infection. Journal of infectious diseases. 1997 Dec 1;176(6):1524-30.	Excluded	Outcome of interest not reported
386	Banniettis N, Thumu S, Weedon J, Chotikanatis K, Szigeti A, Hammerschlag MR, Kohlhoff SA. Seroprevalence of Chlamydia trachomatis in inner-city children and adolescents—Implications for vaccine development. Sexually Transmitted Diseases. 2017 Dec 1;44(12):717-21.	Excluded	Outcome of interest not reported
387	Webley WC, Tilahun Y, Lay K, Patel K, Stuart ES, Andrzejewski C, Salva PS. Occurrence of Chlamydia trachomatis and Chlamydia pneumoniae in paediatric respiratory infections. European Respiratory Journal. 2009 Feb 1;33(2):360-7.	Excluded	Outcome of interest not reported

388	Wolle MA, Muñoz BE, Mkocha H, West SK. Constant ocular infection with Chlamydia trachomatis predicts risk of scarring in children in Tanzania. Ophthalmology. 2009 Feb 1;116(2):243-7.	Excluded	Outcome of interest not reported
389	Grayston JT. Chlamydia pneumoniae (TWAR) infections in children. The Pediatric infectious disease journal. 1994 Aug 1;13(8):675-85.	Excluded	Outcome of interest not reported
390	Renneker KK, Abdala M, Addy J, Al-Khatib T, Amer K, Badiane MD, Batcho W, Bella L, Bougouma C, Bucumi V, Chisenga T. Global progress toward the elimination of active trachoma: an analysis of 38 countries. The Lancet Global Health. 2022 Apr 1;10(4):e491-500.	Excluded	Conducted outside the eligible geographic area (Ethiopia)
391	Smith JL, Flueckiger RM, Hooper PJ, Polack S, Cromwell EA, Palmer SL, Emerson PM, Mabey DC, Solomon AW, Haddad D, Brooker SJ. The geographical distribution and burden of trachoma in Africa. PLoS neglected tropical diseases. 2013 Aug 8;7(8):e2359.	Excluded	Conducted outside the eligible geographic area (Ethiopia)
392	Ballard RC, Fehler HG, Fotheringham P, Sutter EE, Treharne JD. Trachoma in South Africa. Social Science & Medicine. 1983 Jan 1;17(22):1755-65.	Excluded	Conducted outside the eligible geographic area (Ethiopia)
393	Burton MJ. Trachoma: an overview. British medical bulletin. 2007 Dec 1;84(1):99-116.	Excluded	Conducted outside the eligible geographic area (Ethiopia)
394	Kalua K, Chirwa T, Kalilani L, Abbenyi S, Mukaka M, Bailey R. Prevalence and risk factors for trachoma in central and southern Malawi. PLoS One. 2010 Feb 5;5(2):e9067.	Excluded	Conducted outside the eligible geographic area (Ethiopia)
395	Kok PW. The epidemiology of trachoma blindness in southern Africa. Social Science & Medicine. 1983 Jan 1;17(22):1709-13.	Excluded	Conducted outside the eligible geographic area (Ethiopia)

396	Sutter EE, Ballard RC. Community participation in the control of trachoma in Gazankulu. Social science & medicine. 1983 Jan 1;17(22):1813-7.	Excluded	Conducted outside the eligible geographic area (Ethiopia)
397	Kalilani L, Abbenyi S, Mukaka M, Bailey R. Prevalence and risk factors for trachoma in central and southern Malawi. PLoS One. 2010 Feb 5;5(2):e9067.	Excluded	Conducted outside the eligible geographic area (Ethiopia)
398	Tadesse B, Worku A, Kumie A, Yimer SA. The burden of and risk factors for active trachoma in the north and south Wollo zones of Amhara region, Ethiopia: a cross-sectional study. *Infect Dis Poverty*. 2017;**6**(1):143. doi: 10.1186/s40249-017-0358-3.	Excluded	Duplicated
399	Mehari ZA. Pattern of childhood ocular morbidity in rural eye hospital Central Ethiopia. BMC Ophthalmol. 2014;14(1):50.	Excluded	Duplicated
400	al Arab GE, Tawfik N, El Gendy R, Anwar W, Courtright P. The burden of trachoma in the rural Nile Delta of Egypt: a survey of Menofiya governorate. British journal of ophthalmology. 2001 Dec 1;85(12):1406-10.	Excluded	Conducted outside the eligible geographic area (Ethiopia)
401	Elarab GE, Khan M. Estimation of the prevalence of trachoma in Egypt. British Journal of Ophthalmology. 2010 Mar 1;94(3):392-.	Excluded	Conducted outside the eligible geographic area (Ethiopia)
402	Kwami CS, Godfrey S, Gavilan H, Lakhanpaul M, Parikh P. Water, sanitation, and hygiene: linkages with stunting in rural Ethiopia. International journal of environmental research and public health. 2019 Oct;16(20):3793.	Excluded	Excluded after title and abstract screening
403	Berhe AA, Aregay AD, Abreha AA, Aregay AB, Gebretsadik AW, Negash DZ, Gebreegziabher EG, Demoz KG, Fenta KA, Mamo NB. Knowledge, attitude, and practices on water, sanitation, and hygiene among rural residents in Tigray Region,	Excluded	Excluded after title and abstract screening

	Northern Ethiopia. Journal of Environmental and Public Health. 2020;2020(1):5460168.		
404	Girma M, Hussein A, Norris T, Genye T, Tessema M, Bossuyt A, Hadis M, van Zyl C, Goyol K, Samuel A. Progress in water, sanitation and hygiene (WASH) coverage and potential contribution to the decline in diarrhea and stunting in Ethiopia. Maternal & Child Nutrition. 2024 Jul;20:e13280.	Excluded	Excluded after title and abstract screening
405	Amer K, Müller A, Abdelhafiz HM, Al-Khatib T, Bakhtiari A, Boisson S, El Arab GE, Gad H, Gordon BA, Madian A, Fahmi AT. Prevalence of trachoma in four marakez of Elmenia and Bani Suef Governorates, Egypt. Ophthalmic epidemiology. 2018 Dec 28;25(sup1):70-8.	Excluded	Conducted outside the eligible geographic area (Ethiopia)
406	Nayel Y, Taylor M, Montasser AS, Elsherif M, Diab MM. Perceptions of ophthalmologists on the impact of trachoma in Egypt: a mixed-methods, nationwide survey. BMC Infectious Diseases. 2023 Jan 17;23(1):27.	Excluded	Conducted outside the eligible geographic area (Ethiopia)
407	Hammou J, El Ajaroumi H, Hasbi H, Nakhlaoui A, Hmadna A, El Maaroufi A. In Morocco, the elimination of trachoma as a public health problem becomes a reality. The Lancet Global Health. 2017 Mar 1;5(3):e250-1.	Excluded	Conducted outside the eligible geographic area (Ethiopia)
408	Ferede AT, Dadi AF, Tariku A, Adane AA. Prevalence and determinants of active trachoma among preschool-aged children in Dembia District Northwest Ethiopia. *Infect Dis Poverty*. 2017;**6**(1):128. doi: 10.1186/s40249-017-0345-8.	Excluded	Duplicated
409	Ketema K, Tiruneh M, Woldeyohannes D, Muluye D. Active trachoma and associated risk factors among children in Baso Liben District of East Gojjam	Excluded	Duplicated

	Ethiopia. *BMC Public Health.* 2012;**12**(1):1105. doi: 10.1186/1471-2458-12-1105.		
410	Emerson PM, Ngondi J, Biru E, Graves PM, Ejigsemahu Y, Gebre T, Endeshaw T, Genet A, Mosher AW, Zerihun M. Integrating an NTD with one of “the big three”: combined malaria and trachoma survey in Amhara region of Ethiopia. PLoS Negl Trop Dis. 2008;2(3):e197	Excluded	Duplicated
411	Grimes JE, Tadesse G, Mekete K, Wuletaw Y, Gebretsadik A, French MD, Harrison WE, Drake LJ, Gardiner IA, Yard E, Templeton MR. School water, sanitation, and hygiene, soil-transmitted helminths, and schistosomes: national mapping in Ethiopia. PLoS neglected tropical diseases. 2016 Mar 8;10(3):e0004515.	Excluded	Excluded after title and abstract screening
412	Anteneh ZA, Getu WY. Prevalence of active trachoma and associated risk factors among children in Gazegibela district of Wagehemra Zone, Amhara region, Ethiopia: community-based cross-sectional study. *Trop Dis Travel Med Vaccines.* 2016;**2**(1):5. doi: 10.1186/s40794-016-0022-0	Excluded	Duplicated
413	Assefa GM, Sherif S, Sluijs J, Kuijpers M, Chaka T, Solomon A, Hailu Y, Muluneh MD. Gender equality and social inclusion in relation to water, sanitation and hygiene in the Oromia region of Ethiopia. International Journal of Environmental Research and Public Health. 2021 Apr 17;18(8):4281.	Excluded	Excluded after title and abstract screening
414	Admasie A, Debebe A. Estimating access to drinking water supply, sanitation, and hygiene facilities in Wolaita Sodo town, southern Ethiopia, in reference to national coverage. Journal of Environmental and Public Health. 2016;2016(1):8141658.	Excluded	Excluded after title and abstract screening

415	Karmaoui A, El Jaafari S, Chaachouay H, Hajji L. Socio-ecological factors influencing vulnerability to trachoma disease: a new tool applied in five pre-saharan provinces, Morocco. GeoJournal. 2023 Jun;88(3):2669-90.	Excluded	Conducted outside the eligible geographic area (Ethiopia)
416	Ketema K, Tiruneh M, Woldeyohannes D, Muluye D. Active trachoma and associated risk factors among children in Baso Liben District of East Gojjam Ethiopia. *BMC Public Health.* 2012;**12**(1):1105. doi: 10.1186/1471-2458-12-1105.	Excluded	Duplicated
417	Phiri I, Manangazira P, Macleod CK, Mduluza T, Dhobbie T, Chaora SG, Chigwena C, Katiyo J, Willis R, Bakhtiari A, Bare P. The burden of and risk factors for trachoma in selected districts of Zimbabwe: results of 16 population-based prevalence surveys. Ophthalmic epidemiology. 2018 Dec 28;25(sup1):181-91.	Excluded	Conducted outside the eligible geographic area (Ethiopia)
418	Ngondi J, Matthews F, Reacher M, Onsarigo A, Matende I, Baba S, Brayne C, Zingeser J, Emerson P. Prevalence of risk factors and severity of active trachoma in southern Sudan: an ordinal analysis. American Journal of Tropical Medicine and Hygiene. 2007 Jul 1;77(1):126-32.	Excluded	Conducted outside the eligible geographic area (Ethiopia)
419	Clements AC, Kur LW, Gatpan G, Ngondi JM, Emerson PM, Lado M, Sabasio A, Kolaczinski JH. Targeting trachoma control through risk mapping: the example of Southern Sudan. PLoS Neglected Tropical Diseases. 2010 Aug 17;4(8):e799.	Excluded	Conducted outside the eligible geographic area (Ethiopia)
420	Ngondi J, Ole-Sempele F, Onsarigo A, Matende I, Baba S, Reacher M, Matthews F, Brayne C, Emerson P. Blinding trachoma in postconflict southern Sudan. PLoS Medicine. 2006 Dec;3(12):e478.	Excluded	Conducted outside the eligible geographic area (Ethiopia)

421	Nigusie A, Berhe R, Gedefaw M. Prevalence and associated factors of active trachoma among childeren aged 1–9 years in rural communities of Gonji Kolella district, West Gojjam zone North West Ethiopia. *BMC Res Notes.* 2015;**8**(1):641. doi: 10.1186/s13104-015-1529-6.	Excluded	Duplicated
422	Alemayehu W, Cherinet A. Eye diseases and Blindness. InThe Ecology Of Health And Disease In Ethiopia 2019 Jul 11 (pp. 237-250). Routledge.	Excluded	Excluded due to the outcome of interest not reported
423	Nigusie A, Berhe R, Gedefaw M. Prevalence and associated factors of active trachoma among childeren aged 1–9 years in rural communities of Gonji Kolella district, West Gojjam zone North West Ethiopia. *BMC Res Notes.* 2015;**8**(1):641. doi: 10.1186/s13104-015-1529-6.	Excluded	Duplicated
424	Stocks ME, Ogden S, Haddad D, Addiss DG, McGuire C. Effect of Water. Sanitation, and Hygiene on the Prevention of Trachoma: A. 2014.	Excluded	Excluded due to the outcome of interest not reported
425	Salim AR, Sheikh HA. Trachoma in the Sudan. An epidemiological study. British journal of ophthalmology. 1975 Oct 1;59(10):600-4.	Excluded	Conducted outside the eligible geographic area (Ethiopia)
426	Burn H, Aweke S, Wondie T, Habtamu E, Deribe K, Rajak S, Bremner S, Davey G. Podoconiosis, trachomatous trichiasis and cataract in northern Ethiopia: A comparative cross sectional study. PLoS neglected tropical diseases. 2017 Feb 10;11(2):e0005388.	Excluded	Excluded due to the outcome of interest not reported
427	Shiferaw D, Moges HG. Risk factors for active trachoma among children aged 1-9 years in Maksegnit town, Gondar Zuria District Northwest Ethiopia. *Risk.* 2013;**2**(3):202–206	Excluded	Duplicated
428	Worku Y, Bayu S. Screening for ocular abnormalities and subnormal vision in school children of Butajira	Excluded	Excluded due to the outcome of interest not reported

	Town, southern Ethiopia. Ethiopian Journal of Health Development. 2002;16(2):165-71.		
429	Golovaty I, Jones L, Gelaye B, Tilahun M, Belete H, Kumie A, Berhane Y, Williams MA. Access to water source, latrine facilities and other risk factors of active trachoma in Ankober Ethiopia. *PLoS One*. 2009;**4**(8):e6702. doi: 10.1371/journal.pone.0006702	Excluded	Duplicated
430	Ngondi J, Ole-Sempele F, Onsarigo A, Matende I, Baba S, Reacher M, Matthews F, Brayne C, Emerson P. Blinding trachoma in postconflict southern Sudan. PLoS Medicine. 2006 Dec;3(12):e478.	Excluded	Conducted outside the eligible geographic area (Ethiopia)
431	Rajak SN, Habtamu E, Weiss HA, Kello AB, Abera B, Zerihun M, Gebre T, Gilbert CE, Khaw PT, Emerson PM, Burton MJ. The outcome of trachomatous trichiasis surgery in Ethiopia: risk factors for recurrence. PLoS neglected tropical diseases. 2013 Aug 22;7(8):e2392.	Excluded	Excluded after title and abstract screening
432	Churko C, Asfaw MA, Zerdo Z. Knowledge, attitude, practices and associated factors towards trachoma among people living in Arba Minch Zuria District, Gamo Zone, Southern Ethiopia. Clinical Ophthalmology. 2021 Jul 16:3075-85.	Excluded	Excluded due to the outcome of interest not reported
433	Habtamu E, Wondie T, Aweke S, Tadesse Z, Zerihun M, Melak B, Gashaw B, Callahan K, Emerson PM, Bailey RL, Mabey DC. Impact of trichiasis surgery on daily living: a longitudinal study in Ethiopia. Wellcome open research. 2017;2.	Excluded	Excluded after title and abstract screening
434	Rajak SN, Habtamu E, Weiss HA, Bedri A, Zerihun M, Gebre T, Gilbert CE, Emerson PM, Burton MJ. Why do people not attend for treatment for trachomatous trichiasis in Ethiopia? A study of barriers to surgery.	Excluded	Excluded after title and abstract screening

435	Cumberland P, Edwards T, Hailu G, Harding-Esch E, Andreasen A, Mabey D, Todd J. The impact of community level treatment and preventative interventions on trachoma prevalence in rural Ethiopia. International journal of epidemiology. 2008 Jun 1;37(3):549-58.	Excluded	Excluded due to the outcome of interest not reported
436	Churko C, Asfaw MA, Zerdo Z. Exploring barriers for trachomatous trichiasis surgery implementation in gamo zone, Southern Ethiopia. PLoS Neglected Tropical Diseases. 2021 Sep 15;15(9):e0009780.	Excluded	Excluded after title and abstract screening
437	Habtamu E, Rajak SN, Gebre T, Zerihun M, Genet A, Emerson PM, Burton MJ. Clearing the backlog: trichiasis surgeon retention and productivity in Northern Ethiopia. PLoS neglected tropical diseases. 2011 Apr 5;5(4):e1014.	Excluded	Excluded after title and abstract screening
438	Gower EW, Munoz B, Rajak S, Habtamu E, West SK, Merbs SL, Harding JC, Alemayehu W, Callahan EK, Emerson PM, Gebre T. Pre-operative trichiatic eyelash pattern predicts post-operative trachomatous trichiasis. PLoS neglected tropical diseases. 2019 Oct 7;13(10):e0007637.	Excluded	Excluded after title and abstract screening
439	Tadesse F. Prevalence of Trachoma After Three Rounds of Antibiotic Mass Drug Administration in 13 Woredas of Gambella Region, Ethiopia. Ophthalmic epidemiology. 2023 Dec 2:1-9.	Excluded	Excluded after title and abstract screening
440	Alemayehu A, Mekonen A, Mengistu B, Mihret A, Asmare A, Bakhtiari A, Mengistu B, Jimenez C, Kebede D, Bol D, Tadesse F. Prevalence of Trachoma After Three Rounds of Antibiotic Mass Drug Administration in 13 Woredas of Gambella Region, Ethiopia. Ophthalmic epidemiology. 2023 Dec 2:1-9.	Excluded	Excluded after title and abstract screening
441	Tadesse B, Worku A, Kumie A, Yimer SA. Effect of water, sanitation and hygiene interventions on active	Excluded	Non observational study design

	trachoma in North and South Wollo zones of Amhara Region, Ethiopia: A Quasi-experimental study. PLoS neglected tropical diseases. 2017 Nov 10;11(11):e0006080.		
442	Burton MJ, Rajak SN, Hu VH, Ramadhani A, Habtamu E, Massae P, Tadesse Z, Callahan K, Emerson PM, Khaw PT, Jeffries D. Pathogenesis of progressive scarring trachoma in Ethiopia and Tanzania and its implications for disease. PLoS neglected tropical diseases. 2015 May 13;9(5):e0003763.	Excluded	Non observational study design
443	Rajak SN, Habtamu E, Weiss HA, Kello AB, Gebre T, Genet A, Bailey RL, Mabey DC, Khaw PT, Gilbert CE, Emerson PM. Surgery versus epilation for the treatment of minor trichiasis in Ethiopia: a randomised controlled noninferiority trial. PLoS medicine. 2011 Dec 13;8(12):e1001136.	Excluded	Non observational study design
444	Rajak SN, Habtamu E, Weiss HA, Kello AB, Gebre T, Genet A, Bailey RL, Mabey DC, Khaw PT, Gilbert CE, Emerson PM. Absorbable versus silk sutures for surgical treatment of trachomatous trichiasis in Ethiopia: a randomised controlled trial. PLoS medicine. 2011 Dec 13;8(12):e1001137.	Excluded	Non observational study design
445	Ejigu M, Kariuki MM, Ilako DR, Gelaw Y. Rapid trachoma assessment in kersa district, Southwest Ethiopia. Ethiop J Health Sci. 2013;23(1):1–9	Excluded	duplicated
446	Habtamu E, FRCOPHTH SR, MA MJ. Surgical management strategies for trachomatous trichiasis.	Excluded	Non observational study design
447	Mehari ZA. Pattern of childhood ocular morbidity in rural eye hospital Central Ethiopia. BMC Ophthalmol. 2014;14(1):50	Excluded	Duplicated
448	Gupta N, Vashist P, Senjam SS, Gupta V, Wadhwani M, Manna S, Grover S, Bhardwaj A. Current status of	Excluded	Conducted outside the eligible geographic area (Ethiopia)

	trachoma in India: results from the National Trachoma Prevalence Survey. Indian Journal of Ophthalmology. 2022 Sep 1;70(9):3260-5.		
449	Bero B, Macleod C, Alemayehu W, Gadisa S, Abajobir A, Adamu Y, Alemu M, Adamu L, Dejene M, Mekasha A. Prevalence of and risk factors for trachoma in Oromia regional state of Ethiopia: results of 79 population-based prevalence surveys conducted with the global trachoma mapping project. Ophthalmic Epidemiol. 2016;23(6):392–	Excluded	Duplicated
450	Khanduja S, Jhanji V, Sharma N, Vashist P, Murthy GV, Gupta SK, Satpathy G, Tandon R, Titiyal JS, Vajpayee RB. Trachoma prevalence in women living in rural northern India: rapid assessment findings. Ophthalmic epidemiology. 2012 Aug 1;19(4):216-20.	Excluded	Conducted outside the eligible geographic area (Ethiopia)
451	Wosen A, Bekana F, Ayanos T. Prevalence of trachoma and associated risk factors among Yello elementary school students. Loma Woreda, Dawro Zone, Ethiopia: J Nursing care; 2015.	Excluded	Excluded due to publication period falling before the pre-specified time frame
452	Mengistu K, Shegaze M, Woldemichael K, Gesesew H, Markos Y. Prevalence and factors associated with trachoma among children aged 1–9 years in Zala district, gamo gofa Zone, southern ethiopia. Clin Ophthalmol (Auckland, NZ). 2016;10:1663	Excluded	Excluded due to publication period falling before the pre-specified time frame
453	Bejiga A. Common eye diseases in children of rural community in Goro district, Central Ethiopia. Ethiop J Health Dev. 2005;19(2):148–52.	Excluded	Excluded due to publication period falling before the pre-specified time frame
454	Negussu Ayele N, Macleod CK, Kello AB, Eshetu Gezachew Z, Binegdie A, Dejene M, Alemayehu W, Flueckiger RM, Massae PA. Epidemiology of trachoma and its implications for implementing the “SAFE” strategy in Somali Region, Ethiopia: results of	Excluded	Excluded due to publication period falling before the pre-specified time frame

	14 population-based prevalence surveys. Ophthalmic Epidemiol. 2018;25(sup1):25–32.		
455	Melese M, Fredlander E, Worku A, Courtright P. Active trachoma in children in Central Ethiopia: association with altitude. Trans R Soc Trop Med Hyg. 2005;99(11):840–3.	Excluded	Excluded due to publication period falling before the pre-specified time frame
456	Bejiga A, Alemayehu W. Prevalence of trachoma and its determinants in Dalocha District Central. Ethiopia Ophthalmic Epidemiol. 2001;8(2–3):119–25	Excluded	Excluded due to publication period falling before the pre-specified time frame
457	Gebre T, Shargie EB, Graves PM, Ejigsemahu Y, Teferi T, Genet A, Mosher AW, Endeshaw T, Zerihun M. Risk factors for active trachoma in children and trichiasis in adults: a household survey in Amhara regional state, Ethiopia. Trans R Soc Trop Med Hyg. 2008;102(5):432–8.	Excluded	Excluded due to publication period falling before the pre-specified time frame
458	Alemayehu W. Prevalence of trachoma and its determinants in Dalocha District Central. Ethiopia Ophthalmic Epidemiol. 2001;8(2–3):119–25	Excluded	Duplicated
459	Shargie EB, Graves PM, Ejigsemahu Y, Teferi T, Genet A, Mosher AW, Endeshaw T, Zerihun M. Risk factors for active trachoma in children and trichiasis in adults: a household survey in Amhara regional state, Ethiopia. Trans R Soc Trop Med Hyg. 2008;102(5):432–8.	Excluded	duplicated
460	Gebre T, Shargie EB, Graves PM, Ejigsemahu Y, Teferi T, Genet A, Mosher AW, Endeshaw T, Zerihun M. Risk factors for active trachoma in children and trichiasis in adults: a household survey in Amhara regional state, Ethiopia. Trans R Soc Trop Med Hyg. 2008;102(5):432–8.	Excluded	Duplicated
461	Roba AA, Wondimu A, Patel D, Zondervan M. Effects of intervention with the SAFE strategy on trachoma	Excluded	Excluded due to the outcome of interest not reported

	across Ethiopia. Journal of Epidemiology & Community Health. 2011 Jul 1;65(7):626-31.		
462	Burssa D, Teshome A, Iverson K, Ahearn O, Ashengo T, Barash D, Barringer E, Citron I, Garringer K, McKitrick V, Meara J. Safe surgery for all: early lessons from implementing a national government-driven surgical plan in Ethiopia. World journal of surgery. 2017 Dec;41(12):3038-45.	Excluded	Excluded due to the outcome of interest not reported
463	Bejiga A, Alemayehu W. Prevalence of trachoma and its determinants in Dalocha District Central. Ethiopia Ophthalmic Epidemiol. 2001;8(2–3):119–25	Excluded	Duplicated
464	Ngondi J, Gebre T, Shargie EB, Graves PM, Ejigsemahu Y, Teferi T, Genet A, Mosher AW, Endeshaw T, Zerihun M. Risk factors for active trachoma in children and trichiasis in adults: a household survey in Amhara regional state, Ethiopia. Trans R Soc Trop Med Hyg. 2008;102(5):432–8.	Excluded	duplicated
465	Habtamu E, FRCOPHTH SR, MA MJ. Surgical management strategies for trachomatous trichiasis.	Excluded	Excluded due to the outcome of interest not reported
466	Derrick T, Luthert PJ, Jama H, Hu VH, Massae P, Essex D, Holland MJ, Burton MJ. Increased epithelial expression of CTGF and S100A7 with elevated subepithelial expression of IL-1β in trachomatous trichiasis. PLoS neglected tropical diseases. 2016 Jun 1;10(6):e0004752.	Excluded	Excluded due to the outcome of interest not reported
467	Gupta N, Vashist P, Tandon R, Gupta SK, Dwivedi S, Mani K. Prevalence of corneal diseases in the rural Indian population: the Corneal Opacity Rural Epidemiological (CORE) study. British Journal of Ophthalmology. 2015 Feb 1;99(2):147-52.	Excluded	Excluded due to the outcome of interest not reported
468	Gupta N, Vashist P, Tandon R, Gupta SK, Dwivedi S, Mani K. Prevalence of corneal diseases in the rural Indian population: the Corneal Opacity Rural	Excluded	Excluded due to the outcome of interest not reported

	Epidemiological (CORE) study. British Journal of Ophthalmology. 2015 Feb 1;99(2):147-52.		
469	Bejiga A, Alemayehu W. Prevalence of trachoma and its determinants in Dalocha District Central. Ethiopia Ophthalmic Epidemiol. 2001;8(2–3):119–25	Excluded	Duplicated
470	Ferede AT, Alemu DS, Gudeta AD, Alemu HW, Melese MA. Visual impairment among primary school children in Gondar town, Northwest Ethiopia. Journal of ophthalmology. 2020;2020(1):6934013.	Excluded	Excluded after title and abstract screening
471	Habtamu E, Wondie T, Aweke S, Tadesse Z, Zerihun M, Zewdie Z, Callahan K, Emerson PM, Kuper H, Bailey RL. Trachoma and relative poverty: a casecontrol study. PLoS Negl Trop Dis. 2015;9(11):e0004228	Excluded	Duplicated
472	Zelalem M, Abebe Y, Adamu Y, Getinet T. Prevalence of visual impairment among school children in three primary schools of Sekela Woreda, Amhara regional state, north-west Ethiopia. SAGE open medicine. 2019 May;7:2050312119849769.	Excluded	Excluded after title and abstract screening
473	Shargie EB, Graves PM, Ejigsemahu Y, Teferi T, Genet A, Mosher AW, Endeshaw T, Zerihun M. Risk factors for active trachoma in children and trichiasis in adults: a household survey in Amhara regional state, Ethiopia. Trans R Soc Trop Med Hyg. 2008;102(5):432–8	Excluded	Duplicated
474	Tabor Yimam A, Wassie GT, Alene GD. Postoperative trachomatous trichiasis and associated factors among adults who underwent trachomatous trichiasis surgery in Ambassel District, North-East Ethiopia. Plos one. 2024 May 28;19(5):e0304407.	Excluded	Excluded after title and abstract screening
475	Weiss HA, Bedri A, Gebre T, Bailey RL, Mabey DC, Khaw PT, Gilbert CE, Emerson PM, Burton MJ. The clinical phenotype of trachomatous trichiasis in	Excluded	Excluded after title and abstract screening

	Ethiopia: not all trichiasis is due to entropion. Investigative ophthalmology & visual science. 2011 Oct 1;52(11):7974-80		
476	Antwi-Adjei EK. Relationship between the prevalence of trachomatous inflammation in children (age 1-9years) and the prevalence of trichiasis in adults (age 15years and above) at a presumed steady state.	Excluded	Excluded after title and abstract screening
477	Anteneh ZA, Getu WY. Prevalence of active trachoma and associated risk factors among children in Gazegibela district of Wagehemra Zone, Amhara region, Ethiopia: community-based cross-sectional study. *Trop Dis Travel Med Vaccines.* 2016;**2**(1):5. doi: 10.1186/s40794-016-0022-0	Excluded	Duplicated
478	Adamu Y, Macleod C, Adamu L, Fikru W, Kidu B, Abashawl A, Dejene M, Chu BK, Flueckiger RM, Willis R, et al. Prevalence of Trachoma in Benishangul Gumuz Region, Ethiopia: Results of Seven Population-Based Surveys from the Global Trachoma Mapping Project. Ophthalmic Epidemiol. 2016;23(sup1):70–6	Excluded	Excluded due to publication period falling before the pre-specified time frame
479	Mehari ZA. Pattern of childhood ocular morbidity in rural eye hospital Central Ethiopia. BMC Ophthalmol. 2014;14(1):50.	Excluded	Excluded due to publication period falling before the pre-specified time frame
480	Kariuki MM, Ilako DR, Gelaw Y. Rapid trachoma assessment in kersa district, Southwest Ethiopia. Ethiop J Health Sci. 2013;23(1):1–9	Excluded	Excluded due to publication period falling before the pre-specified time frame
481	Gilbert CE, Anderton L, Dandona L, Foster A. Prevalence of visual impairment in children: a review of available data. Ophthalmic epidemiology. 1999 Jan 1;6(1):73-82.	Excluded	Excluded after title and abstract screening
482	Karimurio J, Ilako F, Gichangi M. Prevalence of active and potentially blinding trachoma in Laikipia district, Kenya.	Excluded	Conducted outside the eligible geographic area (Ethiopia)

483	Worku A, Kumie A, Yimer SA. The burden of and risk factors for active trachoma in the north and south Wollo zones of Amhara region, Ethiopia: a cross-sectional study. *Infect Dis Poverty*. 2017;**6**(1):143. doi: 10.1186/s40249-017-0358-3.	Excluded	Excluded due to publication period falling before the pre-specified time frame
484	Makau GN. *The Prevalence And Pattern Of Trachoma In Meru North District Kenya* (Doctoral dissertation).	Excluded	Conducted outside the eligible geographic area (Ethiopia)
485	Aragie S, Wittberg DM, Tadesse W, Dagnew A, Hailu D, Chernet A, Melo JS, Aiemjoy K, Haile M, Zeru T, Tadesse Z. Water, sanitation, and hygiene for control of trachoma in Ethiopia (WUHA): a two-arm, parallel-group, cluster-randomised trial. The Lancet Global Health. 2022 Jan 1;10(1):e87-95.	Excluded	Duplicated
486	Shah M, Khan M, Khan MT, Khan MY, Saeed N. Causes of visual impairment in children with low vision. J Coll Physicians Surg Pak. 2011 Feb 1;21(2):88-92.	Excluded	Excluded due to the outcome of interest not reported
487	Haddath MA, Sei M, Sampaio MW, Kara-José N. Causes of visual impairment in children: a study of 3,210 cases. Journal of pediatric ophthalmology and strabismus. 2007 Jul 1;44(4):232.	Excluded	Excluded due to the outcome of interest not reported
488	Nasieku L, Mutai J, Muthami L, Karanja S. Determinants of active trachoma among children aged 1-9 years in Ol Donyo Nyokie location, Kajiado County, Kenya. African Journal of Health Sciences. 2017;30(2):77-86.	Excluded	Conducted outside the eligible geographic area (Ethiopia)
489	Kumie A, Yimer SA. Effect of water, sanitation and hygiene interventions on active trachoma in North and South Wollo zones of Amhara Region, Ethiopia: A Quasi-experimental study. PLoS neglected tropical diseases. 2017 Nov 10;11(11):e0006080.	Excluded	Excluded due to inappropriate study design (non-observational)
490	Tadesse B, Worku A, Kumie A, Yimer SA. The burden of and risk factors for active trachoma in the	Excluded	Duplicated

	north and south Wollo zones of Amhara region, Ethiopia: a cross-sectional study. *Infect Dis Poverty*. 2017;**6**(1):143. doi: 10.1186/s40249-017-0358-3.		
491	Congdon N, West S, Vitale S, Katala S, Mmbaga BB. Exposure to children and risk of active trachoma in Tanzanian women. American journal of epidemiology. 1993 Feb 1;137(3):366-72.	Excluded	Conducted outside the eligible geographic area (Ethiopia)
492	Woldeamanuel GG, Biru MD, Geta TG, Areru BA. Visual impairment and associated factors among primary school children in Gurage Zone, Southern Ethiopia. African Health Sciences. 2020 Apr 20;20(1):533-42.	Excluded	Excluded due to the outcome of interest not reported
493	Anteneh ZA, Getu WY. Prevalence of active trachoma and associated risk factors among children in Gazegibela district of Wagehemra Zone, Amhara region, Ethiopia: community-based cross-sectional study. *Trop Dis Travel Med Vaccines.* ;**2**(1):5. doi: 10.1186/s40794-016-0022-0	Excluded	Duplicated
494	Maru L, Yitayew M, Getu D, Melkamu S, Debebe W. Visual impairment and associated factors among randomly selected secondary school students of Assosa zone, north west Ethiopia. Res Square. 2022 Oct 10:1-7.	Excluded	Excluded due to the outcome of interest not reported
495	Dawson CR, Daghfous T, Messadi M, Hoshiwara I, Schachter J. Severe endemic trachoma in Tunisia. British journal of ophthalmology. 1976 Apr 1;60(4):245-52.	Excluded	Conducted outside the eligible geographic area (Ethiopia)
496	Melkamu S, Debebe W. Visual impairment and associated factors among randomly selected secondary school students of Assosa zone, north west Ethiopia. Res Square. 2022 Oct 10:1-7.	Excluded	Duplicated

497	West ES, Munoz B, Imeru A, Alemayehu W, Melese M, West SK. The association between epilation and corneal opacity among eyes with trachomatous trichiasis. British journal of ophthalmology. 2006 Feb 1;90(2):171-4.	Excluded	Excluded due to the outcome of interest not reported
498	Hailu G, Todd J. Active trachoma in children aged three to nine years in rural communities in Ethiopia: prevalence, indicators and risk factors. Transactions of the Royal society of Tropical Medicine and Hygiene. 2005 Feb 1;99(2):120-7.	Excluded	Excluded due to publication period falling before the pre-specified time frame
499	Wright HR, Keeffe JE, Taylor HR. Elimination of trachoma: are we in danger of being blinded by the randomised controlled trial?. British journal of ophthalmology. 2006 Nov 1;90(11):1339-42.	Excluded	Non observational study design
500	Yalew KN, Mekonnen MG, Jemaneh AA. Trachoma and its determinants in Mojo and Lume districts of Ethiopia. The Pan African Medical Journal. 2012;13(Suppl 1).	Excluded	Duplicated
501	Rajak SN, Habtamu E, Weiss HA, Kello AB, Gebre T, Genet A, Bailey RL, Mabey DC, Khaw PT, Gilbert CE, Emerson PM. Absorbable versus silk sutures for surgical treatment of trachomatous trichiasis in Ethiopia: a randomised controlled trial. PLoS medicine. 2011 Dec 13;8(12):e1001137.	Excluded	Non observational study design
502	Reda G, Yemane D, Gebreyesus A. Prevalence and associated factors of active trachoma among 1–9 years old children in Deguatemben, Tigray, Ethiopia, 2018: community cross-sectional study. BMC ophthalmology. 2020 Dec;20:1-9.	Excluded	Excluded due to publication period falling before the pre-specified time frame
503	Polack S, Kuper H, Solomon AW, Massae PA, Abuelo C, Cameron E, Valdmanis V, Mahande M, Foster A, Mabey D. The relationship between prevalence of active trachoma, water availability and its use in a	Excluded	Conducted outside the eligible geographic area (Ethiopia)

	Tanzanian village. Transactions of the Royal Society of Tropical Medicine and Hygiene. 2006 Nov 1;100(11):1075-83.		
504	Macleod C, Adamu L, Fikru W, Kidu B, Abashawl A, Dejene M, Chu BK, Flueckiger RM, Willis R, Pavluck AL. Prevalence of trachoma in Benishangul Gumuz Region, Ethiopia: results of seven population-based surveys from the global trachoma mapping project. Ophthalmic epidemiology. 2016 Dec 7;23(sup1):70-6.	Excluded	Excluded due to publication period falling before the pre-specified time frame
