# Supplementary material for: Beyond the SAFE strategy: Systematic review and meta-analysis of prevalence and associated factors of active trachoma among children in Ethiopia
Source: PLoS One. 2025 Feb 20;20(2):e0312024. doi: 10.1371/journal.pone.0312024 (PMC11841906; doi:10.1371/journal.pone.0312024)
Supplement: S2 Table — (PDF) [file pone.0312024.s004.pdf]

S2 Table. Data extraction summary

No	Authors	Publication year	Region	Source of data	Study design	Study population	Sample size	Prevalence (%)	SE of prevalence	Name of data extractors	Date of data extraction
1	Melkie et al	2020	Amhara	Primary	Cross-sectional	Children	690	8.30	1.0502657	ZAA	February 10,2024
2	Asmare et al	2023	Amhara	Primary	Cross-sectional	Children	585	35.37	1.9767724	ESC	February 10,2024
3	Tuke et al	2023	SNNP	Primary	Cross-sectional	Children	538	29.20	1.9602746	DTA	February 10,2024
4	Getachew et al	2023	SNNP	Primary	Cross-sectional	Children	1292	44.10	1.3813189	NM	February 10,2024
5	Genet et al	2022	Amhara	Primary	Cross-sectional	Children	704	6.00	0.8950622	ZAA	February 11,2024
6	Alambo et al	2020	SNNP	Primary	Cross-sectional	Children	586	37.90	2.0040871	ESC	February 11,2024
7	Mekonnen et al.	2022	SNNP	Primary	Cross-sectional	Children	178	21.91	3.100337	DTA	February 11,2024
8	Shimelash et al	2022	Amhara	Primary	Cross-sectional	Children	401	9.90	1.4914469	NM	February 11,2024
9	Belsti et al.	2021	SNNP	Primary	Cross-sectional	Children	620	21.6	1.6526811	ZAA	February 12,2024
10	Abdilwohab et al.	2020	SNNP	Primary	Cross-sectional	Children	831	17.80	1.326922	DTA	February 12,2024
11	Ayelgn et al	2021	Amhara	Primary	Cross-sectional	Children	792	11.80	1.146338	NM	February 12,2024
12	Kedir et al	2021	SNNP	Primary	Cross-sectional	Children	589	29.40	1.8772338	ESC	February 12,2024
13	Abdurahmanl	2023	Oromia	Primary	Cross-sectional	Children	1211	22.10	1.1923199	ZAA	February 13,2024
14	Yeshitila et al	2022	Harari	Primary	Cross-sectional	Children	760	27.00	1.6104102	DTA	February 13,2024
15	Delelegn et al	2021	Oromia	Primary	Cross-sectional	Children	746	17.50	1.3911584	NM	February 13,2024

16	Kassaw et al	2020	Tigray	Primary	Cross-sectional	Children	596	22.00	1.696819	ESC	February 13,2024
17	Reda et al	2020	Tigray	Primary	Cross-sectional	Children	502	21.50	1.8335899	ZAA	February 14,2024
18	Seyum et al	2022	SNNP	Primary	Cross-sectional	Children	1082	20.00	1.2160358	DTA	February 14,2024
19	Miecha et al	2023	Oromia	Primary	Cross-sectional	Children	29230	10.10	0.1762488	NM	February 14,2024
20	Nash et al	2023	Amhara	Primary	Cross-sectional	Children	2841	11.90	0.6074714	ZAA	February 15,2024
