# Supplementary material for: Beyond the SAFE strategy: Systematic review and meta-analysis of prevalence and associated factors of active trachoma among children in Ethiopia
Source: PLoS One. 2025 Feb 20;20(2):e0312024. doi: 10.1371/journal.pone.0312024 (PMC11841906; doi:10.1371/journal.pone.0312024)
Supplement: S3 Table — (PDF) [file pone.0312024.s005.pdf]

S3A Table. Data extraction summary for fly eye contact

Articles	Fly eye contact	LB FEC	UB FEC	LogLB FEC	LogUB FEC	Logor FEC	Slogor FEC	Name of data extractors	Date of data extraction
Melkie et al	3.14	1.43	6.89	0.357674	1.93007	1.14422	0.4011216	ZAA	February 10,2024
Asmare et al	1.96	1.09	3.53	0.086178	1.2613	0.67294	0.2997756	ESC	February 10,2024
Getachew et al	16.3	9.93	26.6	2.29556	3.28241	2.78871	0.2517483	NM	February 10,2024
Genet et al	2.59	1.11	6.03	0.10436	1.79675	0.95166	0.4317314	ZAA	February 11,2024
Mekonnen et al	3.42	1.43	8.17	0.357674	2.10047	1.22964	0.4445904	DTA	February 11,2024
Abdilwohab et al	2.87	1.69	6.46	0.524729	1.86563	1.05431	0.3420665	DTA	February 12,2024
Yeshitila et al	2.15	1.46	3.16	0.378436	1.15057	0.76547	0.1969734	DTA	February 13,2024
Delelegn et al	6.47	3.36	12.4	1.211941	2.52092	1.86718	0.3339225	NM	February 13,2024
Kassaw et al	4.6	2.1	9.9	0.741937	2.29253	1.52606	0.3955606	ESC	February 13,2024

Note: LB: lower bound, UB:upper bound, FEC: fly eye contact

S3B Table. Data extraction summary for facial uncleanliness

Articles	facial uncleanliness	LB UF	UBUF	LogLB UF	LogUB UF	Logor UF	Slogor UF	**Name of data extractors**	**Date of data extraction**
Melkie et al	2.44	1.08	5.5	0.076961	1.7047481	0.891998	0.4152518	ZAA	February 10,2024
Mekonnen et al	3.99	1.42	11.15	0.3506569	2.4114395	1.3837912	0.5257099	DTA	February 11,2024
Shimelash et al	23.9	8.25	69.38	2.1102132	4.2395986	3.1751329	0.5432106	NM	February 11,2024
Abdilwohab et al	3.5	2.12	5.97	0.7514161	1.7867469	1.252763	0.264115	DTA	February 12,2024
Yeshitila et al	4.11	2.46	6.87	0.9001613	1.9271641	1.413423	0.2619905	DTA	February 13,2024
Reda et al	18.2	4.93	69.32	1.595339	4.2387335	2.9025199	0.6743353	ZAA	February 14,2024

Note: LB: lower bound, UB:upper bound,UF: unclean face

S3C Table. Data extraction summary for latrine un-utilization

Articles	latrine un-utilization	LBLU	UBLU	Log LBLU	LogUBLU	LogorLU	SlogorLU	**Name of data extractors**	**Date of data extraction**
Asmare et al	5.28	2.88	9.7	1.0577903	2.2721259	1.6639261	0.3097795	ESC	February 10,2024
Tuke et al	3.7	1.6	8.6	0.4700036	2.1517622	1.3083328	0.42902	DTA	February 10,2024
Genet et al	4.29	1.96	9.34	0.6729445	2.2343063	1.4562867	0.3983066	ZAA	February 11,2024
Alambo et al	6.88	2.13	22.2	0.756122	3.099191	1.9286187	0.5977217	ESC	February 11,2024

Shimelash et al	5.18<br>4.12	1.96<br>2	13.7<br>8.51	0.6729445<br>0.6931472	2.6166656<br>2.1412419	1.6448051<br>1.4158532	0.4958472<br>0.3694119	NM	February 11,2024
Belsti et al	4.12	2	8.51	0.6931472	2.1412419	1.4158532	0.3694119	ZAA	February 12,2024
Abdilwohab et al	2.5	1.63	3.94	0.48858	1.3711807	0.9162907	0.2251532	DTA	February 12,2024
Ayelgn et al	3.2	1.5	6.7	0.4054651	1.9021075	1.1631508	0.3817965	NM	February 12,2024
Yeshitila et al	1.22	1.01	1.47	0.0099503	0.3852624	0.1988509	0.0957429	DTA	February 13,2024
Delelegn et al	2.52	1.5	4.1	0.4054651	1.410987	0.9242589	0.2565107	NM	February 13,2024

Note: LB: lower bound, UB:upper bound, LU:latrine un-utilization

S3D Table. Data extraction summary for water source

Articles	water source	LBWS	UBWS	LogLBWS	LogUBWS	LogorWS	SlogorWS	**Name of data extractors**	**Date of data extraction**
Asmare et al	3.89	1.3	11.67	0.2623643	2.4570214	1.3584092	0.5598615	ESC	February 10,2024
Getachew et al	1.47	1.33	1.67	0.2851789	0.5128236	0.3852624	0.0580726	NM	February 10,2024
Ayelgn et al	4.7	2.5	8.9	0.9162907	2.1860513	1.5475625	0.3239185	NM	February 12,2024
Kedir et al	3.5	1.9	6.5	0.6418539	1.8718022	1.252763	0.3137623	ESC	February 12,2024

Note: LB: lower bound, UB:upper bound, WS:water source
