# Supplementary material for: Beyond the SAFE strategy: Systematic review and meta-analysis of prevalence and associated factors of active trachoma among children in Ethiopia
Source: PLoS One. 2025 Feb 20;20(2):e0312024. doi: 10.1371/journal.pone.0312024 (PMC11841906; doi:10.1371/journal.pone.0312024)
Supplement: S4 Table — (PDF) [file pone.0312024.s006.pdf]

S4 Table. Eligibility confirmation

No	Included Articles	Was the study conducted in Ethiopia?	Was the study observational?	Was the study conducted among children?	Was the study published between the years 2019 and 2024?	Was the study published in English?	Eligibility confirmed
1	Melkie et al	Yes	Yes	Yes	Yes	Yes	Yes
2	Asmare et al	Yes	Yes	Yes	Yes	Yes	Yes
3	Tuke et al	Yes	Yes	Yes	Yes	Yes	Yes
4	Getachew et al.	Yes	Yes	Yes	Yes	Yes	Yes
5	Genet et al	Yes	Yes	Yes	Yes	Yes	Yes
6	Alambo et al	Yes	Yes	Yes	Yes	Yes	Yes
7	Mekonnen et al	Yes	Yes	Yes	Yes	Yes	Yes
8	Shimelash et al	Yes	Yes	Yes	Yes	Yes	Yes
9	Belsti et al	Yes	Yes	Yes	Yes	Yes	Yes
10	Abdilwohab et al	Yes	Yes	Yes	Yes	Yes	Yes
11	Ayelgn et al	Yes	Yes	Yes	Yes	Yes	Yes
12	Kedir et al	Yes	Yes	Yes	Yes	Yes	Yes
13	Abdurahmanl	Yes	Yes	Yes	Yes	Yes	Yes
14	Yeshitila et al	Yes	Yes	Yes	Yes	Yes	Yes
15	Delelegn et al	Yes	Yes	Yes	Yes	Yes	Yes
16	Kassaw et al	Yes	Yes	Yes	Yes	Yes	Yes
17	Reda et al	Yes	Yes	Yes	Yes	Yes	Yes
18	Seyum et al	Yes	Yes	Yes	Yes	Yes	Yes
19	Miecha et al	Yes	Yes	Yes	Yes	Yes	Yes
20	Nash et al	Yes	Yes	Yes	Yes	Yes	Yes
