# Supplementary material for: Beyond the SAFE strategy: Systematic review and meta-analysis of prevalence and associated factors of active trachoma among children in Ethiopia
Source: PLoS One. 2025 Feb 20;20(2):e0312024. doi: 10.1371/journal.pone.0312024 (PMC11841906; doi:10.1371/journal.pone.0312024)
Supplement: S5 Table — (PDF) [file pone.0312024.s007.pdf]

S5A Table. Quality assessment by using the JBI critical appraisal tool for analytical cross-sectional studies.

Analytical cross-sectional studies	Were the criteria for inclusion in the sample clearly defined?	Were the study subjects and the setting described in detail?	Was the exposure measured in a valid and reliable way?	Were objective, standard criteria used for measurement of the condition?	Were confounding factors identified?	Were strategies to deal with confounding factors stated?	Were the outcomes measured in a valid and reliable way?	Was appropriate statistical analysis used?	rate of "yes" (#yes /8)×100
Melkie et al	yes	yes	yes	yes	yes	yes	No	No	75.00
Asmare et al	yes	yes	yes	yes	yes	yes	yes	No	87.50
Tuke et al	yes	yes	yes	yes		yes	yes	yes	87.50
Getachew et al	No	yes	yes	yes	yes	yes	yes	yes	87.50
Genet et al	yes	yes	yes	yes	No	No	yes	yes	75.00
Alambo et al	yes	yes	No	No	yes	yes	yes	yes	75.00
Mekonnen et al	No	yes	yes	yes	yes	yes	yes	yes	87.50
Belsti et al	No	No	yes	yes	yes	yes	yes	yes	75.00
Abdilwohab et al	yes	yes	yes	No	No	yes	yes	yes	75.00
Ayelgn et al	yes	No	yes	yes	yes	yes	yes	No	75.00
Kedir et al	yes	yes	yes	yes	No	yes	yes	yes	87.50
Abdurahmanl	yes	yes	yes	yes	yes	No	No	yes	75.00
Yeshitila et al	No	yes	yes	yes	yes	yes	yes	yes	87.50
Delelegn et al	No	yes	yes	yes	yes	yes	No	yes	75.00
Kassaw et al	yes	yes	No	yes	No	yes	yes	yes	75.00
Reda et al	yes	yes	yes	yes	yes	yes	No	yes	87.50
Seyum et al	yes	yes	yes	yes	No	yes	No	yes	75.00
	No	No	yes	yes	yes	yes	yes	yes	75.00

j

S5B Table. Quality assessment by using the JBI critical appraisal tool for descriptive cross-sectional studies.

Descriptive cross-sectional studies	Was the sample frame appropriate to address the target population?	Were study participants sampled in an appropriate way?	Was the sample size adequate?	Were the study subjects and the setting described in detail?	Was the data analysis conducted with sufficient coverage of the identified sample?	Were valid methods used for the identification of the condition?	Was the condition measured in a standard, reliable way for all participants?	Was there appropriate statistical analysis?	Was the response rate adequate, and if not, was the low response rate managed appropriately?	rate of "yes" (#yes /9)×100
Miecha et al	yes	yes	No	yes	yes	yes	yes	v	yes	87.50
Nash et al	yes	yes	yes	No	yes	No	yes	yes	yes	75.00
